# Supplementary material for: Data-mining of potential antitubercular activities from molecular ingredients of traditional Chinese medicines
Source: PeerJ. 2014 Jul 17;2:e476. doi: 10.7717/peerj.476 (PMC4106188; doi:10.7717/peerj.476)
Supplement: Table S2 — Supplementary Table 2 shows the molecules predicted to have anti tubercular activity by our models. [file peerj-02-476-s002.docx]

[13CH3]C1CCC(=C([13CH3])[13CH3])C(=O)C1 7205

[13CH3]N1C=NC2=C1C(=O)NC(=O)N2[13CH3] 8551

[2H][C@@]1(C[C@@H](O[C@@H]1CO)N2C=C(C(=O)NC2=O)C)O 8572

[2H]C([2H])([2H])C(=O)C([2H])([2H])CCCCC=C 10990

[2H]C([2H])(CC)CCCCCCCC=C 10953

[2H]C(CC=O)C1=CC=CC=C1 10983

[2H]C1=C(C(=C(C(=C1[2H])[2H])C(=O)C)[2H])[2H] 58

[2H]C1=C(C(=C(C(=C1CCN)[2H])O)O)[2H] 2593

[2H]C1=C(C(=C(C2=C1C(=C(C(=O)O2)[2H])[2H])[2H])O)[2H] 8844

[2H]C1=C(C(=C2C(=C1[2H])C3=C(C(=C(C4=C3C2=C(C(=C4[2H])[2H])[2H])[2H])[2H])[2H])[2H])[2H] 9367

[2H]C1=CC2=C(C(=C1OC)[2H])C(=C(N2)[2H])CCNC(=O)C 10961

[3H]C1=CC(=CC2=C1NC3=C2CCN4C[C@@H]5C[C@@H]([C@H]4[C@H]3C5)CC)OC 4101

[C@@H]1([C@@H]([C@H](O[C@H]([C@@H]1O)O)C(O)O)O)O 10969

[C-]#[N+][C@@H](C1=CC=CC=C1)O[C@H]2[C@@H]([C@H]([C@@H]([C@H](O2)CO)O)O)O 7109

[CH]1[CH][C]2[CH][C]=[C][C]=[C][C]2[CH]1 794

[CH2-][C@]12CCC(=[O+]C3=[O+]C(=C)C(=C(C3)[O-])[O+]=C4CC(=C(C(=C)O4)[O+]=C5CC(=O)C(=O)C(=C)O5)[O-])C[C@H]1CC[C@@H]6[C@@H]2CC(=O)[C@]7([C@@]6(CC[C]7C8=C=C(OC8)[O-])[O-])[CH2+] 2347

[Fe+2] 9549

C([C@@H]([C@H]([C@@H]([C@H](CO)O)O)O)O)O 2621

C([C@@H](C(=O)[O-])[NH3+])C(=O)N 686

C([C@@H]1[C@@H]([C@@H]([C@H]([C@@H](O1)O[C@H]2[C@H](OC([C@@H]([C@H]2O)O)O[C@H]3[C@H](O[C@H]([C@@H]([C@H]3O)O)O[C@H]4[C@H](O[C@H]([C@@H]([C@H]4O)O)O[C@H]5[C@H](O[C@H]([C@@H]([C@H]5O)O)O[C@]6([C@H]([C@@H]([C@H](O6)CO)O)O)CO)CO)CO)CO)CO)O)O)O)O 5073

C([C@@H]1[C@@H]([C@@H]([C@H]([C@@H](O1)OCC(COC=O)OC=O)O)O)O)O 5779

C([C@@H]1[C@H]([C@@H]([C@@](O1)(CO)O)O)O)O 10908

C([C@H]([C@H]([C@H]([C@@H](C(=O)CO)O)O)O)O)O 3187

C(C(=CC#N)CO)O 1879

C(C(=O)[O-])(NC(=O)N)NC(=O)N 10301

C(C[C@H](C(=O)O)N)CNC(=O)N 1558

C(CC(C(=O)[O-])[NH3+])C[NH3+] 6373

C(CC[NH+]=C(N)N)C[NH3+] 231

C(CCN)CN.Cl 7221

C.CC(C)C12C(O1)C(C3=C(C2O)C(=O)CC4C3(CCC5=C4COC5=O)C)O 8739

C/[N+](=N/COC1C(C(C(C(O1)CO)O)O)O)/[O-] 1882

C/C(=C/COC(=O)C)/C(=O)O[C@@H]1C[C@@]2([C@H](O2)[C@@H]3[C@@H](O3)/C(=C\[C@@H]4[C@@H]1C(=C)C(=O)O4)/CO)C 2674

C/C(=C\C[C@H]1C(=CCC2[C@@]1(CCCC2(C)C)C)C=O)/C=C 4737

C/C(=C\C[C@H]1C(=CCC2[C@@]1(CCCC2(C)C)C)CO)/C=C 4738

C/C(=C\C=C\C=C(\C=C\C=C(\C(=O)[O-])/C)/C)/C=C/C=C(/C(=O)[O-])\C 9294

C/C(=C\C1=CC(=C(C=C1)OC)OC)/C2(C=C(C(=O)C=C2OC)CC=C)OC 9083

C/C(=C\CC(/C(=C/CCC1(C(/C(=C(/C)\C=O)/CC[C@]1(C)O)CCCO[C@H]2[C@@H]([C@H]([C@@H]([C@H](O2)CO)O)O)O)C)/C)O[C@H]3[C@@H]([C@H]([C@@H]([C@H](O3)CO)O)O)O)/CCC(C(C)(C)O)O 4198

C/C(=C\CC/C(=C/CC1=C(C=CC(=C1)C(=O)O)O)/C)/CCC(C(C)(C)O)O 4674

C/C(=C\CC1=C(C=C(C2=C1OC=C(C2=O)C3=CC(=C(C=C3)OC)O)O)O)/CO 3216

C/C(=C\CC1=C(C=C(C2=C1OC=C(C2=O)C3=CC=C(C=C3)O)O)O)/CO 3215

C/C/1=C\C/C=C/C/C(=C/CC1)/C 7018

C/C/1=C\C2C(C(C(/C(=C\CC1)/C=O)O)OC(=O)C(C)C)C(=C)C(=O)O2 3964

C/C/1=C\C2C(C(C(/C(=C\CC1)/CO)OC(=O)C(C)(C)O)OC(=O)C(C)C)C(=C)C(=O)O2 43

C/C/1=C\CC/C(=C\C[C@]2([C@@H](C/C=C(/[C@@H](CC1)O)\C)C(=C(C2=O)O)[C@H](C)COC(=O)C)C)/C 7062

C/C/1=C\CC[C@@](/C=C/[C@@H](CC/C(=C\CC1)/C)C(C)C)(C)O 4271

C/C/1=C\CC[C@@]23[C@@H](O2)[C@H](C4=C(C1)OC=C4C)OC3=O 4940

C/C/1=C\CCC2([C@H](O2)C(=O)C3=C(C1)OC=C3C)C 9265

C/C=C(/C)\C(=O)O[C@@H]1[C@H]2C3[C@](CO2)(C(CC([C@@]3(C4[C@@]1(C5=C([C@@H](CC5OC(C4)O)C6=CC=CO6)C)C)C)OC(=O)C)OC(=O)C)C 6128

C/C=C(/C)\C(=O)O[C@H]1[C@@H]([C@@H]2[C@@H]([C@]3(C(=CC(=O)C3=C1C)C)O)OC(=O)C2=C)O 7207

C/C=C(/C=O)\[C@@H]1CC(=O)OC=C1OC(=O)C 2678

C/C=C(/C=O)\C1=CC=CC=C1 6732

C/C=C(/CC[C@@H](C)C1CCC2[C@@]1(CCC3C2=CC[C@@H]4[C@@]3(CC[C@@H]([C@H]4C)O)C)C)\C(C)C 1557

C/C=C(/CO)\C(=O)O[C@@H]1C/C(=C/[C@H](C/C(=C/[C@@H]2[C@@H]1C(=C)C(=O)O2)/C)O)/C 2022

C/C=C(\C)/C(=O)O[C@H]1[C@@H](C(OC2=C1C=C3C=CC(=O)OC3=C2)(C)C)OC(=O)/C(=C/C)/C 9144

C/C=C(\C)/C(=O)O[C@H]1[C@@H](C(OC2=C1C3=C(C=C2)C=CC(=O)O3)(C)C)O 7239

C/C=C(\C)/C(=O)O[C@H]1C/C(=C\C(=O)/C=C(/[C@@H]([C@H]2[C@@H]1C(=C)C(=O)O2)O)\C)/C 5762

C/C=C(\C)/C(=O)OC1CC(C2C(C=C(C2(C3C1C(=C)C(=O)O3)O)C)OC(=O)C)(CCl)O 2945

C/C=C(\C)/C(=O)OC1CC2(CO2)C3C(C4C(C3(C5C1C(=C)C(=O)O5)O)(O4)C)O 2976

C/C=C(\CO)/C(=O)OC/C(=C\CO)/C(=O)OC1C/C(=C\CC(/C(=C\C2C1C(=C)C(=O)O2)/C)OC(=O)C)/C 7107

C/C=C/1\[C@@H](C(=CO[C@H]1O[C@H]2[C@@H]([C@H]([C@@H]([C@H](O2)CO)O)O)O)C(=O)OC)CC(=O)OC[C@H]([C@H]3CC[C@@H]([C@@H]3C(=O)O)C)C(=O)O 2725

C/C=C/1\[C@@H]2CC(=O)OC[C@@H](OC[C@@H]3[C@H]([C@@H]([C@H]([C@@H](O3)O[C@@H]1OC=C2C(=O)OC)O)O)O)C4=CC(=C(C=C4)O)O 3127

C/C=C/1\C[C@H]([C@](C(=O)OCC2=CCN3C2[C@@H](CC3)OC1=O)(CO)O)C 8885

C/C=C/1\C2/C/3=C/N4C5=CC=CC=C5C67C4/C(=C\N8C3C9(C3=CC=CC=C83)C(C2)N(C1)CC9)/C1/C(=C\C)/CN(C6C1)CC7 2395

C/C=C/1\CN2C3CC(C2=O)(C4CC1C3CO4)C5=C(C=C(C=C5)O)COC 5411

C/C=C/1\CN2C3CC(C2=O)(C4CC1C3CO4)C5=CC=CC=C5NOC 3265

C/C=C/1\CN2CCC34C2(C(=O)CC1C3C(=O)OC)N(C5=CC=CC=C45)C 9017

C/C=C/C(=O)OC1CC2[C@@]3(C1(C4(CC(=O)C(=CC4O2)C)C)C)CO3 8649

C/C=C/C1=CC(=C(C=C1)O[C@@H](C)[C@H](C2=CC(=C(C(=C2)OC)OC)OC)O)OC 8175

C/C=C/C1=CC=C(C=C1)OCC=C(C)C 2504

C/C=C/C1=CC2=C(C(=C1)OC)O[C@H]([C@@H]2C)C3=CC(=C(C=C3)O)OC 4876

C/C=C/C1=CC2=C(C=C1)OC(=C2C)C3=CC(=CC=C3)OC4=C(C=CC(=C4)C5=C(C6=C(O5)C=CC(=C6)/C=C/C)C)O 2536

C/C=C\1/[C@@H](OC=C(C1CC(=O)OC[C@@H]2[C@H]([C@@H]([C@H]([C@@H](O2)OCCC3=CC=C(C=C3)O)OC(=O)CC\4C(=CO[C@H](/C4=C/C)O[C@H]5[C@@H]([C@H]([C@@H]([C@H](O5)CO)O)O)O)C(=O)OC)O)O)C(=O)OC)O[C@H]6[C@@H]([C@H]([C@@H]([C@H](O6)CO)O)O)O 10724

C/C=C\1/[C@@H]2[C@]3(C=C[C@@H]4[C@H]3[C@H](O2)OC=C4C(=O)OC)OC1=O 4391

C/C=C\1/C(=O)C[C@@]2([C@@]1(CCC34C2CCC5C3(C4)CCC(C5(C)C)N(C)C)C)C 1109

C/C=C\1/C[N@@+]2(CC[C@@]34[C@@]2(CCC1C3(CO)C(=O)OC)NC5=CC=CC=C45)C 2654

C/C=C\1/C2[C@@]3(C=C[C@H]4[C@@H]3[C@@H](O2)OC=C4C(=O)OC)OC1=O 6927

C/C=C\1/CN2[C@@H]3C[C@]45C(C2CC1[C@]3(C4O)C(=O)OC)NC6=CC=CC=C56 9013

C/C=C\1/CN2[C@H]3C[C@H]1[C@H]([C@@H]2CC4=C3NC5=C4C=C(C=C5)O)CO 7667

C/C=C\1/CN2C3CC1C(C45C3(NC6=CC(=C(C=C64)OC)OC)OC2C5)C(=O)OC 7250

C/C=C\1/CN2CC[C@H]1C(=C)C3=C(C2)C=C4C=CNC4=C3 8209

C/C=C\1/CN2CCC3=C(C2CC1C(CC4=C[N+]5=C(CC6C(C5)C(OC=C6C(=O)OC)C)C7=C4C8=CC=CC=C8N7)C(=O)OC)NC9=CC=CC=C39 7824

C/C=C\C(=O)O[C@H]1[C@@H]([C@@]2([C@@H](C[C@@]3(C(=CC[C@H]4[C@]3(CCC5[C@@]4(CC[C@@H](C5(C)C)O[C@H]6[C@@H]([C@H]([C@@H]([C@H](O6)C(=O)O)O)O[C@H]7[C@@H]([C@H]([C@H](CO7)O)O)O[C@H]8[C@@H]([C@H]([C@@H](CO8)O)O)O)O[C@H]9[C@@H]([C@H]([C@H]([C@H](O9)CO)O)O)O)C)C)[C@@H]2CC1(C)C)C)O)CO)OC(=O)C 3083

C/C=C\CC1C(C2=C(C=CC3=C2OC(=O)C=C3)OC1(C)C)OC(=O)C 7009

C[11CH](C)NCC(COC1=CC=C(C=C1)CC(=O)N)O 10695

C[C@@]1(CC[C@@]2(CC[C@@]3(C(=CC[C@H]4[C@]3(CC[C@@H]5[C@@]4(C[C@@H]([C@@H](C5(C)CO)O[C@H]6[C@@H]([C@H]([C@@H](CO6)O)O)O)O)C)C)[C@@H]2C1)C)C(=O)O)C(=O)OC 6791

C[C@@]1(CC[C@@]23CO[C@]4([C@@H]2C1)CC[C@@H]5[C@]6(CC[C@@H](C([C@@H]6CC[C@]5([C@@]4(C[C@H]3O)C)C)(C)C)O[C@H]7[C@@H]([C@H]([C@H](CO7)O[C@H]8[C@@H]([C@H]([C@@H]([C@H](O8)CO)O)O)O)O)O[C@H]9[C@@H]([C@H]([C@@H]([C@H](O9)CO)O)O)O)C)C=O 1884

C[C@@]1(CC[C@@H]2C(=C1)CC[C@H]3C2(CCC[C@]3(C)CO)C)C(=O)CO 2470

C[C@@]1(CC[C@@H]2C(=CC1)CCC2(C)C)C=C 7614

C[C@@]1(CC[C@]2([C@@H]1C(OC=C2C(=O)OC)O[C@H]3[C@@H]([C@H]([C@@H]([C@H](O3)CO)O)O)O)O)O 4192

C[C@@]12[C@@H]3[C@H]([C@@H]([C@@H]1O2)O)C(=CO[C@H]3O)C(=O)OC 4763

C[C@@]12C(O1)[C@H]([C@]3(C2C(OC=C3C(=O)OC)O[C@H]4[C@@H]([C@H]([C@@H]([C@H](O4)CO)O)O)O)O)O 7840

C[C@@]12C[C@@H](O[C@@]13C[C@@H]([C@H]4[C@H]2C[C@@H]5C[C@H]4C(=O)O5)OC3=O)C6=COC=C6 2539

C[C@@]12C[C@H]([C@@H]([C@]1(CC(=O)[C@@]3([C@H]2CC=C4[C@H]3C=C(C(=O)C4(C)C)O[C@H]5[C@@H]([C@H]([C@@H]([C@H](O5)CO)O)O)O)C)C)[C@@](C)(C(=O)CCC(C)(C)O)O)O 1044

C[C@@]12C[C@H](C3(CCC(CC3C1=CCC4C2(CCC5C4(CCC(C5(C)C)O)C)C)(C)C)C(=O)O)O 9557

C[C@@]12CC/C=C(\CC[C@@H]3[C@@H]([C@H]1O2)OC(=O)C3=C)/CO 5303

C[C@@]12CC[C@@]3(C(=CCC4[C@]3(CCC5C4(CCC(C5(C)C(=O)O)O)C)C)[C@@H]1CC(CC2)(C)C)C 11279

C[C@@]12CC[C@@H]3[C@@]([C@H]1CC=C4[C@]2(CC[C@@]5([C@H]4CC(CC5)(C)C)C(=O)O)C)(C[C@H]([C@@H](C3(C)C)O)O)C 10728

C[C@@]12CC[C@H]([C@@]([C@H]1CCC(=C)[C@H]2C/C=C\3/C(COC3=O)O)(C)CO)O 11089

C[C@@]12CC[C@H]3C(=O)O[C@H](C[C@]3([C@@H]1[C@H]4[C@H]5[C@@H]([C@@]2(C(=O)O4)O)O5)C)C6=COC=C6 1414

C[C@@]12CCC(=O)C([C@H]1CC[C@]3([C@H]2CC[C@](O3)(C)[C@@H](CCl)O)C)(C)C 209

C[C@@]12CCC(C34[C@@H]1C[C@H](C56C3CCC([C@H]5O)C(=C)C6=O)OC4OC2)O 7292

C[C@@]12CCC[C@@]3([C@@H]1CCC45C3CCC(C4)[C@](C5)(C)O)COC2 3956

C[C@@]12CCC[C@@H]([C@@]13C[C@@H](CC2)C(O3)(C)C)C(=O)O 11263

C[C@@]12CCC[C@]34[C@@H]1CC[C@]56[C@H]3C[C@H](CC5C4N(C2)C)[C@@]7(C6)CC[C@@]89[C@H]1C8C[C@]2([C@@H]9O7)CCC3=C(C2=C1)C[C@@H]1C[C@]3(CN(C1)C)C 8080

C[C@@]12CCC[C@H]([C@@]13C[C@@H](CC2)C(O3)(C)C)CO 812

C[C@@]12CCC3(CCC(CC3C1=CCC4C2(CCC5C4(CCC([C@@]5(C)CO)O[C@H]6[C@@H]([C@H]([C@@H]([C@H](O6)CO)O)O[C@H]7[C@@H]([C@H]([C@@H]([C@H](O7)CO)O)O)O)O[C@H]8[C@@H]([C@H]([C@@H]([C@H](O8)CO)O)O)O)C)C)(C)C)C(=O)O[C@H]9[C@@H]([C@H]([C@@H]([C@H](O9)CO)O)O)O 545

C[C@@]12CCC3(CCC(CC3C1=CCC4C2(CCC5C4(CCC(C5(C)C)O[C@H]6[C@@H]([C@H]([C@@H]([C@H](O6)CO)O)O[C@H]7[C@@H]([C@H]([C@@H]([C@H](O7)CO)O)O)O)O[C@H]8[C@@H]([C@H]([C@@H]([C@H](O8)CO)O)O)O)C)C)(C)C)C(=O)O[C@H]9[C@@H]([C@H]([C@@H]([C@H](O9)CO)O)O)O 543

C[C@@]12CCCC([C@H]1CCC34C2CCC(C3)[C@@](C4)(CO)OC)(C)C 11688

C[C@@]12CCCC34C1C(=O)CC56C3CC(CC5C4N(C2)CCO)[C@@](C6)(C)O 8046

C[C@@]12CCCC34C1CC(C3N(C2)CCOCC5CC67CCC5CC6C89CCC[C@]1(C8CC7C9N2C1OCC2)C)C12C4CC(CC1)C(=C)[C@H]2O 7204

C[C@@H]([C@@](C(C)C)(C(=O)OC)O)OC(=O)C1=CC=CC=C1 664

C[C@@H]([C@@](C(C)C)(C(=O)OC[C@@H]1CCN2[C@H]1CCC2)O)O 9030

C[C@@H]([C@@H]1[C@]23[C@H](O2)[C@@H]4C5[C@]([C@H]([C@H]6[C@@H](C5(C3=CC(=O)O1)C)O6)O)(C(=O)O4)C)O[C@H]7[C@@H]([C@H]([C@@H]([C@H](O7)CO)O)O)O 4184

C[C@@H]([C@H](C1=CN=C2C(=N1)C(=O)N=C(N2)N)O)O 943

C[C@@H]([C@H]1C(CC2([C@@]1(CC=C3[C@H]2CCC4C(=C3)CC[C@@H](C4(C)C)N(C)C)C)C)O)N 1106

C[C@@H]([C@H]1CC[C@@H]2[C@@]1(CC[C@H]3[C@H]2CC(=O)[C@@H]4[C@@]3(C[C@H]([C@H](C4)O)O)C)C)C(C([C@H](C)C(C)C)O)O 2714

C[C@@H]([C@H]1CC[C@@H]2[C@@]1(CC[C@H]3[C@H]2CO[C@@H]([C@@H]4[C@@]3(C[C@H]([C@H](C4)O)O)C)O)C)[C@H]([C@@H]([C@@H](C)C(C)C)O)O 999

C[C@@H](C(=O)C)O 56

C[C@@H](C1CC[C@@]2([C@@]1(CCC34C2C=CC5C3(C4)CCC(C5(C)C)N(C)C)C)C)N 1111

C[C@@H](CC(C1C(O1)(C)C)O)[C@@H]2CC[C@]3(C2(CCC4C3=CCC5[C@@]4(CCC(C5(C)C)O)C)C)C 2394

C[C@@H](CC1=CCC[C@]([C@@H]1C(=C)C=O)(C)C=C)OC(=O)C2=CC3=C(C4=C5C=CC=C(C5=CC(=C24)[N+](=O)[O-])OC)OCO3 607

C[C@@H]1[C@@H]([C@@H]([C@@H]([C@H](O1)OC2=C(OC3=C(C2=O)C(=CC(=C3CC=C(C)C)O)O)C4=CC=C(C=C4)O)O)O)O 820

C[C@@H]1[C@@H]([C@@H]([C@@H]([C@H](O1)OCC(=O)C2=CC=C(C=C2)OC)O)O)O 4105

C[C@@H]1[C@@H]([C@@H]([C@H]([C@@H](O1)O[C@H]2CC[C@@]3([C@H]4C=CC5=C6CC(CC[C@@]6([C@@H](C[C@@]5([C@@]4(CCC3[C@]2(C)CO)C)C)O)CO)(C)C)C)O)O[C@H]7[C@H]([C@H]([C@@H]([C@H](O7)CO)O)O)O)O 7571

C[C@@H]1[C@@H]([C@@H]([C@H]([C@@H](O1)O[C@H]2CC[C@]3(C([C@]2(C)CO)CC[C@@]4(C3OC=C5[C@]4(CC(C6(C5CC(CC6)(C)C)CO)O)C)C)C)O)O[C@H]7[C@@H]([C@H]([C@@H]([C@H](O7)CO)O)O)O)O 1077

C[C@@H]1[C@@H](CC23C1(CC(=O)O2)CC4([C@@H](C3(CO4)C)C)O)O 7137

C[C@@H]1[C@@H]2C[C@@H]([C@H]3[C@@]4(CCCC([C@H]4C[C@H]([C@]3([C@@H]2O)C1=O)O)(C)C)CO)OC(=O)C 2404

C[C@@H]1[C@@H]2C[C@@H](C2(C)C)CC1=O 4389

C[C@@H]1[C@]23C[C@H]([C@@]14[C@@]5([C@H]2N(CC5)CC=C3)C6=CC=CC=C6N4)C(=O)OC 9021

C[C@@H]1[C@H]([C@@H]([C@@H]([C@H](O1)O[C@@H]2[C@H]([C@@H]([C@H](O[C@@H]2C3=C(C=C4C(=C3O)C(=O)C=C(O4)C5=CC=C(C=C5)OC)OC)CO)O)O)O)O)O 2693

C[C@@H]1[C@H]([C@@H]([C@H]([C@@H](O1)O[C@@H]2[C@H](O[C@H](C[C@@H]2OC)O[C@@H]3[C@H](O[C@H](C[C@@H]3OC)O[C@H]4CC[C@]5(C(C4)CC[C@@]6(C5C[C@H]([C@]7([C@@]6(CC[C@]7([C@H](C)O)O)O)C)O)O)C)C)C)O)OC)O 2605

C[C@@H]1[C@H]([C@@H]([C@H](C(O1)OC[C@@H]2[C@H]([C@@H]([C@H]([C@@H](O2)O[C@@](C)(CCC=C(C)C)C3CC[C@@]4(C3[C@@H](CC5[C@]4(CCC6[C@@]5(CCC(C6(C)C)O[C@@H]7[C@@H]([C@H]([C@@H]([C@H](O7)CO)O)O)O[C@H]8[C@@H]([C@H]([C@@H]([C@H](O8)CO)O)O)O)C)C)O)C)O)O)O)O)O)O 3568

C[C@@H]1[C@H]([C@@H]([C@H](C(O1)OC2CC3[C@]4(CC[C@@H](C(C4CC[C@]3(C5(C2C(CC5)C6(CCC(O6)C(C)(C)O)C)C)C)(C)C)O[C@H]7[C@@H]([C@H]([C@@H](O7)CO)O)O)C)O)O)O 1897

C[C@@H]1[C@H]([C@H]([C@@H]1C2=CC(=C(C=C2OC)OC)OC)C3=CC(=C(C=C3OC)OC)OC)C 951

C[C@@H]1[C@H]([C@H](C[C@@H](O1)O[C@H]2[C@@H](O[C@H](C[C@@H]2OC)O[C@@H]3[C@H](O[C@H]([C@@H]([C@H]3OC)O)O[C@H]4CC[C@@]5([C@H]6CC=C7[C@@H]8[C@@H](CO[C@@]8(OC7=O)C)OC(=O)[C@H]6CC=C5C4)C)C)C)OC)O 753

C[C@@H]1[C@H]([C@H](C[C@@H](O1)O[C@H]2CC[C@@]3([C@@H]4[C@@H](CC=C3C2)[C@]5(CC[C@@H]([C@]5(C([C@H]4O)O)C)[C@@H](C)O)O)C)O)O[C@H]6C[C@@H]([C@@H]([C@H](O6)C)O[C@H]7[C@@H]([C@@H]([C@@H]([C@H](O7)C)O[C@H]8[C@@H]([C@H]([C@@H]([C@H](O8)CO)O)O)O)OC)O)OC 9076

C[C@@H]1[C@H]([C@H](C[C@@H](O1)O[C@H]2CC[C@@]3([C@H]4CC[C@@]5([C@H](CC[C@@]5([C@@H]4CC[C@@]3(C2)O)O)C6=CC(=O)OC6)C)C=O)O)O[C@H]7[C@@H]([C@H]([C@@H]([C@H](O7)CO)O)O)O 2844

C[C@@H]1[C@H]([C@H](C[C@H](O1)O[C@@H]2[C@H](O[C@@H](C[C@@H]2O[C@@H]3C[C@@H]([C@@H]([C@H](O3)C)O)O[C@H]4[C@@H]([C@H]([C@@H]([C@H](O4)CO)O)O)O)O[C@H]5CC[C@]6([C@@H](C5)CC[C@@H]7[C@@H]6CC[C@]8(C7(C[C@@H]([C@@H]8C9=CC(=O)OC9)O)O)C)C)C)O)O 7216

C[C@@H]1[C@H]([C@H](O[C@H]1C2=CC(=C(C=C2)OC)OC)C3=CC(=C(C=C3)OC)OC)C 8947

C[C@@H]1[C@H](C[C@H]2C[C@@]13C=C(C(=O)C=C3O2)OC)C4=CC5=C(C=C4)OCO5 3180

C[C@@H]1[C@H]2C3CC[C@@H]4[C@]5(CC[C@@H](C([C@@H]5CC[C@]4([C@@]3(CC[C@]2(CCC1=C)C)C)C)(C)C)OC(=O)C)C 8277

C[C@@H]1C(=O)C[C@H]2[C@H](C34C1(C2(C)C)[C@H]([C@@H]([C@@]3(CC[C@@H](C4=C)O)C)OC(=O)C)OC(=O)C)OC(=O)C 11893

C[C@@H]1C(=O)CC2[C@@]([C@@]13CC[C@]4(O3)COC=C4)(CCCC2(C)C)C 7021

C[C@@H]1C[C@@]2([C@@H](C2(C)C)[C@H]3C1(C4C=C(C(=O)[C@]4(CC(=C3)CO)O)C)O)OC(=O)C 7082

C[C@@H]1C[C@@]2([C@@H]3[C@](O3)([C@H](O2)O)C)O[C@@H]4[C@H]1[C@]5([C@@H](C[C@@]67C[C@@]68CC[C@@H](C([C@@H]8CC[C@H]7[C@@]5(C4)C)(C)C)O[C@H]9[C@@H]([C@H]([C@@H](CO9)O)O)O)OC(=O)C)C 154

C[C@@H]1C[C@@]2([C@H]3[C@](O3)([C@@H](O2)OC)C)O[C@@H]4[C@H]1[C@]5([C@@H](C[C@@]67C[C@@]68CC[C@@H](C(C8CC=C7[C@@]5(C4)C)(C)C)O[C@H]9[C@@H]([C@H]([C@@H](CO9)O)O)O)OC(=O)C)C 5500

C[C@@H]1C[C@@]2(C[C@H](C(=O)OCC3(C4C[C@H](C5=C([C@]4(CCC3=O)C)C(=O)[C@H]([C@@]6(C1[C@@H]2C(=O)[C@@]56C)C)O)O)C)C)O 3233

C[C@@H]1C[C@@H]([C@@]23CC(=CCC[C@@H]2[C@@]1(CO3)CC/C(=C/CO)/CO)C)O 6997

C[C@@H]1C[C@@H]([C@@H]([C@@]2([C@]13[C@@H]([C@@H](C[C@@H]2OC(=O)C4=CC=CC=C4)C(O3)(C)C)OC(=O)C5=CC=CO5)C)OC(=O)C)OC(=O)C6=CC=CO6 6355

C[C@@H]1C[C@@H]([C@@H]([C@@]2([C@]13[C@@H]([C@@H](C[C@@H]2OC(=O)C4=CC=CO4)C(O3)(C)C)OC(=O)C5=CC=CO5)C)OC(=O)C)OC(=O)C6=CC=CO6 6357

C[C@@H]1C[C@@H]([C@]2([C@@H](O1)O[C@@H]3C[C@@H]4CC[C@@H]5[C@@H]([C@]4(C[C@H]3O2)C=O)CC[C@]6([C@@]5(CC[C@@H]6C7=CC(=O)OC7)O)C)O)OC(=O)C 671

C[C@@H]1C[C@@H](C[C@H]([C@]12CC[C@H](C2)C(=C)C)C=O)O 5002

C[C@@H]1C[C@@H](CC([C@]1(/C=C/[C@@H](C)O[C@H]2[C@@H]([C@H]([C@@H]([C@H](O2)CO)O)O)O)O)(C)C)O 10574

C[C@@H]1C[C@@H]2[C@@H]([C@@H](C(=O)O2)C)C([C@]3([C@H]1C=CC3=O)C)O 6924

C[C@@H]1C[C@]2(CC(C3C(O2)CC4([C@@]3(CC[C@]56C4CCC7[C@]5(C6)CCC(=O)OC7(C)C)C)C)C)OC1=O 7134

C[C@@H]1C[C@]23[C@@H]4CCCN2CCC[C@@H]3[C@@H](C[C@@H]4C1=O)O 473

C[C@@H]1C[C@H]([C@]23COC(=O)[C@]1([C@H]2CC[C@@H]([C@]34CO4)OC(=O)C)C[C@H](C5=COC=C5)O)O 8505

C[C@@H]1C[C@H]2[C@@H](CC(=O)C3=COC(=C23)C1=O)C(C)C 3458

C[C@@H]1C[C@H]2[C@@H]3[C@H]([C@]4([C@@H]1C=CC4=O)C)OC([C@@]3(C(=O)O2)C)(C)O 8438

C[C@@H]1C[C@H]2[C@H]([C@@H]([C@]3([C@H]1[C@H](C[C@H]3O)OC(=O)C)C)OC(=O)C)C(=C)C(=O)O2 1004

C[C@@H]1C[C@H]2[C@H](O[C@]3([C@H]1[C@]4(CC[C@@]56C[C@@]57CC[C@@H](C([C@@H]7CC[C@H]6[C@@]4([C@H]3O)C)(C)C)O[C@H]8[C@@H]([C@H]([C@@H](CO8)O)O)O)C)O2)C(=C)C 1498

C[C@@H]1C[C@H]2CC3=N[C@@H](CCC3[C@@H]4C[C@H]2[C@H](C1)N(C4)C)C[C@H]5C[C@@H](C[C@@H]6[C@H]5CCCN6C(=O)C)C 5007

C[C@@H]1C=C(C(=O)[C@]2([C@H]1C[C@@H]3[C@@]4([C@@H]2[C@@H]([C@H]([C@@]([C@@H]4CC(=O)O3)(C)O)O)O)C)C)OC 6100

C[C@@H]1C=C(C(=O)[C@]2([C@H]1C[C@@H]3[C@@]4([C@@H]2[C@@H]([C@H]([C@@H]([C@@H]4CC(=O)O3)C)OC)OC(=O)C)C)C)OC 6087

C[C@@H]1C=C(C(=O)[C@]2([C@H]1C[C@@H]3[C@@]4([C@@H]2[C@H]5[C@H]([C@@]([C@@]4(CC(=O)O3)O)(C)O)OCO5)C)C)OC 6816

C[C@@H]1C=C(C(=O)[C@]2([C@H]1C[C@@H]3[C@]45[C@@H]2[C@H]([C@@H]([C@]([C@@H]4[C@H](C(=O)O3)OC(=O)CC(C)C)(OC5)C(=O)OC)O)O)C)O[C@H]6[C@@H]([C@H]([C@@H]([C@H](O6)CO)O)O)O 4486

C[C@@H]1C=C(C(=O)C2([C@H]1CC3C4([C@@H]2[C@@H]([C@H]([C@@](C4CC(O3)O[C@H]5[C@@H]([C@H]([C@@H]([C@H](O5)CO)O)O)O)(C)O)OC)O)C)C)OC 6825

C[C@@H]1C=C(O[C@@H]2[C@H]1[C@]3([C@@H](C[C@@]45C[C@@]46CC[C@@H](C([C@@H]6CC=C5[C@@]3(C2)C)(C)C)O[C@H]7[C@@H]([C@H]([C@@H](CO7)O)O)O)OC(=O)C)C)C(=O)C(C)C 677

C[C@@H]1C2=C(CCN1)C3=CC=CC=C3N2 1151

C[C@@H]1CC(=O)[C@@]2([C@]1(CC3=C([C@H]2C)C=CO3)C)C 6854

C[C@@H]1CC[C@@]2([C@H]([C@]3([C@@H](O2)C[C@@H]4[C@@]3(C(=O)C[C@H]5[C@H]4CC[C@H]6[C@@]5(CCC(C6)(OC)OC)C)C)O)C)OC1 685

C[C@@H]1CC[C@@]2([C@H]([C@]3(C(O2)C[C@@]4(C3(CCC5C4CC=C6C5(CCC(C6)O[C@H]7[C@@H]([C@H]([C@@H]([C@H](O7)CO)O[C@H]8[C@@H]([C@H]([C@@H]([C@H](O8)CO)O)O)O)O[C@H]9[C@@H]([C@H]([C@@H](CO9)O)O)O)O[C@H]2[C@@H]([C@@H]([C@H]([C@@H](O2)C)O)O)O)C)C)O)O)C)OC1 6345

C[C@@H]1CC[C@@]2([C@H]([C@H]3[C@@H](O2)C[C@@H]4[C@@]3(CC[C@H]5[C@H]4C[C@@H]6[C@H]([C@]5(C)CC(=O)O)CC(=O)O6)C)C)OC1 7771

C[C@@H]1CC[C@@]2([C@H]([C@H]3[C@@H](O2)C[C@@H]4[C@@]3(CC[C@H]5[C@H]4CC[C@@H]6[C@@]5(CCC(=O)C6)C)C)C)OC1 8582

C[C@@H]1CC[C@@]2([C@H]([C@H]3[C@@H](O2)C[C@@H]4[C@@]3(CC[C@H]5[C@H]4CC[C@H]6[C@@]5([C@@H]([C@@H]([C@H](C6)O)O)O)C)C)C)OC1 8593

C[C@@H]1CC[C@@]2([C@H]([C@H]3[C@@H](O2)C[C@@H]4[C@@]3(CC[C@H]5[C@H]4CC=C6[C@@]5([C@@H](C[C@@H](C6)O)O[C@H]7[C@@H]([C@H]([C@H]([C@H](O7)C)O)O)O[C@H]8[C@@H]([C@@H]([C@H]([C@@H](O8)C)O)OC(=O)C)O)C)C)C)OC1 6346

C[C@@H]1CC[C@@]2([C@H]([C@H]3[C@@H](O2)C[C@@H]4[C@@]3(CC[C@H]5[C@H]4CC=C6[C@@]5([C@@H](C[C@@H](C6)O)O[C@H]7[C@@H]([C@H]([C@H]([C@H](O7)C)O)O[C@H]8[C@@H]([C@H]([C@@H](CO8)O)O)O)O[C@H]9[C@@H]([C@@H]([C@H]([C@@H](O9)C)O)O)O)C)C)C)OC1 6348

C[C@@H]1CC[C@@]2([C@H]([C@H]3[C@@H](O2)C[C@@H]4[C@@]3(CC[C@H]5C4CC(=O)[C@@H]6[C@@]5(CC[C@@H](C6)O)C)C)C)OC1 4832

C[C@@H]1CC[C@@]2(CC[C@@]3(C(=CC[C@H]4[C@]3(CC[C@@H]5[C@@]4(CC[C@@H]([C@@]5(C)CO)O[C@H]6[C@@H]([C@H]([C@H](CO6)O)O)O)C)C)[C@@H]2[C@]1(C)O)C)C(=O)O[C@H]7[C@@H]([C@H]([C@@H]([C@H](O7)CO)O)O)O 4121

C[C@@H]1CC[C@@H]([C@@]2([C@]13[C@@H]([C@@H]([C@H]([C@@H]2OC(=O)C4=CC=CO4)OC(=O)C)C(O3)(C)C)OC(=O)C5=CC=CO5)C)OC(=O)C 6356

C[C@@H]1CC[C@@H]2[C@@]13CC[C@@H](C3)[C@@H](C2(C)C)CO 4496

C[C@@H]1CC[C@@H]2CC3=C(CC[C@]13C2(C)C)C 1940

C[C@@H]1CC[C@]2([C@@]13C[C@@]4([C@]([C@@]2(COC([C@@H]3O)O4)C)(C)O)O)O 1919

C[C@@H]1CC[C@H]2[C@@H]1[C@H]3[C@H](C3(C)C)CC[C@]2(C)O 11091

C[C@@H]1CC=C(C(=O)C1)C(C)(C)O 7728

C[C@@H]1CC2([C@@H]3[C@@](O3)(CO2)C)O[C@@H]4[C@H]1[C@]5(CC[C@@]67C[C@@]68CCC(C([C@@H]8CC[C@H]7[C@@]5(C4)C)(C)C)O[C@H]9[C@@H]([C@H]([C@@H](CO9)O)O)O)C 10862

C[C@@H]1CC2[C@@H](O[C@]3(C1[C@]4(CCC56CC57CC[C@@H](C(C7CCC6[C@@]4([C@H]3O)C)(C)C)O[C@@H]8[C@@H]([C@H]([C@H](CO8)O)O)O)C)O2)C(C)(C)OC 5503

C[C@@H]1CC2[C@H](OC3(C1[C@]4(CC[C@@]56C[C@@]57CC[C@@H](C(C7CCC6[C@@]4([C@H]3O)C)(C)C)O)C)O2)C(C)(C)OC 5502

C[C@@H]1CC2=C(C(=C(C=C2C)OC)O)C(=O)O1 2517

C[C@@H]1CC23C(=C1)/C=C(\C(=O)/C=C\C(CC(=C(C2=O)C)O3)(C)C)/C 4481

C[C@@H]1CCC(C(=O)C1)C(C)C 4369

C[C@@H]1CCC=C2[C@@]1([C@@H]3[C@@H](C3=C)CC2)C 2747

C[C@@H]1CCC2([C@H]([C@H]3[C@@H](O2)C[C@@H]4[C@@]3(CC[C@H]5[C@H]4CC=C6[C@@]5([C@@H](CC(C6)O)OC7[C@@H]([C@@H]([C@@H]([C@H](O7)C)O)O)OC8[C@@H]([C@H]([C@H]([C@@H](O8)C)O)O)O)C)C)C)OC1 6347

C[C@@H]1CCC2([C@H](C3C(O2)CC4[C@@]3(CCC5C4C[C@H]([C@@H]6[C@@]5(C[C@H]([C@@H](C6)O[C@H]7[C@@H]([C@H]([C@@H]([C@H](O7)CO)O[C@@H]8[C@@H]([C@H]([C@H]([C@H](O8)CO)O)O)O)O)O)O)C)O)C)C)OC1 7047

C[C@@H]1CCC2(C(C3C(O2)CC4[C@@]3(CCC5C4CCC6[C@@]5(CC[C@@H](C6)O[C@H]7C([C@H]([C@H]([C@H](O7)CO)O[C@H]8C([C@H](C([C@H](O8)CO)O)O[C@H]9C([C@H](C([C@H](O9)CO)O)O)O)O[C@H]2C([C@H](C(CO2)O)O[C@H]2C(C([C@H](C(O2)C)O)O)O)O)O)O)C)C)C)OC1 2591

C[C@@H]1CCC2(CC[C@@]3(C(=CC[C@H]4[C@]3(CC[C@@H]5[C@@]4(CCC(=O)C5(C)C)C)C)C2[C@H]1C)C)C(=O)O 945

C[C@@H]1CCCC2=CC(=O)C(=C(C)C)C[C@@]12C 2387

C[C@]1([C@H]2CC=C([C@H]2COC1=O)CO)CO 3247

C[C@]1(C[C@@H](C23CCCN4[C@@]2(CCC4)C(=O)CC3[C@@H]1O)O)O 7825

C[C@]1(CC[C@@H](O1)C(C)(C)O)[C@H]2[C@H](C[C@@]3([C@@]2(CC[C@]45[C@H]3CC[C@H]6[C@]4(C5)CC[C@@H](C6(C)C)O[C@@H]7[C@@H]([C@@H]([C@@H](CO7)O)O)O)CO)C)O 855

C[C@]1(CCC23COC4([C@H]2C1)CC[C@@H]5[C@]6(CC[C@@H](C(C6CC[C@]5([C@@]4(C[C@H]3O)C)C)(C)C)O[C@H]7[C@@H]([C@H]([C@H](CO7)O[C@@H]8[C@@H]([C@H]([C@@H]([C@H](O8)CO)O)O)O)O[C@@H]9[C@@H]([C@H]([C@@H]([C@H](O9)CO)O)O)O)O[C@H]1[C@@H]([C@H]([C@@H]([C@H](O1)CO)O)O)O[C@H]1[C@@H]([C@H]([C@@H](CO1)O)O)O)C)C=O 563

C[C@]12C[C@@]3([C@H]4C[C@@]1([C@@]4([C@H](O2)O3)COC(=O)C5=CC=CC=C5)O[C@H]6[C@@H]([C@H]([C@@H]([C@H](O6)COC(=O)C7=CC=CC=C7)O)O)O)O 880

C[C@]12C[C@@]3([C@H]4C[C@@]1(C4([C@@H](O2)O3)COC(=O)C5=CC=C(C=C5)O)O[C@H]6[C@@H]([C@H]([C@@H]([C@H](O6)COC(=O)C7=CC=CC=C7)O)O)O)O 879

C[C@]12C[C@@H](OC(=O)[C@H]1CCC34[C@H]2C=CC=C3C(=O)OC4)C5=COC=C5 4949

C[C@]12C[C@H](C3(C45[C@H]1C(=O)[C@@](O4)([C@@H]6CC=C7CC=CC(=O)[C@@]7(C6CCC5(C(=O)O3)O)C)O)C)OC(=O)C2=C 6781

C[C@]12C[C@H](CC(C1=CC(=O)O2)(C)C)O 11041

C[C@]12CC[C@@](C[C@H]1C3=CC[C@@H]4[C@]5(CC[C@@H]([C@]([C@@H]5CC[C@]4([C@@]3(CC2)C)C)(C)CO)O[C@H]6[C@@H]([C@H]([C@@H]([C@H](O6)C(=O)O)O)O)O[C@H]7[C@@H]([C@H]([C@@H]([C@H](O7)CO)O)O)O)C)(C)C(=O)O 793

C[C@]12CC[C@@H]([C@@]([C@@H]1CC[C@@]3([C@@H]2C[C@@H]4[C@]5([C@]3(C[C@@H]([C@@]6([C@H]5C[C@@]([C@H](C6)OC(=O)C7=CC=CC=C7NC)(C)C=O)C)O)C)O4)C)(C)CO)O[C@H]8[C@@H]([C@H]([C@H](CO8)O[C@H]9[C@@H]([C@H]([C@@H]([C@H](O9)CO)O)O)O)O)O[C@H]1[C@@H]([C@H]([C@@H]([C@H](O1)CO)O)O)O 11260

C[C@]12CC[C@@H]([C@@]3([C@@H]1[C@H](C[C@](O2)(C)C=C)OC(=O)C3)C)C(C)(C)C(=O)OC 212

C[C@]12CC[C@@H]([C@](C1CC[C@@]3(C2CC=C4[C@]3(C[C@H]([C@@]5([C@H]4CC([C@H]([C@@H]5O)O)(C)C)CO)O)C)C)(C)CO)O 7088

C[C@]12CC[C@@H](C([C@@H]1CC[C@@]3([C@@H]2CC=C4[C@]3(C[C@H]([C@@]5([C@H]4CC([C@H]([C@@H]5O)O)(C)C)CO)O)C)C)(C)C)O 831

C[C@]12CC[C@@H](C([C@@H]1CC[C@@]3([C@@H]2CC=C4[C@]3(CC[C@@]5(C4CC(CC5)(C)C)C(=O)O[C@H]6[C@@H]([C@H]([C@H]([C@H](O6)CO)O)O)O)C)C)(C)C)O[C@H]7[C@@H]([C@H]([C@@H]([C@H](O7)C(=O)O)O[C@H]8[C@@H]([C@H]([C@@H](O8)CO)O)O)O)O 548

C[C@]12CC[C@@H](C([C@H]1CC[C@@]3([C@@H]2CC=C4[C@]3(C[C@@H]([C@@]5([C@H]4CC(CC5)(C)C)C)O)C)C)(C)C)O 11004

C[C@]12CC[C@@H](C(C1C(C[C@@]3(C2CCC4C3(CC(C5C4(CC[C@H]5C(C)(C)O)C)O)C)C)O)(C)C)O 5765

C[C@]12CC[C@@H](C(C1CC[C@@]3([C@@H]2CC=C4[C@]3(C[C@H]([C@@]5([C@@H]4CC(C[C@@H]5O)(C)C)CO)O)C)C)(C)C)O 1179

C[C@]12CC[C@@H](C(C1CC[C@@]3(C2CC=C4[C@]3(C[C@H]([C@@]5(C4CC(CC5)(C)C)C(=O)O)O)C)C)(C)C)O[C@H]6[C@@H]([C@H]([C@@H]([C@H](O6)C(=O)OC)O)O)O 2658

C[C@]12CC[C@@H](C(C1CC[C@@]3(C2CC=C4C3(C(C([C@@]5([C@H]4CC(CC5O)(C)C)CO)O)O)C)C)(C)C)O 830

C[C@]12CC[C@@H](C(C1CC=C3C2=CC[C@]4([C@]3(CC[C@@H]4[C@@H](CCC(C(C)(C)O)O)CO)C)C)(C)C)O 3222

C[C@]12CC[C@@H](C(C1CC=C3C2CCC4([C@@]3(CC[C@H]4[C@@H]5C[C@@H](OC5O)C6C(O6)(C)C)C)C)(C)C)O 5311

C[C@]12CC[C@](C[C@H]1C3=CCC4[C@]5(CC[C@@H]([C@](C5CC[C@]4(C3(CC2)C)C)(C)CO)O)C)(C)C(=O)OC 5572

C[C@]12CC[C@H](C1(C)C)C[C@H]2OC(=O)/C=C/C3=CC=CC=C3 981

C[C@]12CC[C@H]3[C@H]([C@H]1CC[C@@H]2C4=COC(=O)C=C4)CCC5[C@@]3(CCCC5)C 1050

C[C@]12CC[C@H]3C([C@]1(CC[C@@H]2C4=COC(=O)C=C4)O)CC[C@]5([C@@]3(CC[C@@H](C5)O)C=O)O 1054

C[C@]12CCC([C@@](C1CC[C@@]3(C2C=C[C@@]45[C@]3(C[C@@H]([C@@]6(C4CC(CC6)(C)C)CO5)O)C)C)(C)CO)O[C@H]7[C@@H]([C@H]([C@@H](CO7)O)O)O 7086

C[C@]12CCC(=O)C([C@@H]1C[C@@H](C3=C2C(=O)C[C@]4([C@]3(C(=O)C[C@@H]4C5(CCC(=O)O5)C)C)C)O)(C)C 3232

C[C@]12CCC(=O)C([C@@H]1CC[C@@]3([C@@H]2[C@@H](C=C4[C@]3(CC[C@@]5([C@H]4CC(C(=O)C5)(C)C)C(=O)O)C)O)C)(C)C 7069

C[C@]12CCC(=O)C([C@@H]1CC[C@@]3([C@@H]2CC=C4[C@]3(CC[C@@]5([C@H]4CC(CC5)(C)C)C(=O)O)C)C)(C)C 9624

C[C@]12CCC(=O)C([C@@H]1CC[C@]34[C@@H]2CC[C@@H](C3)[C@](C4)(CO)O)(C)C 1153

C[C@]12CCC(=O)C(C1CC[C@@]3(C2CC[C@@]45[C@]3(CC(=O)[C@@]6(C4CC(CC6)(C)C)CO5)C)C)(C)C 2692

C[C@]12CCC(=O)C(C1CC[C@@]3(C2CC=C4C3(CC[C@@]5(C4C[C@](CC5O)(C)C(=O)OC)C)C)C)(C)C 7332

C[C@]12CCC(=O)C[C@H]1CC[C@@H]3C2[C@@H](C[C@]4([C@]3(CC[C@@H]4C5=COC(=O)C=C5)O)C)O 3209

C[C@]12CCC(=O)C=C1CC[C@@H]3C2[C@H](C[C@]4([C@H]3CC[C@@H]4C(=O)CO)C)O 1724

C[C@]12CCC(=O)C=C1CC[C@H]3[C@@H]2C(=O)C[C@@]4([C@@H]3CC[C@@]4(C(=O)CO)O)C 1725

C[C@]12CCC[C@](C1CCC(=C)[C@@H]2CCC3=COC=C3)(C)C(=O)OC 5589

C[C@]12CCC3C(C1CC[C@@H]2O)CCC4=CC(=O)CC[C@]34C 8459

C[C@]12CCC3C(C1CCC2[C@@H]4CO[C@]5(C[C@@H]4OC(=O)C5COC)C)CC=C6[C@@]3(C(=O)C=CC6)C 1978

C[C@]12CCCC([C@@H]1CC(=O)C3=C2C(=O)C4C(C3)(O4)C(C)(C)O)(C)C 1150

C[C@]12CCCC([C@@H]1CC[C@@]3([C@@H]2CC=C(C[C@@H]3O)C=O)C=O)(C)C 3189

C[C@]12CCCC(=C)[C@@]1(C[C@H]3[C@@H](C2)OC(=O)C3=C)O 8415

C[C@]12CCCC(C1CC[C@@]3([C@@H]2CC=C(C[C@H]3O)C=O)C=O)(C)C 3190

C[C@]12CCCC3(C1CCC45C3CCC(C4)C(=C)[C@H]5O)[C@H]6N(C2)CCO6 8932

C[C@H](/C=C/[C@H](C)C(C)C)C1CCC2[C@@]1(CCC3C2CC=C4[C@@]3(CC[C@@H](C4)O)C)C 997

C[C@H](C(=C)CCC(C)[C@H]1CCC2([C@@]1(CC[C@]34C2CCC5[C@]3(C4)CC[C@@H](C5(C)C)O)C)C)C(=O)O 10265

C[C@H](C(=O)N[C@H](CC1=CC=C(C=C1)O)C(=O)NCC(=O)N[C@H](CC2=CNC3=CC=CC=C32)C(=O)N[C@H](CCSC)C(=O)N[C@H](CC(=O)O)C(=O)N[C@H](CC4=CC=CC=C4)C(=O)N)NC(=O)[C@@H](CCC(=O)O)NC(=O)[C@@H](CCC(=O)O)NC(=O)[C@@H](CCC(=O)O)NC(=O)[C@H](CCC(=O)O)NC(=O)[C@H](CCC(=O)O)NC(=O)[C@H](CC(C)C)NC(=O)[C@H](CC5=CNC6=CC=CC=C65)NC(=O)[C@@H]7CCCN7C(=O)CNC(=O)[C@H](CCC(=O)O)N 9817

C[C@H](C[C@@H]1C[C@@H](C(=O)O1)CCO)O 3957

C[C@H](CC/C=C(\C)/C(=O)O)[C@H]1CC[C@]2(C1=CC[C@@H]3[C@@]2(CC[C@@H]4[C@@]3(CC[C@@H](C4(C)C)O)C)C)C 3245

C[C@H](CC/C=C(\C)/C(=O)O)[C@H]1CC[C@]2(C1=CC[C@@H]3[C@@]2(CC[C@@H]4[C@@]3(CC[C@@H](C4(C)C)OC(=O)C)C)C)C 3244

C[C@H](CC/C=C(\C)/C=O)[C@H]1CC[C@@]2([C@@]1(CCC3=C2C(=O)C[C@@H]4[C@@]3(CCC(=O)C4(C)C)C)C)C 5004

C[C@H](CC/C=C(\C)/CO[C@H]1[C@@H]([C@H]([C@@H]([C@H](O1)CO[C@H]2[C@@H]([C@H]([C@@H]([C@H](O2)CO)O)O)O)O)O)O)[C@H]3CC[C@@]4([C@@]3(CC(=O)[C@@]5([C@H]4CC=C6[C@H]5CC[C@H](C6(C)C)O[C@H]7[C@@H]([C@H]([C@@H]([C@H](O7)CO)O)O)O)C)C)C 1267

C[C@H](CC[C@@H](C)C(=C)C)[C@H]1CC[C@@]2([C@@]1(CC[C@]34[C@H]2CC[C@@H]5[C@]3(C4)CC[C@@H](C5(C)C)O)C)C 1907

C[C@H](CC[C@H](C(C)(C)O)O)[C@H]1CC[C@@]2([C@@]1(CC=C3C2=CC[C@@H]4[C@@]3(CCC(=O)C4(C)C)C)C)C 3227

C[C@H](CCC(=O)NCCS(=O)(=O)O)[C@H]1CC[C@@H]2[C@@]1(CC[C@H]3[C@H]2[C@H](CC4[C@@]3(CC[C@H](C4)O)C)O)C 9382

C[C@H](CCC(=O)OC)[C@H]1CC(=O)[C@@]2([C@@]1(C(C(=O)C3=C2C(=O)CC4[C@@]3(CC[C@@H](C4(C)C)O)C)OC(=O)C)C)C 5594

C[C@H](CCC(=O)OC)[C@H]1CC(=O)[C@@]2([C@@]1(CC(=O)C3=C2[C@H](C[C@@H]4[C@@]3(CCC(=O)C4(C)C)C)O)C)C 5592

C[C@H](CCC=C(CO)CO)[C@H]1C[C@@H]([C@@]2([C@@]1(CC=C3C2=CCC4[C@@]3(CCC(=O)C4(C)C)C)C)C)O 3223

C[C@H]1[C@@H]([C@H]([C@@H]([C@@H](O1)O[C@H]2CC[C@]3([C@@H](C2)CCC4C3CC[C@]5(C4(CCC5C6=CC(=O)OC6)O)C)C=O)OC(=O)C)OC)O 6685

C[C@H]1[C@@H]([C@H]([C@H]([C@@H](O1)O[C@@H]2[C@H]([C@@H]([C@H](O[C@H]2O[C@@H]3CC4C(C[C@@H](C5[C@@]4(CC[C@@H]5[C@](C)(/C=C/C(C(C)C)O)O[C@H]6[C@@H]([C@H]([C@@H]([C@H](O6)CO)O)O)O)C)O)[C@@]7(C3C(C(CC7)O)(C)C)C)CO)O)O)O)O)O 5177

C[C@H]1[C@@H]([C@H]([C@H]([C@@H](O1)O[C@@H]2[C@H]([C@@H]([C@H](O[C@H]2OC(=O)[C@@]34CC[C@@]5(C(=CCC6[C@]5(CCC7[C@@]6(CCC(C7(C)C)O[C@H]8[C@@H]([C@H]([C@H](CO8)O)O[C@H]9[C@@H]([C@H]([C@@H]([C@H](O9)CO)O)O)O)O[C@H]1[C@@H]([C@@H]([C@@H]([C@@H](O1)C)O)O)O)C)C)C3[C@@H](C(CC4)(C)C)O)C)CO)O)O)O)O)O 4810

C[C@H]1[C@@H]([C@H]([C@H]([C@@H](O1)O[C@@H]2[C@H]([C@@H]([C@H](O[C@H]2OC3=C[C@@H]4C(=CC[C@@H]5[C@]4(C(=O)C[C@]6([C@]5(C[C@@H]7[C@@H]6[C@@](C[C@@H](O7)C(C(C)(C)O)O)(C)O)C)C)C)C(C3=O)(C)C)CO)O)O)O)O)O 4616

C[C@H]1[C@@H]([C@H]([C@H]([C@@H](O1)O[C@@H]2[C@H]([C@@H]([C@H](O[C@H]2OC3=CC4=C(C=C3)C(=O)C=C(O4)C5=CC(=C(C=C5)O)O)CO)O)O)O)O)O 4995

C[C@H]1[C@@H]([C@H]([C@H]([C@@H](O1)O[C@@H]2[C@H]([C@@H](CO[C@H]2OC(=O)C34CCC(CC3C5=CCC6C(C5(CC4O)C)(CCC7C6(CC(C(C7(CO)C(=O)OC)O[C@H]8[C@@H]([C@H]([C@@H]([C@H](O8)CO)O)O)O)O)C)C)(C)C)O)O)O)O)O[C@H]9[C@@H]([C@H]([C@@H](CO9)O)O[C@H]1[C@@H](C(CO1)(CO)O)O)O 5649

C[C@H]1[C@@H]([C@H]([C@H]([C@@H](O1)O[C@@H]2[C@H]([C@@H](O[C@@H]([C@H]2OC(=O)/C=C/C3=CC(=C(C=C3)O)O)CO)OCCC4=CC(=C(C=C4)O)O)O)O[C@H]5[C@H]([C@@H]([C@@H](CO5)O)O)O)O)O 4825

C[C@H]1[C@@H]([C@H]([C@H]([C@@H](O1)O[C@@H]2[C@H]([C@@H](O[C@@H]([C@H]2OC(=O)/C=C/C3=CC(=C(C=C3)O)O)CO[C@H]4[C@@H]([C@H]([C@@H]([C@H](O4)OC)O)O)O)OCCC5=CC(=C(C=C5)O)OC)O)O)O)O 1541

C[C@H]1[C@@H]([C@H]([C@H]([C@@H](O1)O[C@@H]2[C@H]([C@@H](O[C@@H]([C@H]2OC(=O)/C=C/C3=CC(=C(C=C3)O)O)CO[C@H]4[C@@H]([C@H]([C@H]([C@H](O4)CO)O)O)O)OCCC5=CC(=C(C=C5)O)O)O)O)O)O 7217

C[C@H]1[C@@H]([C@H]([C@H]([C@@H](O1)O[C@@H]2[C@H]([C@@H](O[C@@H]([C@H]2OC(=O)/C=C/C3=CC(=C(C=C3)O)OC)CO)OCCC4=CC(=C(C=C4)O)O)O)O)O)O 4865

C[C@H]1[C@@H]([C@H]([C@H]([C@@H](O1)O[C@@H]2[C@H]([C@H](CO[C@H]2O[C@@H]3[C@H]([C@@H]([C@H](O[C@H]3OC4CCC5([C@H]([C@@]4(C)CO)CCC6(C5CC=C7[C@]6(CCC8(C7CC(CC8O)(C)C)C)C)C)C)C(=O)O)O)O)O)O)O)O)O 10781

C[C@H]1[C@@H]([C@H]([C@H]([C@@H](O1)O[C@@H]2[C@H]([C@H](CO[C@H]2OC3CCC4(C([C@]3(C)CO)CCC5(C4CC=C6[C@]5(CCC7(C6CC(CC7)(C)C)C(=O)O)C)C)C)O)O)O)O[C@H]8[C@@H]([C@H]([C@@H](CO8)O)O)O)O 7642

C[C@H]1[C@@H]([C@H]([C@H]([C@@H](O1)O[C@@H]2[C@H](O[C@H]([C@@H]([C@H]2O)O)OC[C@@H]3[C@H]([C@@H]([C@H]([C@@H](O3)OC(=O)[C@]45CC[C@H]([C@@H]4[C@H]6CC[C@@H]7[C@]8(CC[C@H]([C@@]([C@@H]8CC[C@]7([C@@]6(CC5)C)C)(C)CO)O)C)C(=C)C)O)O)O)CO)O)O)O 7206

C[C@H]1[C@@H]([C@H]([C@H]([C@@H](O1)O[C@@H]2[C@H](O[C@H]([C@@H]([C@H]2O)O)OC3CC4(C(CC(C5C4(CCC5C6(CC[C@@H](O6)C(C)(C)O)C)C)O)C7(C3C(C(CC7)O)(C)C)C)C)CO)O)O)O 7126

C[C@H]1[C@@H]([C@H]([C@H]([C@@H](O1)O[C@H]2[C@@H]([C@H](O[C@@H]([C@@H]2O[C@@H]3[C@@H]([C@H]([C@@H]([C@H](O3)CO)O)O)O)OC(=O)[C@@]45CC[C@@]6(C(=CC[C@H]7[C@]6(CCC8[C@@]7(CC[C@@H](C8(C)C)O[C@H]9[C@@H]([C@H]([C@H](CO9)OC(=O)C)OC(=O)C)O)C)C)[C@H]4CC(CC5)(C)C(=O)O)C)CO)O)O)O)O 9229

C[C@H]1[C@@H]([C@H]([C@H]([C@@H](O1)O[C@H]2[C@@H]([C@H](O[C@H]([C@@H]2O)O[C@@H]3[C@H](O[C@H]([C@@H]([C@H]3O)O)OC4CCC5(C(C4(C)CO)CCC6(C5CC=C7C6(CCC8(C7CC(CC8)(C)C)C(=O)O[C@H]9[C@@H]([C@H]([C@@H]([C@H](O9)CO[C@H]1[C@@H]([C@H]([C@@H]([C@H](O1)CO)O)O)O)O)O)O)C)C)C)CO)CO)O)O[C@H]1[C@@H]([C@H]([C@H](CO1)O)O)O)O)O 6075

C[C@H]1[C@@H]([C@H]([C@H]([C@@H](O1)O[C@H]2[C@@H]([C@H](O[C@H]([C@@H]2O)OCCC3=CC(=C(C=C3)O)O)COC(=O)/C=C/C4=CC(=C(C=C4)O)O)O)O)O)O 4230

C[C@H]1[C@@H]([C@H]([C@H]([C@@H](O1)O[C@H]2[C@@H]([C@H](O[C@H]([C@@H]2O[C@H]3[C@@H]([C@@H]([C@@H]([C@@H](O3)C)O)O)O)OC/C(=C\CC/C(=C/CC/C(=C/O[C@H]4[C@@H]([C@H]([C@@H]([C@H](O4)CO)O)O[C@H]5[C@@H]([C@@H]([C@H]([C@@H](O5)C)O)O)O)O[C@H]6[C@@H]([C@@H]([C@H]([C@@H](O6)C)O)O)O)/C)/C)/C)CO)O)O)O)O 5831

C[C@H]1[C@@H]([C@H]([C@H]([C@@H](O1)O[C@H]2[C@@H](CO[C@H]([C@@H]2O)O[C@H]3[C@@H](O[C@H]([C@@H]([C@@H]3O)O)O[C@@H]4[C@H]([C@H]([C@H](O[C@H]4OC(=O)[C@]56CCC(C[C@@H]5C7=CC[C@H]8[C@]([C@@]7(C[C@H]6O)C)(CCC9[C@@]8(C[C@@H]([C@@H]([C@@]9(C)CO)O[C@H]1[C@@H]([C@H]([C@@H]([C@H](O1)CO)O)O)O)O)C)C)(C)C)C)O)O)C)O)O)O)O 9029

C[C@H]1[C@@H]([C@H]([C@H]([C@@H](O1)O[C@H]2[C@@H](O[C@H]([C@@H]([C@@H]2O)O)O[C@@H]3[C@H]([C@@H](O[C@@H]([C@H]3OC(=O)/C=C/C4=CC=C(C=C4)O)CO)OCCC5=CC=C(C=C5)O)O)C)O)O)O 4914

C[C@H]1[C@@H]([C@H]([C@H]([C@@H](O1)O[C@H]2[C@@H](O[C@H]([C@@H]([C@@H]2O)O)OC3=C(OC4=C(C3=O)C(=CC(=C4CC=C(C)C)O[C@H]5[C@@H]([C@H]([C@@H]([C@H](O5)CO)O)O)O)O)C6=CC=C(C=C6)O)C)O)O)O 823

C[C@H]1[C@@H]([C@H]([C@H]([C@@H](O1)OC[C@@H]2[C@H]([C@@H]([C@H]([C@@H](O2)O[C@@](C)(CCC=C(C)C)C3CC[C@@]4(C3C(CC5C4(CCC6C5(CCC(C6(C)C)O[C@H]7[C@@H]([C@H]([C@@H]([C@H](O7)CO[C@H]8[C@@H]([C@@H]([C@H]([C@@H](O8)C)O)O)O)O)O)O[C@H]9[C@@H]([C@H]([C@@H]([C@H](O9)CO)O)O)O)C)C)O)C)O)O)O)O)O)O 3567

C[C@H]1[C@@H]([C@H]([C@H]([C@@H](O1)OC[C@@H]2[C@H]([C@@H]([C@H]([C@@H](O2)OC3=C([O+]=C4C=C(C=C(C4=C3)O[C@@H]5C[C@@H]([C@@H]([C@@H]([C@H]5O)O)O)CO)O)C6=CC(=C(C=C6)O)O)O)O)O)O)O)O 1875

C[C@H]1[C@@H]([C@H]([C@H]([C@@H](O1)OC[C@@H]2[C@H]([C@@H]([C@H]([C@@H](O2)OC3CCC4(C(C3)CCC5C4CCC6(C5CC7C6C(C(O7)(CCC(C)O[C@H]8[C@@H]([C@H]([C@@H]([C@H](O8)CO)O)O)O)O)C)C)C)O)O)O)O)O)O[C@H]9[C@@H]([C@H]([C@H](CO9)O)O)O[C@H]1[C@@H]([C@H]([C@@H]([C@H](O1)CO)O)O)O 703

C[C@H]1[C@@H]([C@H]([C@H]([C@@H](O1)OC[C@@H]2C[C@@H]([C@H]([C@@H](O2)OC3=CC(=C4C(=C3)OC(=CC4=O)C5=CC=C(C=C5)OC)O)O)O)O)OC(=O)CC(C)C)O 4929

C[C@H]1[C@@H]([C@H]([C@H]([C@H](O1)OC2=C(OC3=C(C2=O)C(=CC(=C3)O)[O-])C4=CC=C(C=C4)O)O)O)O 4558

C[C@H]1[C@@H]([C@H]([C@H]([C@H](O1)OC2=C(OC3=CC(=CC(=C3C2=O)O)O)C4=CC(=C(C=C4)O)O)OC(=O)C5=CC(=C(C(=C5)O)O)O)O)O 7269

C[C@H]1[C@@H]([C@H](C[C@@H](O1)O[C@H]2CC[C@]3([C@@H](C2)CC[C@@H]4C3CC[C@]5([C@@]4(C[C@@H]([C@@H]5C6=CC(=O)OC6)OC(=O)C)O)C)C)OC)O 6317

C[C@H]1[C@@H](C(=C)C[C@]2([C@H]1C3CC[C@H]4[C@]([C@@H]3CC2)(CCC5[C@@]4(CC[C@H](C5(C)C)O)C)C)CO)C 8255

C[C@H]1[C@@H](C2=CC(=C(C(=C2C1=O)CO)CCO)C)O 7190

C[C@H]1[C@@H](CC[C@@]1(C)O)[C@@](C)(CCC=C(C)C)O 1916

C[C@H]1[C@@H](CC[C@@H]2[C@@]1(CC[C@H]3[C@]2(CC[C@@]4([C@@]3(CC[C@@]5([C@H]4CC(CC5)(C)C)C)C)C)C)C)O 2726

C[C@H]1[C@@H]2[C@@H]([C@H]3[C@]([C@H]1C(=O)O2)(C(=O)C=C4[C@@]3(C(=O)C=C4C)C)C)O 4824

C[C@H]1[C@@H]2[C@H](CC(=C)[C@@H]3CC4C([C@@H]3[C@H]2OC1=O)(O4)C)OC(=O)C 9033

C[C@H]1[C@@H]2CN3CC[C@@]4([C@H]3C[C@@H]2C(=CO1)C(=O)OC)C5=CC=CC=C5NC4=O 8850

C[C@H]1[C@H]([C@@H]2[C@@H](O2)C3=CC(=O)[C@@]4(CC13C)[C@@](O4)(C)C=O)OC(=O)C 7108

C[C@H]1[C@H]([C@H]([C@H]([C@@H](O1)O[C@@H]2[C@@H]([C@H]([C@@H](O[C@H]2OC3CCC4(C(C3(C)CO)CCC5(C4CC=C6C5(CCC7(C6CC(CC7)(C)C)C(=O)O[C@H]8[C@@H]([C@H]([C@@H]([C@H](O8)CO)O)O)O[C@H]9[C@@H]([C@H]([C@@H]([C@H](O9)CO)O)O)O)C)C)C)C)O)O)O)O)O 5834

C[C@H]1[C@H]([C@H]([C@H]([C@@H](O1)O[C@@H]2[C@H]([C@@H]([C@H](O[C@H]2OC(C)(C)[C@@H](CC[C@@H](C)[C@H]3CC[C@@]4([C@@]3(CC(=O)[C@@]5([C@H]4CC=C6[C@H]5CCC(C6(C)C)O[C@H]7[C@@H]([C@H]([C@@H]([C@H](O7)CO)O)O)O)C)C)C)O)CO)O)O)O)O)O 4618

C[C@H]1[C@H]([C@H]([C@H]([C@@H](O1)O[C@@H]2[C@H]([C@@H](CO[C@H]2OC3CCC4(C5CCC6C7C(CC(OC78CC6(C5(CCC4C3(C)C)C)CO8)C=C(C)C)(C)O)C)O)O[C@H]9[C@@H]([C@H]([C@@H]([C@H](O9)COC(=O)C)O)O)O[C@H]1[C@@H]([C@H]([C@@H](CO1)O)O)O)O)O)O 118

C[C@H]1[C@H]([C@H]([C@H]([C@@H](O1)O[C@@H]2[C@H]([C@@H](O[C@@H]([C@@H]2O[C@H]3C([C@H]([C@@H]([C@H](O3)CO)O[C@H]4[C@@H]([C@H]([C@@H](CO4)O)O)O)O)O)CO)O[C@@H]5[C@H]([C@@H](O[C@H]([C@@H]5O)O[C@@H]6[C@H]([C@H](CO[C@H]6OC7CCC8(C(C7(C)CO)CCC9(C8CC=C1C9(CCC2(C1CC(CC2)(C)C)C(=O)O)C)C)C)O)O)C)O)O)O)O)O 707

C[C@H]1[C@H]([C@H]([C@H]([C@@H](O1)O[C@@H]2[C@H]([C@H](CO[C@H]2OC3CC[C@]4(C(C3(C)C)CC[C@@]5(C4CC=C6[C@]5(CC[C@@]7(C6CC(CC7)(C)C)C(=O)O[C@H]8[C@@H]([C@H]([C@@H]([C@H](O8)CO)O)O)O)C)C)C)O)O)O)O)O 4806

C[C@H]1[C@H]([C@H]([C@H]([C@@H](O1)O[C@@H]2[C@H]([C@H](CO[C@H]2OC3CC[C@]4(C(C3(C)C)CC[C@@]5(C4CC=C6[C@]5(CC[C@@]7(C6CC(CC7)(C)C)C(=O)O[C@H]8[C@@H]([C@H]([C@@H]([C@H](O8)CO)O)O)O)C)C)C)O)O[C@H]9[C@@H]([C@H]([C@@H]([C@H](O9)CO)O)O)O)O)O)O 4807

C[C@H]1[C@H]([C@H]([C@H]([C@@H](O1)O[C@@H]2[C@H](O[C@H]([C@@H]([C@H]2O)O)OC3CCC4(C(C3)CCC5C4CCC6(C5CC7C6C(C(O7)(CCC(C)O[C@H]8[C@@H]([C@H]([C@@H]([C@H](O8)CO)O)O)O)O)C)C)C)CO)O[C@H]9[C@@H]([C@H]([C@@H]([C@H](O9)CO)O)O)O)O)O 700

C[C@H]1[C@H]([C@H]([C@H]([C@@H](O1)O[C@@H]2[C@H](O[C@H]([C@@H]([C@H]2O)O)OC3CCC4(C(C3)CCC5C4CCC6(C5CC7C6C(C(O7)(CCC(C)O[C@H]8[C@@H]([C@H]([C@@H]([C@H](O8)CO)O)O)O)OC)C)C)C)CO)O[C@H]9[C@@H]([C@H]([C@@H]([C@H](O9)CO)O)O)O)O)O 699

C[C@H]1[C@H]([C@H]([C@H]([C@@H](O1)OC[C@@H]2[C@H]([C@@H]([C@H]([C@@H](O2)O[C@@H]3[C@H]([C@@H]([C@H](O[C@H]3OC4CCC5(C(C4(C)C)CCC6(C5CC(C7C6(CCC7C(C)(CCC=C(C)C)O[C@H]8[C@@H]([C@H]([C@@H]([C@H](O8)CO)O)O)O)C)O)C)C)CO)O)O)O)O)O)O)O)O 3569

C[C@H]1[C@H]([C@H]([C@H]([C@@H](O1)OC2=C(C=C(C=C2)/C=C/C(=O)O[C@@H]3[C@H](O[C@@H]([C@@H]([C@H]3O)O)O[C@]4([C@H]([C@@H]([C@H](O4)CO)O)OC(=O)C5=CC=CC=C5)CO)COC(=O)C6=CC=CC=C6)OC)O)O)O 7352

C[C@H]1[C@H]([C@H]([C@H]([C@@H](O1)OC2=C(OC3=CC(=CC(=C3C2=O)O)O)C4=CC(=C(C=C4)O)O)O[C@H]5[C@@H]([C@H]([C@H]([C@H](O5)CO)O)O)O)O)O 7263

C[C@H]1[C@H]([C@H]([C@H]([C@@H](O1)OC2=CC(=C3C(=C2)C(=O)C4=CC(=CC(=C4C3=O)O)C)O)O)O)O 3125

C[C@H]1[C@H]([C@H]([C@H]([C@@H](O1)OCCCC2=CC=C(C=C2)OC(CO)C(C3=CC(=C(C(=C3)OC)O)OC)O)O)O)O 4106

C[C@H]1[C@H]([C@H](C[C@@H](O1)O[C@H]2CC[C@]3([C@@H](C2)CC[C@@H]4[C@@H]3[C@@H](C[C@]5([C@@]4(CC[C@@H]5C6=CC(=O)OC6)O)C)O)C)OC)O 2576

C[C@H]1[C@H](C([C@H]([C@@H](O1)O[C@@H]2CO[C@@H]([C@@H]([C@H]2O)O)OC(=O)[C@]34CCC(CC3C5=CCC6[C@]7(CC[C@@H](C(C7CC[C@]6(C5(CC4)C)C)(C)C)O[C@H]8[C@@H]([C@H]([C@H](CO8)O[C@H]9[C@@H]([C@H]([C@@H]([C@H](O9)CO)O)O)O)O)O)C)(C)C)O)O[C@H]1[C@@H]([C@H]([C@@H](CO1)O)O)O)O 7692

C[C@H]1[C@H]2[C@@H](C[C@@H]3[C@@]2(CC[C@H]4[C@H]3CCC5[C@@]4(CCC(C5)O)C)C)O[C@]16CCC(CO6)C 7671

C[C@H]1[C@H]2[C@H](C[C@@H]3[C@@]2(CC[C@H]4[C@H]3CC=C5[C@@]4(CC[C@@H](C5)O[C@H]6[C@@H]([C@H]([C@@H]([C@H](O6)CO)O[C@@H]7[C@H]([C@H]([C@@H]([C@H](O7)C)O)O)O)O)O[C@@H]8[C@H]([C@H]([C@@H]([C@H](O8)C)O)O)O)C)C)O[C@@]1(CC[C@H](C)CO[C@H]9[C@@H]([C@H]([C@@H]([C@H](O9)CO)O)O)O)O 7092

C[C@H]1[C@H]2[C@H](C[C@@H]3[C@@]2(CC[C@H]4[C@H]3CC=C5[C@@]4(CC[C@@H](C5)OC6C(C(C(C(O6)CO)O)OC7C(C(C(C(O7)CO)O)O)O)OC8C(C(C(C(O8)C)O)O)O)C)C)O[C@@]1(CC[C@@H](C)COC9C(C(C(C(O9)CO)O)O)O)O 7093

C[C@H]1[C@H]2C[C@H]3C(=C)CCC[C@@]3(C[C@H]2OC1=O)C 2383

C[C@H]1C(=O)C=C[C@@H]2[C@@]1(CC[C@H]3[C@]2(CC[C@@]4([C@@]3(CC[C@@]5([C@H]4CC(CC5)(C)C)C)C)C)C)C 3133

C[C@H]1C(=O)N[C@H](C(=O)N2CCC[C@H]2C(=O)N[C@H](C(=O)N[C@H](C(=O)N[C@H](C(=O)N3CCC[C@H]3C(=O)N[C@H](C(=O)N1)C(C)C)CC4=CC=C(C=C4)O)CC(C)C)C(C)C)CC5=CC=CC=C5 5724

C[C@H]1C(CC([C@]2(C1C3=CCC4[C@]5(CCC(=O)[C@@](C5CC[C@]4(C3(CC2)C)C)(C)CO)C)C)OC(=O)C)C(=O)OC 7331

C[C@H]1C(CCC2=CC(=O)C(C[C@]12C)C(=C)C)OC(=O)/C=C\SC 6692

C[C@H]1C(OC2=C(C=C(C=C12)OC)CC=C)C3=CC=C(C=C3)O 2382

C[C@H]1C[C@@H]2CC(=O)C3CCCN4[C@@]3(C1)[C@@H]2CCC4 5072

C[C@H]1C[C@@H]2CCC[C@H]3N2[C@H](C1)C[C@H]4N3C(=O)CCC4 1393

C[C@H]1C[C@]2([C@H]([C@H]1OC(=O)C)[C@H](C(=C)[C@@H]([C@H]([C@H](C([C@@H]3C(=O)[C@H]([C@]2(O3)O)C)(C)C)OC(=O)C)OC(=O)C4=CC=CC=C4)OC(=O)C)OC(=O)C)OC(=O)C 4574

C[C@H]1C[C@]23C(=O)[C@@H]([C@@H](C4C(C4(C)C)[C@@H]([C@@H](/C(=C/[C@@]2([C@H]1O)O3)/C)O)O)O)C 4162

C[C@H]1C=C(C(=C[C@H]2[C@@H]1CC(C2)(C)C)CO)C=O 8935

C[C@H]1C2=CC(=C(C=C2CCN1)O)OC 7596

C[C@H]1C2=CC(=C(C=C2CCN1[C@]3([C@H]([C@@H]([C@@H](CO3)O)O)O)CO)O)O 2461

C[C@H]1C2C(C3[C@@]4([C@]1(CC(=O)O[C@@H]4CC5[C@@]3(C(=O)C(=CC5C)OC)C)O)C)OCO2 6815

C[C@H]1CC(=O)[C@H]2[C@@H]1C(OC=C2C(=O)OC)O[C@H]3[C@@H]([C@H]([C@@H]([C@H](O3)CO)O)O)O 8956

C[C@H]1CC/C=C(/CC(=O)[C@H](CC1=O)C(C)C)\C 1857

C[C@H]1CC[C@@]2([C@H]([C@H]3[C@@H](O2)C[C@@H]4[C@@]3(CC[C@H]5[C@H]4CC[C@H]6[C@@]5(CC[C@@H](C6)O[C@H]7[C@@H]([C@H]([C@H]([C@H](O7)CO)O[C@H]8[C@@H]([C@H]([C@@H]([C@H](O8)CO)O)O)O)O)O[C@H]9[C@@H]([C@@H]([C@H]([C@@H](O9)C)O)O)O)C)C)C)OC1 8457

C[C@H]1CC[C@@]2(CC[C@]3(C(=CCC4[C@]3(CCC5[C@@]4(CC[C@@H](C5(C)C)O[C@H]6[C@H]7[C@@H]([C@@H]([C@@H](O6)C)O)OC(O7)(C)C)C)C)C2[C@H]1C)C(=O)O)C(=O)O 11862

C[C@H]1CC[C@@H]2[C@]13C[C@@H](C(O3)(CC2=C)O)C(C)C 1856

C[C@H]1CC[C@@H]2[C@H]([C@]3([C@]1(C=CC3=O)O)C)OC(=O)C2=C 6569

C[C@H]1CC[C@]2([C@@H](C3[C@@H](O2)C[C@@H]4[C@@]3(CC[C@@H]5[C@H]4CC[C@@H]6[C@@]5(CC[C@@H](C6)O)C)C)C)NC1 8597

C[C@H]1CC2=CC(=C(C(=C2C3=C(C(=C(C=C3C[C@]1(C)O)OC)OC)OC)OC4=CC=CC=C4)OC)OC 873

C[C@H]1CCC/C=C/C2C[C@@H](C[C@H]2[C@@H](/C=C/C(=O)O1)O)O 1000

C[C@H]1CCC[C@H]2[C@@]1([C@@H](C3=C(C2)OC=C3C)OC)C 6691

C[C@H]1CCC=C2C1[C@@H](C(CC2)C(C)(C)O)C 4719

C[C@H]1CCC2[C@@](C3[C@H]([C@@H]([C@@]4([C@H](C3CN2C1)C[C@@]56[C@H]4[C@@H](C[C@H]7[C@@]5(CC[C@@H]([C@]7(O6)O)O)C)O)O)O)O)(C)O 3314

C[C@H]1CCC2[C@@H](C1)C(CC=C2C)C(=C)C 1116

C[C@H]1CCC2=C(CC(C[C@H]12)(C)C)C(=C)C 995

C[C@H]1CCC2C1C3C(C3(C)C)CC[C@@]2(C)O 10966

C[C@H]1CCC2C1C3C(C3(C)C)CC[C@]2(C)O 4836

C[C@H]1CCC2CC3CCC4C(C3CN2C1)CC5C4CC(=O)C6[C@@]5(CCC(C6)O)C 1477

C[C@H]1CCCC2[C@@]1(C[C@]3(C2)C(=C)COC3=O)C 814

C[N+](C)(C)C=C.[OH-] 6074

C[N+](C)(C)CCC1=CNC2=C1C=C(C=C2)[O-] 1058

C[N+](C)(C)CCCC[C@@H](C(=O)[O-])N 4761

C[N+]1(CCC2=C([C@H]1CC3=CC=C(C=C3)OC)C(=C(C=C2)OC)O)C.[Cl-] 6688

C[N+]1(CCC2=CC(=C(C3=C2[C@@H]1CC4=C3C(=C(C=C4)OC)O)OC)OC)C 5342

C[N+]1(CCC2=CNC3=C2C1=C(C=C3)OS(=O)O)C 1060

C[NH+]1CCC2=CC(=C(C=C2[C@H]1CC3=CC(=C(C=C3)OC)O)O)OC 11084

C[S@@](=O)/C=C/[C@@H](C(=O)O)N 5374

C[Se]C[C@@H](C(=O)[O-])[NH3+] 7783

C\1[C@@H](NC(=C/C1=C/C=[N+]2[C@@H](CC3=CC(=C(C=C32)O[C@H]4[C@@H]([C@H]([C@@H]([C@H](O4)CO)O)O)O[C@H]5[C@@H]([C@H]([C@@H]([C@H](O5)CO)O)O)O)O)C(=O)[O-])C(=O)O)C(=O)O 11286

C\1[C@H]([NH2+]C(=C/C1=C/C=[N+]2[C@@H](CC3=CC(=C(C=C32)[O-])OC4[C@@H]([C@H]([C@@H]([C@H](O4)CO)O)O)OC5[C@@H]([C@H]([C@@H]([C@H](O5)C(=O)[O-])O)O)O)C(=O)[O-])C(=O)O)C(=O)[O-] 353

C\1[C@H](NC(=C/C1=C/C=O)C(=O)O)C(=O)O 11268

C\1C(NC(=C/C1=C\C=[N+]2C(CC3=CC(=C(C=C32)O)OC4C(C(C(C(O4)CO)O)O)O)C(=O)[O-])C(=O)O)C(=O)O 11269

C=C(CO)C(C#N)O 1880

C=C[C@@H]1[C@@H]2C[C@@H]3C4=C(CCN3C(=O)C2=C(O[C@H]1O[C@H]5[C@@H]([C@H]([C@@H]([C@H](O5)CO)O)O)O)O)C6=CC=CC=C6N4 9018

C=C1[C@H](C[C@H]2[C@@H]1[C@@H](OC=C2C(=O)O)O[C@H]3[C@@H]([C@H]([C@@H]([C@H](O3)CO)O)O)O)O 3253

C=C1C[C@@H]2[C@]34CO[C@@H]([C@]1([C@H]3C[C@@H](C=C4C(=O)O2)O)C[C@@H](C5=COC=C5)O)O 6919

C=C1CC[C@@H]2[C@H]([C@@H]3[C@H]1CC(C3=C)O)OC(=O)C2=C 9254

C=C1CCC(CC1)C(=C)O 5347

C=CC(C#CC#CC(CCO)O)O 8678

C=CC1=C2CCOC(=O)C2=CN=C1 3286

C=CCC1=C(C(=CC=C1)O)O 322

C=CCC1=CC(=C(C=C1)O)C2=C(C=CC(=C2)/C=C/C=O)O 7312

C=CCC1=CC=C(C=C1)O 1417

C=CCCCCCC(C(CC#CC#CC(C=C)O)O)Cl 3350

C=CCCCCCC/C=C/CCCCCCCC=O 10987

C1(OO1)O 9749

C1[C@@]([C@H](C2=C(O1)C=C(C=C2)O)O)(CC3=CC(=C(C=C3)O)O)O 7651

C1[C@@H]([C@@H]([C@H]([C@@H](O1)CC(=O)O)OC(=O)C2=CC3=CC(=C(C=C3[C@H]([C@@H]2C(=O)O)C4=CC(=C(C=C4)O)O)O)O)O)O 8708

C1[C@@H]([C@@H](OC2=CC(=CC(=C21)O)O)C3=CC(=C(C(=C3)O)O)O)O 9448

C1[C@@H]([C@H]([C@@H]([C@H]([C@@H]1OC2=CC(=CC3=[O+]C(=C(C=C23)O[C@H]4[C@H]([C@@H]([C@H]([C@@H](O4)CO)O)O)O[C@@H]5[C@H]([C@@H]([C@H]([C@@H](O5)CO)O)O)O)C6=CC(=C(C=C6)O)O)O)O)O)O)CO 1876

C1[C@@H]([C@H](OC1=O)COP(=O)(O)OP(=O)(O)OP(=O)(O)O)OP(=O)(O)OP(=O)(O)OP(=O)(O)O 960

C1[C@@H]([C@H](OC2=C1C(=CC(=C2[C@@H]3[C@H]([C@H](OC4=CC(=CC(=C34)O)O)C5=CC=C(C=C5)O)O)O)O)C6=CC(=C(C=C6)O)O)O 3211

C1[C@@H](N(C2=C(N1)NC(=NC2=O)N)C=O)CNC3=CC=C(C=C3)C(=O)N[C@@H](CCC(=O)[O-])C(=O)[O-] 10984

C1[C@@H]2[C@H]([C@@H]([C@H]([C@@H](O2)OC(=O)C3=CC(=C(C(=C3)O)O)O)OC(=O)C4=CC(=C(C(=C4)O)O)O)OC(=O)C5=CC(=C(C(=C5C6=C(C(=C(C=C6C(=O)O1)O)O)O)O)O)O)OC(=O)C7=CC(=C(C(=C7)O)O)O 7210

C1[C@@H]2[C@H]([C@@H]([C@H]([C@H](O2)O)O)O)OC(=O)C3=CC(=C(C(=C3C4=C(C(=C5C6=C4C(=O)OC7=C(C(=C(C8=C(C(=C(C=C8C(=O)O1)O)O)O)C(=C67)C(=O)O5)O)O)O)O)O)O)O 7212

C1[C@@H]2[C@H]([C@@H]([C@H](C(O2)O)OC(=O)C3=CC(=C(C(=C3OC4=C(C(=C5C(=C4)C(=O)OC[C@@H]6[C@H]([C@@H]([C@H](C(O6)O)OC(=O)C7=CC(=C(C(=C7)OC8=C(C(=C9C(=C8)C(=O)OCC2[C@H]([C@@H]([C@H](C(O2)O)OC(=O)C2=CC(=C(C(=C2)O)O)O)OC(=O)C2=CC(=C(C(=C2)O)O)O)OC(=O)C2=CC(=C(C(=C29)O)O)O)O)O)O)O)OC(=O)C2=CC(=C(C(=C2)O)O)O)OC(=O)C2=CC(=C(C(=C25)O)O)O)O)O)O)O)O)OC(=O)C2=CC(=C(C(=C2)O)O)O)OC(=O)C2=CC(=C(C(=C2C2=C(C(=C(C=C2C(=O)O1)O)O)O)O)O)O 10683

C1[C@@H]2[C@H](OC[C@@]2([C@@H](O1)C3=CC4=C(C=C3)OCO4)O)C5=CC6=C(C=C5)OCO6 6586

C1[C@H]([C@@H]([C@H]([C@@H](O1)O[C@@H](CCC2=CC(=C(C=C2)O)O)CC(=O)CCC3=CC(=C(C=C3)O)O)O)O)O 6360

C1[C@H]([C@@H]([C@H]([C@@H](O1)OC[C@@H]2[C@H]([C@@H]([C@H](C(O2)OC3=C(C4=C(C=C3)C(=O)C5=CC=CC=C5C4=O)O)O)O)O)O)O)O 9810

C1[C@H]([C@@H](OC2=CC(=CC(=C21)O)O)C3=CC(=C(C=C3)O)O)O 9319

C1[C@H]([C@H](OC2=C1C(=CC(=C2[C@@H]3[C@H]([C@H](OC4=C(C(=CC(=C34)O)O)[C@@H]5[C@H]([C@H](OC6=CC(=CC(=C56)O)O)C7=CC(=C(C=C7)O)O)O)C8=CC(=C(C=C8)O)O)O)O)O)C9=CC(=C(C=C9)O)O)O 7058

C1[C@H]([C@H](OC2=C3[C@H](CC(=O)OC3=CC(=C21)O)C4=CC(=C(C=C4)O)O)C5=CC(=C(C=C5)O)O)O 1502

C1[C@H]([C@H](OC2=CC(=CC(=C21)O)O)C3=CC(=C(C(=C3)O)O)O)O 2727

C1[C@H](C(OC2=CC(=CC(=C21)O)O)C3=CC(=C(C=C3)O)O)O 2715

C1[C@H](N2C(=C/C(=C\3/C=C4C5=C(C[C@H](N4C3=O)C(=O)O)C6=CC=CC=C6N5)/C2=O)C7=C1C8=CC=CC=C8N7)C(=O)O 8650

C1[C@H](OC2=CC(=CC(=C2C1=O)O)OC3C(C(C(C(O3)CO)O)O)O)C4=CC=C(C=C4)O 7111

C1[C@H]2[C@H](CO[C@@H]2C3=CC4=C(C=C3)O[C@H]([C@@H](O4)C5=CC(=C(C=C5)O)O)CO)[C@H](O1)C6=CC(=C(C=C6)O)O 7043

C1=C(C=C(C(=C1O)O)O)C(=O)O[C@@H]2[C@H](O[C@@H]([C@@H]([C@H]2OC(=O)C3=CC(=C(C(=C3)O)O)O)OC(=O)C4=CC(=C(C(=C4)O)O)O)OC(=O)C5=CC(=C(C(=C5)O)O)O)CO 11901

C1=C(C=C(C(=C1O)O)O)C(=O)O[C@H]2[C@H]([C@H]3[C@@H]([C@H](O2)OC(=O)C4=CC(=C(C(=C4C5=C(C(=C(C=C5C(=O)O3)O)O)O)O)O)O)O)O 9362

C1=C(C=C(C(=C1O)O)O)C(=O)OC[C@H]([C@H]([C@@H]([C@H](C=O)O)O)O)O 3200

C1=C(C=C(C(=C1O)O)O)C2=CC(=O)C3=C(C(=C(C=C3O2)O)[C@H]4C([C@H]([C@@H](C(O4)CO)O)O)O)O 4232

C1=C(C=C(C(=C1O)O)OS(=O)(=O)[O-])C2=C(C(=O)C3=C(C=C(C=C3O2)O)O)OC(=O)C4=CC(=C(C(=C4)O)O)O.[K+] 5912

C1=C(C=C(C=C1O)OC2=C(C=C(C3=C2OC4=C(C=C(C(=C4O3)C5=C6C(=C(C=C5O)O)OC7=C(O6)C(=CC(=C7OC8=CC(=CC(=C8)O)O)O)O)O)O)O)O)O 926

C1=C(C=C(C=C1O)OC2=C(C=C(C3=C2OC4=C(C=C(C(=C4O3)C5=C6C(=C(C=C5O)O)OC7=C(O6)C(=CC(=C7OC8=CC(=CC(=C8)O)O)OC9=C(C=C(C=C9O)O)O)O)O)O)O)O)O 8697

C1=C(C=C(C=C1O)OC2=C(C=C(C3=C2OC4=C(C=C(C=C4O3)O)O)O)O)O 2655

C1=C(C=C2C(=C1O)C(=O)C3=C(C=C(C=C3C2=O)O)O)CO 1552

C1=C(NC(=O)NC1=O)C(=O)[O-].[Na+] 10167

C1=C2[C@H]([C@@H]([C@@H](C2=CO1)O)O)CCO 4497

C1=CC(=C(C(=C1)O)O)CCCCCCC/C=C\CCCC2=C(C(=CC=C2)O)O 3321

C1=CC(=C(C(=C1)O)O)O 7234

C1=CC(=C(C(=C1CCC(=O)O)O)O)O 11950

C1=CC(=C(C=C1/C=C/C2=CC(=CC(=C2)O[C@H]3[C@@H]([C@H]([C@@H]([C@H](O3)CO)O)O)O)O)O)O 734

C1=CC(=C(C=C1/C=C\C2=CC(=CC(=C2)O)O)O)O 10985

C1=CC(=C(C=C1C[C@@H](C(=O)O)OC(=O)/C=C\C2=C(C(=C(C=C2)O)O)/C=C/C3=CC(=C(C=C3)O)O)O)O 9456

C1=CC(=C(C=C1C[C@H](C(=O)O)O)O)O 1964

C1=CC(=C(C=C1C=C2C(=O)C3=C(C=C(C=C3O2)O)O[C@H]4C([C@H]([C@@H](C(O4)CO)O)O)O)O)O 1394

C1=CC(=C(C=C1C=CC2=CC(=CC(=O)O2)O)O)O 6402

C1=CC(=C(C=C1C2=C(C(=O)C3=C(C(=C(C=C3O2)O[C@H]4[C@@H]([C@H]([C@@H]([C@H](O4)CO)O)O)O)O)O)O)O)O 7254

C1=CC(=C(C=C1C2=C(C(=O)C3=C(C=C(C=C3O2)O)O)[O-])O)O 7255

C1=CC(=C(C=C1C2=C(C(=O)C3=C(C=C(C=C3O2)O)O)O)O)O[C@H]4[C@@H]([C@H]([C@@H]([C@H](O4)CO)O)O)O 7261

C1=CC(=C(C=C1C2=C(C(=O)C3=C(C=C(C=C3O2)O)O)O[C@H]4[C@@H]([C@H]([C@@H]([C@H](O4)CO[C@H]5[C@@H]([C@H]([C@@H]([C@H](O5)CO[C@H]6[C@@H]([C@H]([C@@H]([C@H](O6)CO)O)O)O)O)O)O)O)O)O)O)O 5791

C1=CC(=C(C=C1C2=C(C(=O)C3=C(C=C(C=C3O2)O)O)OC4[C@@H]([C@H]([C@@H]([C@H](O4)CO)O)O)O)O)OC5[C@@H]([C@H]([C@@H]([C@H](O5)CO)O)O)O 7257

C1=CC(=C(C=C1C2=C(C(=O)C3=C(C=C(C=C3O2)O)O[C@H]4[C@@H]([C@H]([C@H]([C@H](O4)CO)O)O)O)O[C@@H]5[C@@H]([C@H]([C@H]([C@H](O5)CO)O)O)O)O)O 7258

C1=CC(=C(C=C1C2=C(C(=O)C3=C(C=C(C=C3O2)O[C@H]4[C@@H]([C@H]([C@@H]([C@H](O4)CO)O)O)O)O)O)O)O[C@H]5[C@@H]([C@H]([C@@H]([C@H](O5)CO)O)O)O 7259

C1=CC(=C(C=C1C2=C(C(=O)C3=C(C=C(C=C3O2)O[C@H]4[C@@H]([C@H]([C@@H]([C@H](O4)CO)O)O)O)O)O[C@@H]5[C@@H]([C@@H]([C@@H]([C@@H](O5)O)O)O)O)O)O 7256

C1=CC(=C(C=C1C2=C(C(=O)C3=C(C=C(C=C3O2)O[C@H]4C([C@H]([C@@H](C(O4)CO)O)O)O)O)O)O)O 7266

C1=CC(=C(C=C1C2=CC(=O)C3=C(C=C(C=C3O2)O)O[C@H]4[C@@H]([C@H]([C@@H]([C@H](O4)CO)O)O)O)O)O 3207

C1=CC(=C(C=C1C2=CC(=O)C3=C(C=C(C=C3O2)O[C@H]4[C@@H]([C@H]([C@@H]([C@H](O4)CO)O)O)O)O)O)O 1500

C1=CC(=C(C=C1C2=CC(=O)C3=C(C=C(C=C3O2)OC4C(C(C(C(O4)CO)O)O)O)O)O)O 9417

C1=CC(=C(C=C1C2=CC(=O)C3=C(O2)C(=C(C=C3O)O)[C@H]4[C@@H](C([C@@H](C(O4)CO)O)O)O)O)O 6366

C1=CC(=C(C=C1C2=COC3=CC(=CC(=C3C2=O)O)O)O)O 6374

C1=CC(=C(C=C1C2=COC3=CC(=CC(=C3C2=O)O)O[C@H]4C([C@H]([C@@H](C(O4)CO)O)O)O)O)O 6375

C1=CC(=C(C=C1CCC(=O)NCCCNCCCCNCCCNC(=O)CCC2=CC(=C(C=C2)O)O)O)O 4676

C1=CC(=C(C=C1CCO[C@H]2[C@@H]([C@@H]([C@@H]([C@H](O2)CO)O)O)O)O)O 2466

C1=CC(=C(C=C1CCOC(=O)/C=C/C2=CC(=C(C=C2)O)O)O)O 8504

C1=CC(=C(C=C1CO)O)O[C@H]2[C@@H]([C@H]([C@@H]([C@H](O2)CO)O)O)O 2421

C1=CC(=C(C=C1O)O)[C@@H]2[C@H](C(=O)C3=C(C=C(C=C3O2)O)O)O 2393

C1=CC(=C(C=C1O)O)C2=CC(=O)C3=C(C=C(C=C3O2)O)O 6185

C1=CC(=C(C=C1OC2=C(C=C(C=C2I)C[C@@H](C(=O)[O-])[NH3+])I)I)O 8703

C1=CC(=CC(=C1)O)[C@@H](C#N)O[C@H]2[C@@H]([C@H]([C@@H]([C@H](O2)CO)O)O)O 9274

C1=CC(=CC(=C1)O)CCC2=CC=C(C=C2)OC3=CC(=CC(=C3O)O)CCC4=CC(=CC=C4)O 6675

C1=CC(=CC=C1/C=C/C(=O)C2=C(C(=O)C(=O)C=C2O)OC3C(C(C(C(O3)CO)O)O)O)O 1280

C1=CC(=CC=C1/C=C/C(=O)C2=C(C=C(C=C2)O)O)O[C@H]3[C@@H]([C@H]([C@@H]([C@H](O3)CO)O)O)O 4351

C1=CC(=CC=C1/C=C/C(=O)CC(=O)/C=C/C2=CC=C(C=C2)O)O 954

C1=CC(=CC=C1/C=C/C(=O)OC[C@@H]2[C@H]([C@@H]([C@H]([C@@H](O2)OC3=CC(=C4C(=C3)OC(=C(C4=O)O)C5=CC=C(C=C5)O)O)O)O)O)O 942

C1=CC(=CC=C1/C=C/C(=O)OCC2[C@H](C(C([C@@H](O2)OC3=C([O+]=C4C=C(C=C(C4=C3)O[C@H]5C([C@H]([C@@H](C(O5)COC(=O)CC(=O)O)OC(=O)CC(=O)O)O)O)O)C6=CC=C(C=C6)O)O)O)O)O 5776

C1=CC(=CC=C1/C=C/C2=CC(=CC(=C2)O[C@H]3[C@H]([C@H]([C@@H]([C@@H](O3)CO)O)O)O)O)O 6794

C1=CC(=CC=C1/C=C/C2=CC(=CC3=C2[C@@H]([C@H](O3)C4=CC=C(C=C4)O)C5=CC6=C(C(C(O6)C7=CC=C(C=C7)O)C8=CC(=CC(=C8)O)O)C(=C5)O)O)O 3460

C1=CC(=CC=C1/C=C\C(=O)C2=C(C=C(C=C2)O)O)O 4350

C1=CC(=CC=C1/C=C\C(=O)OC[C@@H]2[C@H]([C@@H]([C@H]([C@@H](O2)OC3=CC(=C4C(=C3)OC(=CC4=O)C5=CC=C(C=C5)O)O)O)O)OC(=O)/C=C/C6=CC=C(C=C6)O)O 1392

C1=CC(=CC=C1[C@@H]2[C@@H](C(=O)C3=C(C=C(C=C3O2)O)O)[C@H]4[C@H](OC5=CC(=CC(=C5C4=O)O)O)C6=CC=C(C=C6)O)O 11173

C1=CC(=CC=C1[C@H](C#N)O[C@H]2[C@@H]([C@H]([C@@H]([C@H](O2)CO)O)O)O)O 8340

C1=CC(=CC=C1C#C/C=C/COC2=CC=C(C=C2)O)O 704

C1=CC(=CC=C1C(=O)CO)O 2418

C1=CC(=CC=C1C=O)O[C@H]2[C@@H]([C@@H]([C@@H]([C@H](O2)CO)O)O)O 3679

C1=CC(=CC=C1C2=C(C(=O)C(=C(C2=O)O)C3=CC=C(C=C3)O)O)O 754

C1=CC(=CC=C1C2=C(C(=O)C3=C(C=C(C=C3O2)O)O)O[C@H]4C(C([C@H]([C@H](O4)CO)O)O)O)O 8670

C1=CC(=CC=C1C2=CC(=O)C3=C(C(=C(C=C3O2)O)[C@H]4C([C@H]([C@@H](C(O4)COC(=O)C5=CC(=C(C(=C5)O)O)O)O)O)O)O)O 3203

C1=CC(=CC=C1C2=CC(=O)C3=C(C(=C(C=C3O2)O[C@H]4[C@@H]([C@H]([C@@H]([C@H](O4)C(=O)O)O)O)O)O)O)O 1003

C1=CC(=CC=C1C2=CC(=O)C3=C(O2)C(=C(C=C3O)O)C4=C(OC5=CC(=CC(=C5C4=O)O)O)C6=CC=C(C=C6)O)O 919

C1=CC(=CC=C1C2=CC(=O)C3=C(O2)C=C(C=C3)O)O 2443

C1=CC(=CC=C1CC2=C(C=C(C3=C2OC(=C(C3=O)O)C4=CC=C(C=C4)O)O)O)O 3908

C1=CC(=CC=C1CCC2=CC(=CC(=C2)O)O)O 2402

C1=CC(=CC=C1CN(CC2=CC=C(C=C2)O)O)O 3255

C1=CC(=O)OC2=CC(=C(C=C21)O)O 197

C1=CC(=S)N(C=C1)[O-].C1=CC(=S)N(C=C1)[O-].[Zn+2] 9276

C1=CC=C(C(=C1)C(=O)O)[O-] 9449

C1=CC=C(C(=C1)C(=O)O)[O-].[Ag+] 8343

C1=CC=C(C(=C1)C2=CC(=O)C3=C(C=C(C=C3O2)O)O)O 8686

C1=CC=C(C=C1)/C=C/C(=O)[O-] 9361

C1=CC=C(C=C1)/C=C/C(=O)N 1510

C1=CC=C(C=C1)C(=O)OC[C@@H](/C=C\2/C=CC(=O)O2)O 5340

C1=CC=C(C=C1)C(=O)OCC2=C(C=CC(=C2)O)O[C@H]3[C@@H]([C@H]([C@@H]([C@H](O3)CO)O)OC(=O)C4=CC=CC=C4)O 882

C1=CC=C(C=C1)C(=O)OCC2C(C(C(C(O2)OC3=CC=CC=C3CO)O)O)O 6993

C1=CC=C(C=C1)C2=CC(=O)C3=C(C(=C(C=C3O2)O)O[C@H]4[C@@H]([C@H]([C@@H]([C@H](O4)C(=O)O)O)O)O)O 807

C1=CC=C(C=C1)C2=CC(=O)C3=C(C(=C(C=C3O2)O[C@H]4C([C@H]([C@@H](C(O4)C(=O)O)O)O)O)O)O 808

C1=CC=C(C=C1)CCC(CC(=O)C2=CC=C(C=C2)O)O 1968

C1=CC=C(C=C1)CCCO 6746

C1=CC=C(C=C1)CCN2C(=CC=C2C=O)CO 3230

C1=CC=C(C=C1)CSSCC2=CC=CC=C2 2299

C1=CC=C(C=C1)N=C=S 6742

C1=CC=C(C=C1)NC2=CC3=CC=CC=C3C=C2 6743

C1=CC=C(C=C1)OC2C(C(C(C(O2)(CO)C=CC3=CC=C(C=C3)O)O)(O)O)O 9387

C1=CC=C2C(=C1)C(=CC(=O)C2=O)[O-] 4827

C1=CC=C2C(=C1)C(=O)C(=O)N2 4226

C1=CC=C2C(=C1)C(=O)C3=CC(=C(C(=C3C2=O)O)CO)O 5006

C1=CC=C2C(=C1)C(=O)C3=CC(=CC(=C3C2=O)O)O 7219

C1=CC=C2C(=C1)C=C(C=C2O)O 11017

C1=CC=C2C(=C1)C3=CC(=C(C=C3N2)O)C=O 5826

C1=CC=C2C(=C1)C3=CNSC3=N2 998

C1=CC=C2C(=C1)N(C(=O)C(O2)O)O 2420

C1=CC2=C(C(=C1)O)C(=CN2)C/C(=N/OS(=O)(=O)[O-])/S[C@H]3[C@@H]([C@H]([C@@H]([C@H](O3)CO)O)O)O 11040

C1=CC2=C(C(=C1)O)C(=O)OC(=C2)C3=CC(=C(C=C3)O)O 8569

C1=CC2=C(C(=C1)O)OC(=O)C=C2O 1970

C1=CC2=C(C(=C1)O[C@H]3[C@@H]([C@H]([C@@H]([C@H](O3)CO)O)O)O)C(=O)C4=C([C@@H]2C5[C@@H]([C@H]([C@@H]([C@H](O5)CO)O)O)O)C=C(C=C4O)C(=O)O 7404

C1=CC2=C(C=C1O)O[C@H]([C@@H](C2=O)O)C3=CC(=C(C(=C3)O)O)O 2403

C1=CC2=C(C=C1O)OC(=C2)C3=C(C=C(C=C3)O)O 2468

C1=CC2=C(C=C1O)OC(=C2)C3=CC(=CC(=C3)O)O 5800

C1=CC2=C(C=CN2)C=C1O 10936

C1=CC2=CC(=CN=C2C(=C1)O)O 10986

C1=CN(C=C(C1=O)O)C[C@H](C(=O)O)N 5742

C1=CNC(=C1)C(=O)O 5743

C1=CNC(=C1)C(=O)O[C@H]2[C@@H]([C@H]([C@@H]([C@H](O2)CO)O)O)O 993

C1=CNC(=C1)C=O 7236

C1=CO[C@H]([C@H]2[C@@]1([C@@H](C=C2CO)O)O[C@@H]3[C@@H]([C@H]([C@@H]([C@H](O3)CO)O)O)O[C@H]4[C@@H]([C@H]([C@@H]([C@H](O4)CO)O)O)O)O[C@H]5[C@@H]([C@H]([C@@H]([C@H](O5)CO)O)O)O 7346

C1=CO[C@H]([C@H]2[C@@H]1[C@@H](C3C2(O3)CO)O[C@H]4[C@@H]([C@H]([C@H]([C@H](O4)CO)O)O)O)O[C@H]5[C@@H]([C@H]([C@@H]([C@H](O5)CO)O)O)O 7344

C1=COC([C@H]2[C@@H]1C(C=C2CO)O)O 757

C1=CSC(=C1)C#CC=O 8563

C1=NC2=C(C(=N1)N)N=CN2C[C@@H]([C@@H](C(=O)O)O)O 4842

C1=NC2=C(N1)C(=NC(=O)N2)[O-].[Na+] 9150

C1C(=CC2=C(O1)C=C(C=C2)O)C3=C(C=C(C=C3)O)O 3623

C1C(=CC2=C(O1)C=C(C=C2)O)C3=CC=C(C=C3)O 3624

C1C(=O)COC2=C(C=CC(=C2)O)C3=CC(=C(C=C31)O)O 7100

C1C(C(C(C(C1OCC=CC2=CC=CC=C2)O)O)O)COC3C(C(C(O3)CO)O)O 11149

C1C(C(C(C=C1C(=O)O)O)O)OC(=O)/C=C/C2=CC(=C(C=C2)O)O 11290

C1C(C(C(O1)OC2C(C(C(OC2OC3=CC=C(C=C3)/C=C/C(=O)C4=C(C=C(C=C4)O)O)CO)O)O)O)(CO)O 4906

C1C(C(OC(=O)C2=CC(=C(C(=C2C3=C(C(=C4C5=C3C(=O)OC6=C(C(=C(C7=C(C(=C(C=C7C(=O)O1)O)O)O)C(=C56)C(=O)O4)O)O)O)O)O)O)O)C8C9C(C1=C(C(=C(C(=C1C(=O)O9)C1=C(C(=C(C=C1C(=O)O8)O)O)O)O)O)O)O)O 7209

C1C(C(OC2=CC(=CC(=C21)O)O)C3=CC=C(C=C3)O)OC4C(C(C(C(O4)CO)O)O)O 11090

C1C(OC2=C1C(=CC(=C2)O)C(=O)/C=C/C3=CC=CC=C3)C4=CC=CC=C4 6508

C1C(OC2=CC(=C(C(=C2C1=O)O)O)O)C3=CC=CC=C3 806

C1C[C@@H]([C@@H](NC1)CC(=O)CN2C=NC3=CC=CC=C3C2=O)O 3038

C1C[C@H]([NH2+]C1)C(=O)[O-] 7063

C1C[C@H](C2[C@@H]([C@@H](CN2C1)O)O)O 8180

C1C=CC(=O)OC1C2C(O2)C3=CC=CC=C3 3503

C1C=CCN[C@@H]1C(=O)O 809

C1C2=C([C@@H](S1)CCCCC(=O)O)NC(=O)N2 944

C1C2=CC(=C(C=C2C3C1(COC4=C3C=CC(=C4)O)O)O)O 996

C1C2C(C3=C(O1)C4=C(C=C3)OCO4)OC5=CC6=C(C=C25)OCO6 2513

C1CC[C@@H]2[C@H](C1)C=C[C@@H]([C@H]2C(=O)N3CCCC3)C4=CC5=C(C=C4)OCO5 1923

C1CC2=C(C(=C(C=C2C3=CC(=C(/C=C\C4=CC(=C(C(=C4)C5=CC1=CC(=C5O)Cl)O)Cl)C(=C3O)Cl)Cl)Cl)O)Cl 851

C1CC2=C(C=C(C=C2)O)OC1C3=CC=C(C=C3)O 2442

C1CCCC/C=C/CCCC(=O)CCCC1 1920

C1CCCCC(=O)CCC/C=C\CCC1 1925

C1CCCCCCC(=O)CCCCCC1 1924

C1CCN(CC1)C(=O)/C=C/C=C/CCCCCC/C=C/C2=CC3=C(C=C2)OCO3 6878

C1CCN(CC1)C(=O)/C=C/CCCCCC/C=C/C2=CC3=C(C=C2)OCO3 6877

C1CCN(CC1)C(=O)/C=C\C=C/C2=CC3=C(C=C2)OCO3 1416

C1CCN[C@@H](C1)[C@@]23C[C@@H](C[C@@H]4[C@H]2NCCC4)[C@H]5CCCCN5C3 6371

C1CCN2[C@@H](C1)C34CC2CC=C3CC(=O)O4 2407

C1CCN2C(C1)C34CC2C=CC3=CC(=O)O4 309

C1CCN2C[C@@]34C[C@H]([C@H]2C1)C[C@H]5[C@H]3N(CCC5)[C@H]6C(CC[C@@H]4N6)[C@H]7CCC[C@@H](N7)C89C[C@@H]([C@H]1[C@H]8NCCC1)[C@H]1CCCCN1C9 6372

C1CCN2CC(NC(=O)C2C1)CC3=CC=CC=C3 8974

C1CCNC1.C1CCNC1 7237

C1CN(CCC1N2C(=O)CCC2=O)C(=O)C3=CC=CC=C3 7238

C1CN2CC(C3CC2C14[C@@H]5C3C(CC(=O)N5C6=CC=CC=C46)O)CCO 7103

C1CNC2CC3=CC=CC=C3C4=C2C1=CC5=C4OCO5 502

C1COC2=CC=CC=C21 10964

C1NC2=C(N1[C@H]3[C@@H]([C@@H]([C@H](O3)COP(=O)(O)OP(=O)(O)O)O)O)N=CN=C2N 176

C1OC2=C(O1)C=C(C=C2)C3=COC4=C(C3=O)C=CC(=C4)O 7121

C1OC2=C(O1)C3=C(C=C2)C(=O)C4=NC=CC5=CC6=C(C3=C54)OCO6 3746

C1SC=CS1 2570

CC#CC1=CC=C(SS1)C#CC#CC=C 8561

CC(/C(=C/CC[C@](C)([C@H]1CC[C@]2([C@H]1[C@@H](CC3C2CCC4[C@@]3(C(CC(C4(C)C)O)O)C)O)C)O[C@H]5[C@@H]([C@H]([C@@H]([C@H](O5)CO[C@H]6[C@@H]([C@H]([C@@H]([C@H](O6)CO)O)O)O)O)O)O)/C)O 3555

CC(/C=C\C=C(/C)\C1CNC(C1CC(=O)O)C(=O)O)C(=O)O 9368

CC([C@H]1[C@@H](C[C@@]2([C@@]1(CC[C@]34[C@H]2CC[C@@H]5[C@]3(C4)CC[C@H](C5(C)C)NC)C)C)O)NC 1927

CC(=C(C)CC(=O)O[C@@H]1C=C[C@@]2([C@H](C1(C)C)CC(=O)O2)CO)C 8440

CC(=C)[C@@H]1CC[C@]2([C@H]1[C@H]3CC[C@@H]4[C@]5(CC[C@@H](C([C@@H]5CC[C@]4([C@@]3(C[C@@H]2O)C)C)(C)C)O)C)C 10889

CC(=C)[C@H]1CC2=C(O1)C=C3C(=C2O)C(=O)C(=CO3)C4=CC=C(C=C4)O 4883

CC(=C)C(CC/C(=C/COC1=C2C=CC(=O)OC2=CC3=C1C=CO3)/C)O 3953

CC(=C)C(CCC(C)(C)O)CC1=C(C=C(C(=C1O)C(=O)/C=C/C2=C(C=C(C=C2)O)O)OC)O 4689

CC(=C)C(CCC(C)(C)O)CC1=C(C=C(C2=C1O[C@@H](CC2=O)C3=C(C=C(C=C3)O)OC)O)O 4713

CC(=C)C(COC1=C2C(=CC3=C1OC=C3)C=CC(=O)O2)O 4329

CC(=C)C1([C@@H]2[C@H]([C@]3([C@@]4(CO4)[C@H]5[C@@H]([C@]3([C@H]1C(=O)O2)O)O5)C)O)O 5339

CC(=C)C1C(O1)C2=C(C=CC3=C2OC(=O)C=C3)OC 6709

CC(=C)C1CC2=CC3=C(C=C2O1)OC[C@@H]4[C@H]3OC5=C4C=CC(=C5O)OC 6686

CC(=C[C@@H]1CO[C@]23C[C@]4(CO2)[C@H]([C@H]3[C@@]1(C)O)CC[C@H]5[C@]4(CC[C@@H]6[C@@]5(CC[C@@H](C6(C)C)O[C@@H]7[C@@H]([C@H]([C@H](CO7)O)O[C@@H]8[C@@H]([C@H]([C@H]([C@H](O8)COS(=O)(=O)O)O)O)O)O[C@H]9[C@H]([C@@H]([C@@H](O9)CO)O)O)C)C)C 802

CC(=C[C@H]1C[C@](C2C3CCC4C(C35CC2(O1)OC5)CCC6[C@@]4(CCC(C6(C)C)[C@]7([C@@H]([C@H]([C@@H]([C@H](O7)CO)O)O)O[C@H]8[C@@H]([C@H]([C@@H](O8)CO)O)O)O[C@H]9[C@@H]([C@H]([C@@H]([C@H](O9)CO)O)O)O)C)(C)O)C 804

CC(=C1C=C(C(=O)O1)CN2CCCC2=O)C 8853

CC(=C1CC[C@@]([C@@H](C1)C(=C)C)(C)C=C)C 10925

CC(=C1CC=C(C1)C(=O)C)C 117

CC(=CC[C@@H]1C[C@]23C[C@@H](C(OC2=C(C(=O)[C@@](C3=O)(C1(C)C)CC=C(C)C)C(=O)C4=CC(=C(C=C4)O)O)(C)C)CC=C(C)C)C 1178

CC(=CC[C@H](CC1=C(C=C(C2=C1O[C@@H](CC2=O)C3=C(C=C(C=C3)O)O)OC)O)C(=C)C)C 4691

CC(=CC[C@H](CC1=C(C=C(C2=C1O[C@@H](CC2=O)C3=C(C=C(C=C3)O)OC)O)O)C(=C)C)C 4833

CC(=CC1C2=C(C3=C(O1)C=C(C=C3)O)OC4=C5C=CC(OC5=CC(=C4C2=O)O)(C)C)C 1914

CC(=CCC(C1=CC(=O)C2=C(C=CC(=C2C1=O)O)O)OC(=O)CC(C)(C)O)C 9484

CC(=CCC/C(=C/C=O)/C)C 1550

CC(=CCC/C(=C/CC/C(=C/CC/C=C(\C)/CC[C@@H]1C(=C)CC[C@@H](C1(C)C)O)/C)/C)C 138

CC(=CCC/C(=C/CC1=C(C(=C(C=C1O)O)C/C=C(\C)/CCC=C(C)C)C(=O)C2=C(C=C(C=C2O)O)O)/C)C 3243

CC(=CCC/C(=C/CC1=C(C=C(C(=C1)/C=C/C(=O)C2=C(C=C(C=C2)O)O)O)O)/C)C 4722

CC(=CCC/C(=C/CC1=C(C=CC(=C1O)C(=O)/C=C/C2=CC=C(C=C2)O)O)/C)C 8688

CC(=CCC/C(=C/CC1=C(C=CC(=C1O)O)[C@@H]2CC(=O)C3=C(C4=C(C=C3O2)OC(C(C4)O)(C)C)O)/C)C 8260

CC(=CCC/C(=C/CC1=C(C2=C(C3=C1OC(C=C3)(C)C)OC(=C(C2=O)O)C4=CC(=C(C=C4)O)O)O)/C)C 2596

CC(=CCC/C(=C/CC1=C(OC2=C(C1=O)C(=CC(=C2CC=C(C)C)O)O)C3=C(C=C(C=C3)O)O)/C)C 650

CC(=CCC/C(=C/COC1=CC2=C(C=C1)C=CC(=O)O2)/C)C 762

CC(=CCC/C(=C\C=O)/C)C 1551

CC(=CCC/C=C(\C)/CCC=C(C)C)C 8259

CC(=CCC[C@@](C)([C@@H]1CCC(=CC1)C(=O)OC)O)C 949

CC(=CCC[C@@](C)(C=C)/C=C/C1=CC=C(C=C1)O)C 801

CC(=CCC[C@](C)(C=C)O)C 4924

CC(=CCC[C@](C)(C1CC[C@@]2([C@H]1[C@@H](C[C@@H]3[C@@]2(C[C@@H]([C@H]4[C@@]3(CC[C@@H](C4(C)C)O)C)O)C)O)C)O)C 7097

CC(=CCC1(C(=O)C(=C(/C(=C(/C2=CC=CC=C2)\O)/C1=O)O)C[C@@H]3[C@H](CC[C@]3(C)O)C(=C)C)CC=C(C)C)C 4076

CC(=CCC1(C(=O)C=C(/C(=C(/C=C/C2=CC=CC=C2)\O)/C1=O)OC)CC=C(C)C)C 8799

CC(=CCC1=C(C(=C(C=C1O)O)CC=C(C)C)C2=CC3=C(O2)C4=C(C=C3)OC(C=C4)(C)C)C 655

CC(=CCC1=C(C(=C2C(=C1)C(=O)C3=C(O2)C=CC=C3O)OC)O)C 3387

CC(=CCC1=C(C(=CC(=C1)C(=O)O)OC)O)C 7060

CC(=CCC1=C(C(=CC(=C1)C2=C(C(=O)C3=C(C(=C(C=C3O2)O)CC=C(C)C)O)O)O)O)C 1022

CC(=CCC1=C(C(=CC(=C1)C2=CC(=O)C3=C(C=C(C=C3O2)O)O)CC(C(=C)C)O)O)C 9223

CC(=CCC1=C(C(=CC(=C1)C2CC(=O)C3=C(O2)C(=C(C=C3O)O)CC=C(C)C)O)O)C 3217

CC(=CCC1=C(C=C(C(=C1)C(=O)/C=C/C2=CC(=C(C=C2)O)O)O)O)C 1007

CC(=CCC1=C(C=C(C(=C1)C2COC3=CC(=CC(=C3C2=O)O)O)O)OC)C 5089

CC(=CCC1=C(C=C(C(=C1O)C(=O)CCC2=CC=C(C=C2)O)OC)O)C 2416

CC(=CCC1=C(C=C(C(=C1OC)C2=COC3=C(C2=O)C=CC(=C3)O)O)OC)C 4904

CC(=CCC1=C(C=C(C=C1O)/C=C/C2=CC=C(C=C2)O)O)C 7031

CC(=CCC1=C(C=C(C=C1O)C2=CC3=C(O2)C=C(C=C3)O)O)C 5793

CC(=CCC1=C(C=C(C2=C1OC(=C(C2=O)O)C3=CC=C(C=C3)O)O)O)C 4385

CC(=CCC1=C(C=C(C2=C1OC3=C(C2=O)C(OC4=C3C=CC(=C4)O)C=C(C)C)O)O)C 1915

CC(=CCC1=C(C=C2C(=C1)C(=O)C=C(O2)C3=CC=C(C=C3)O)OC)C 844

CC(=CCC1=C(C=C2C(=C1O)C(=O)C(=C(O2)C3=CC(=C(C=C3)O)O)O)O)C 3220

CC(=CCC1=C(C=C2C(=C1O)C(=O)C(=C(O2)C3=CC(=C(C=C3)O)OC)O)O)C 3221

CC(=CCC1=C(C=C2C(=C1O)C(=O)C(=CO2)C3=CC(=C(C=C3)OC)O)O)C 3214

CC(=CCC1=C(C=C2C(=C1O)C(=O)C(=CO2)C3=CC=C(C=C3)OC)O)C 3213

CC(=CCC1=C(C=C2C(=C1O)C(=O)C=C(O2)C3=CC=C(C=C3)O)O)C 7023

CC(=CCC1=C(C=C2C(=C1O)C(=O)C3=C(C(=C(C=C3O2)O)OC)CCC(C)(C)O)O)C 3242

CC(=CCC1=C(C=C2C(=C1O)C(=O)C3=C(O2)C=CC(=C3)O)O)C 8695

CC(=CCC1=C(C=C2C(=C1OC)C=C(O2)C3=C(C=C(C=C3)O)O)OC)C 3219

CC(=CCC1=C(C=CC(=C1)/C=C/C(=O)C2=C(C=C(C=C2)O)O)O)C 4878

CC(=CCC1=C(C=CC(=C1)/C=C/C(=O)C2=CC3=C(C=C2O)OC(C3)C(C)(C)O)O)C 833

CC(=CCC1=C(C=CC(=C1)C(=O)C2(C(=O)C3=CC(=C(C=C3O2)O)CC=C(C)C)C4=CC(=C(C=C4/C=C\5/C(=O)C6=C(O5)C(=C(C=C6)O)CC=C(C)C)O)O)O)C 4884

CC(=CCC1=C(C=CC(=C1)C2=C(C(=O)C3=C(C=C(C=C3O2)O)O)O)O)C 4346

CC(=CCC1=C(C=CC(=C1)C2CCC3=C(O2)C=C(C=C3)O)O)C 2471

CC(=CCC1=C(C=CC(=C1O)C(=O)/C=C/C2=CC=CC=C2)O)C 10973

CC(=CCC1=C(C=CC(=C1O)C2CC3=C(C4=C(C=C3)OC(CC4)(C)C)OC2)O)C 3797

CC(=CCC1=C(C=CC2=C1C(=O)C3=C(C4=C(C=C3O2)OC(C=C4)(C)C)O)O)C 2169

CC(=CCC1=C(C=CC2=C1C3=C(N2)C=CC(=C3)C=O)O)C 5721

CC(=CCC1=C(C=CC2=C1O[C@@H]3COC4=CC5=C(C=C4[C@]3(C2=O)O)OCO5)OC)C 8886

CC(=CCC1=C(C=CC2=C1OCC3=C2OC4=C3C=CC(=C4)O)O)C 2839

CC(=CCC1=C(C2=C(C(=C1O)CC(C(=C)C)O)O[C@@H](CC2=O)C3=CC(=C(C=C3)O)O)O)C 2599

CC(=CCC1=C(C2=C(C(=C1O)CC=C(C)C)OC([C@H](C2=O)O)C3=C(C=C(C=C3)O)O)O)C 4710

CC(=CCC1=C(C2=C(C(=C1O)CC=C(C)C)OC(=C(C2=O)O)C3=CC(=C(C=C3)O)OC)O)C 2597

CC(=CCC1=C(C2=C(C(=C1O)CC=C(C)C)OC(=C(C2=O)OC)C3=CC(=C(C=C3)O)O)O)C 1009

CC(=CCC1=C(C2=C(C=C1)C(=O)C3=C(O2)C=CC=C3O)O)C 1157

CC(=CCC1=C(C2=C(C3=C1OC(C=C3)(C)C)OC=C(C2=O)C4=CC(=C(C=C4)O)O)O)C 6977

CC(=CCC1=C(OC2=C3C=CC(OC3=CC(=C2C1=O)O)(C)C)C4=C(C=C(C=C4)O)O)C 5812

CC(=CCC1=C2C(=C(C=C1[O-])O)C(=O)C[C@H](O2)C3=C(C=C(C=C3)O)O)C 4834

CC(=CCC1=C2C(=C(C3=C1OC(C=C3)(C)C)O)C(=O)C4C[C@H]5CC6[C@]4(O2)[C@@](C5=O)(OC6(C)C)C/C=C(\C)/C=O)C 2385

CC(=CCC1=C2C(=C(C3=C1OC(C=C3)(C)C)O)C(=O)CC(O2)C4=C(C=CC=C4O)O)C 11010

CC(=CCC1=C2C(=C(C3=C1OC(C=C3)(C)C)O)C(=O)CC(O2)C4=CC=C(C=C4)O)C 10968

CC(=CCC1=C2C(=C(C3=C1OC4=C(C3=O)C=CC=C4O)O)C=CC(O2)(C)C)C 8627

CC(=CCC1=C2C=CC(OC2=C(C=C1C3=C(C(=O)C4=C(O3)C(=C(C=C4O)O)C(C)(C)C=C)O)O)(C)C)C 1011

CC(=CCC1=CC(=C(C(=C1[C@@H]2[C@H](C(=O)C3=C(O2)C(=C(C=C3O)O)CC=C(C)C)O)CC=C(C)C)O)O)C 6690

CC(=CCC1=CC(=C2C(=C1O)C(=O)C3=CC(=CC(=C3O2)O)O[C@H]4[C@@H]([C@H]([C@@H]([C@H](O4)CO)O)O)O)OC)C 8846

CC(=CCCC(=CCC1=C(C=CC2=C1OC(=C2)C3=CC(=CC(=C3)O)O)O)C)C 5844

CC(=CCCC(C)([C@]1(CCC2([C@@H]1[C@@H](CC3[C@]2(CCC4[C@@]3(CCC(C4(C)C)O)C)C)O)C)O)O)C 11270

CC(=CCCC(C)(C=C)C1=CC=CC=C1C(=O)[O-])C 4926

CC(=CCCC(C)(C1CCC2(C1C(CC3C2(CC(C4C3(CCC(C4(C)C)O)C)O[C@H]5[C@@H]([C@H]([C@@H]([C@H](O5)CO)O)O)O[C@@H]6[C@@H]([C@H]([C@@H](CO6)O)O)O)C)O)C)O)C 6238

CC(=CCCC(C1CC[C@@]2(C1(CCC3=C2CC[C@@H]4C3(CCC(C4(C)C)O)C)C)C)C(=O)OC)C 5685

CC(=CCCC(C1CCC2(C1(CCC3=C2CCC4C3(CCC(C4(C)C)OC(=O)C)C)C)C)C(=O)OC5C(C(C(CO5)O)O)O)C 3099

CC(=CCCC1(C=CC2=C(C=CC(=C2O1)O)[C@@H]3CC(=O)C4=C(C(=C(C=C4O3)O)CC=C(C)C)O)C)C 8261

CC(=CCCC1(C=CC2=C(C=CC(=C2O1)O)C3=C(C(=O)C4=C(C=C(C=C4O3)O)O)O)C)C 6689

CC(=CCCC1(C=CC2=C(C3=C(C=C2O1)OC(CC3=O)C4=C(C=C(C=C4)O)O)O)C)C 4721

CC(=CCCC1(C=CC2=C(O1)C(=O)C3=C(C2=O)C=CC(=C3)O)C)C 7227

CC(=CCCC1(C2CCC(C2)C1(C)O)C)CO 10568

CC(=CCCC1(C2CCC1(C(=O)C2)C)C)C 1193

CC(=CCOC1=C2C(=C(C3=C1OC(=O)C=C3)OC)C=CO2)C 6722

CC(=CCOC1=C2C=CC(=O)OC2=CC3=C1C=CO3)C 4336

CC(=NCC(COC(=O)C(CC1=CC=CC=C1)NC(=O)C2=CC=CC=C2)O)C3=CC=CC=C3 8648

CC(=O)/C=C/[C@@]1([C@](CCCC1(C)C)(C)O[C@H]2[C@@H]([C@@H]([C@H]([C@H](O2)CO)O)O)O)O 7342

CC(=O)[C@@]1(CC[C@]2([C@@]1([C@@H](C[C@H]3[C@]2(CC=C4[C@@]3(CC[C@@H](C4)O)C)O)OC(=O)C)C)O)O 5370

CC(=O)[C@@H]1C[C@@]2([C@H](CC[C@]3([C@@]2(CC[C@@H]3C4=COC(=O)C=C4)O)C)[C@@]5(C1=C[C@H](CC5)O[C@H]6C([C@H]([C@@H](C(O6)CO)O)O)O)C)O 7733

CC(=O)[C@H]1CCC2[C@@]1(CCC3[C@H]2CCC4=CC(=O)CC[C@]34C)C 7059

CC(=O)C(=N)C(=O)[O] 8565

CC(=O)C(O)O 2417

CC(=O)C[C@@]1(C(=O)C=C2[C@@H]3[C@]1(OC4=C3C(=CC(=C4O)O)C(=O)O[C@@H]5[C@@H]6[C@@H]([C@@H](COC(=O)C7=CC(=C(C(=C7C8=C(C(=C(C=C8C(=O)O6)O)O)O)O)O)O)O[C@H]5OC(=O)C9=CC(=C(C(=C9)O)O)O)OC2=O)O)O 6773

CC(=O)C1=C(C=C(C=C1)OC)O 9560

CC(=O)C1=C(C=CC(=C1)OC)O 9667

CC(=O)C1=C(C=CC(=C1CCC2=CC=C(C=C2)O)O[C@H]3[C@@H]([C@H]([C@@H]([C@H](O3)CO)O)O)O)O[C@H]4[C@@H]([C@H]([C@@H]([C@H](O4)CO)O)O)O 8824

CC(=O)C1=CC(=C(C=C1)O)O 2419

CC(=O)C1C(CC2(C1(CC(=O)C3(C2CC=C4C3CC(C(C4(C)C)O)O)C)C)C)O 3780

CC(=O)C1CC[C@]2([C@@]1([C@@H](CC3C2(CC=C4C3(CC[C@@H](C4)O)C)O)O)C)O 4950

CC(=O)C1CC[C@]2(C1([C@@H](CC3C2CC=C4C3(CC[C@@H](C4)O)C)OC(=O)C5=CC=CC=C5)C)O 881

CC(=O)C1CCC2[C@@]1(CCC3C2CC=C4[C@@]3(CC(C(C4)N(C)C)O)C)C 4695

CC(=O)CC(C)(C)N1C=NC2=C1N=CN=C2N 3229

CC(=O)CC1=C2C=C3C(=CC2=C4C(=C1)C5=C(C=[N+]4C)C(=C(C=C5)OC)OC)OCO3 57

CC(=O)CCC1=CC(=C(C=C1)O)OC 9277

CC(=O)NN1C=NC2=C(C1=N)N=CN=C2NC3=CC=CC=C3 55

CC(=O)O[C@@H]1[C@@H]([C@]2(CCC3C([C@@]24[C@@H]1O4)CC[C@H]5[C@@]3(CC[C@@H](C5)OC(=O)CCCCCCC(=O)O)C)C)C6=COC(=O)C=C6 1527

CC(=O)O[C@@H]1[C@@H]2[C@@](CC[C@H]([C@]23COC(=O)[C@]45[C@H]3[C@@H](C[C@H](C4)C(=C)C5=O)O)O)(CO1)C 6361

CC(=O)O[C@@H]1[C@H]2C[C@@H]3C4=C(CCN3C[C@H]2C[C@H]([C@H]1O)OC(=O)C5=CC(=C(C(=C5)OC)OC)OC)C6=C(N4)C=C(C=C6)OC 7143

CC(=O)O[C@@H]1C(=C)[C@@]2(CC[C@@H]3[C@]1(C2)C(=O)[C@H]([C@H]4[C@]35CO[C@@H](C4(C)C)CC5=O)O)O 4831

CC(=O)O[C@@H]1C[C@@H]2[C@@]([C@@H]3[C@@]1(C(=O)[C@@](C[C@H]3O)(C)C=C)O)([C@H]([C@H]([C@H](C2(C)C)O)OC(=O)C)OC(=O)C4=CC=CC=C4)C 6396

CC(=O)O[C@@H]1C[C@@H]2[C@@]([C@@H]3[C@@]1(C(=O)[C@@](C[C@H]3O)(C)C=C)O)([C@H]([C@H]([C@H](C2(C)C)OC(=O)C)OC(=O)C)OC(=O)C4=CC=CC=C4)C 6394

CC(=O)O[C@@H]1C[C@@H]2[C@](C=C(C(=O)C2(C)C)OC(=O)C)([C@@H]3[C@@]1(C(=O)[C@@](C[C@H]3O)(C)C=C)O)C 6398

CC(=O)O[C@@H]1C[C@@H]2C3(CO[C@@H]([C@H]3C1(C)C)O)C4CCC5CC4(C(=O)C5=C)C(=O)O2 2701

CC(=O)O[C@@H]1C[C@H]2[C@@]3(C=C1)[C@H](CN2CC4=CC5=C(C=C34)OCO5)O 115

CC(=O)O[C@@H]1CN(C(=O)C=C1)C(=O)CCC2=CC=CC=C2 6891

CC(=O)O[C@H]1C(C(C23C([C@]1(C)C=O)C4C(C56C2C(C([C@@H](C5C3N4C)OC(=O)C7=CC=CC=C7)C(=C)C6)OC(=O)C)O)OC(=O)C)OC(=O)C8=CC=CC=C8 2142

CC(=O)O[C@H]1C[C@@]2([C@@H]3CC[C@@H]4C[C@H](CC[C@@]4([C@H]3CC[C@@]2([C@H]1C5=CC(=O)OC5)C)C)O)O 111

CC(=O)O[C@H]1C[C@@H]2[C@]3(C=CC(=O)C([C@@H]3C[C@H]([C@]2(C4=CC[C@H]([C@]14C)C5=CC(OC5=O)O)C)O)(C)C)C 8805

CC(=O)O[C@H]1C[C@]2([C@@H]3[C@H](C[C@@H]4C[C@@]3(C[C@@H]([C@@H]2C([C@H]1OC(=O)C)(C)C)OC(=O)C)C(=O)C4=C)O)C 5025

CC(=O)O[C@H]1C=C([C@@H]2[C@H]1C(=CO[C@H]2O[C@H]3[C@@H]([C@H]([C@@H]([C@H](O3)CO)O)O)O)C(=O)O)CO 131

CC(=O)O[C@H]1CC[C@@H]2[C@]3([C@H]1O)CCN2CC4=C(C5=C(C=C34)OCO5)OC 2718

CC(=O)O[C@H]1CC[C@]2(C([C@]1(C)CO)CC[C@@]3(C2[C@@H](C[C@@]4(C3=CC[C@@]5([C@H]4CC(CC5)(C)C)C(=O)O)C)O)C)C 141

CC(=O)O[C@H]1CCC([C@@H]2[C@@]13CO[C@@]([C@H]2OC(=O)C)([C@]45[C@H]3CC[C@H](C4)C(=C)C5=O)O)(C)C 2664

CC(=O)O[C@H]1CCC([C@]2([C@]1([C@H]3CC4=C(C=CO4)[C@@](C3[C@H]([C@@H]2OC(=O)C)OC(=O)C)(C)OC)C)O)(C)C 1123

CC(=O)O[C@H]1CCC([C@]2([C@]1([C@H]3CC4=C(C=CO4)[C@@H]5[C@@H]3[C@H](C2)OC5=O)C)O)(C)C 1120

CC(=O)O[C@H]1CCC([C@]2([C@]1([C@H]3CC4=C(C=CO4)[C@]([C@@H]3[C@H]([C@@H]2OC(=O)C)OC(=O)C)(C)O)C)O)(C)C 1121

CC(=O)OC([C@H]1[C@H]([C@H]2[C@@H](O1)CC(=O)O2)O)C3=CC=CC=C3 113

CC(=O)OC(C)(C)CCC(=O)C(C)(C1C(CC2(C1(CC(=O)C3(C2CC=C4C3CC(=O)C(C4(C)C)O)C)C)C)O)O 2384

CC(=O)OC[C@@]1([C@H](CCC2([C@@H]1C=O)COC(=O)C34C2CC[C@H](C3)C(=C)C4=O)O)C 8646

CC(=O)OC[C@@]12[C@H]([C@H]([C@H]3[C@]([C@@]14[C@@H]([C@@H](C(=O)[C@H]2OC(=O)C)[C@@](O4)(COC(=O)C5=C(CCC(C(=O)O3)(C)O)N=CC=C5)C)OC(=O)C)(C)O)OC(=O)C6=CC=CC=C6)OC(=O)C 9562

CC(=O)OC[C@@H]1[C@H]([C@@H]([C@H]([C@@H](O1)O[C@H]2[C@@H]([C@@H]3CCOC(=O)C3=CO2)C=C)OC(=O)C4=CC=CC=C4)OC(=O)C)OC(=O)C5=C(C(=CC=C5)O[C@H]6[C@@H]([C@H]([C@@H]([C@H](O6)CO)O)O)O)O 10940

CC(=O)OC[C@@H]1[C@H]([C@@H]([C@H]([C@@H](O1)O[C@H]2[C@@H]([C@H](O[C@@H]([C@@H]2O[C@H]3[C@@H]([C@H]([C@@H]([C@H](O3)CO)O)O)O)O[C@]4([C@H]([C@@H]([C@H](O4)CO)O)OC(=O)C5=CC=CC=C5)COC(=O)/C=C/C6=CC(=C(C=C6)O)O)CO)OC(=O)/C=C/C7=CC(=C(C=C7)O)OC)O)O)O 9095

CC(=O)OC[C@@H]1[C@H]([C@@H]([C@H]([C@@H](O1)O[C@H]2[C@@H]([C@H](O[C@@H]([C@@H]2O[C@H]3[C@@H]([C@H]([C@@H]([C@H](O3)CO)O)O)O)O[C@]4([C@H]([C@@H]([C@H](O4)CO)O)OC(=O)C5=CC=CC=C5)COC(=O)/C=C/C6=CC(=C(C=C6)O)O)COC(=O)/C=C/C7=CC(=C(C=C7)O)O)O)O)O)O 9099

CC(=O)OC[C@@H]1[C@H]([C@@H]([C@H]([C@@H](O1)O[C@H]2[C@@H]([C@H](O[C@@H]([C@@H]2O[C@H]3[C@@H]([C@H]([C@@H]([C@H](O3)CO)O)O)O)O[C@]4([C@H]([C@@H]([C@H](O4)CO)O)OC(=O)C5=CC=CC=C5)COC(=O)/C=C/C6=CC(=C(C=C6)O)O)COC(=O)C)OC(=O)/C=C/C7=CC(=C(C=C7)O)OC)O)O)O 9094

CC(=O)OC[C@@H]1[C@H]([C@@H]([C@H]([C@@H](O1)O[C@H]2[C@@H]([C@H](O[C@@H]([C@@H]2O[C@H]3[C@@H]([C@H]([C@@H]([C@H](O3)CO)O)O)O)O[C@]4([C@H]([C@@H]([C@H](O4)CO)O)OC(=O)C5=CC=CC=C5)COC(=O)/C=C/C6=CC(=C(C=C6)O)OC)CO)OC(=O)/C=C/C7=CC=C(C=C7)O)O)O)O 9090

CC(=O)OC[C@@H]1[C@H]([C@@H]([C@H]([C@@H](O1)O[C@H]2[C@@H]([C@H](O[C@@H]([C@@H]2O[C@H]3[C@@H]([C@H]([C@@H]([C@H](O3)CO)O)O)O)O[C@]4([C@H]([C@@H]([C@H](O4)CO)O)OC(=O)C5=CC=CC=C5)COC(=O)/C=C/C6=CC=C(C=C6)O)CO)OC(=O)/C=C/C7=CC=C(C=C7)O)O)O[C@H]8[C@@H]([C@H]([C@@H]([C@H](O8)CO)O)O)O)OC(=O)C 8429

CC(=O)OC[C@@H]1[C@H]([C@@H]([C@H]([C@@H](O1)OC2=C(C=C(C=C2/C=C/C3=CC=C(C=C3)O)O)O)O)O)O 11911

CC(=O)OC[C@@H]1[C@H]([C@@H]([C@H]([C@H](O1)CO[C@]2([C@@H]([C@H]([C@H](O2)COC(=O)/C=C/C3=CC=C(C=C3)O)OC(=O)C)O)COC(=O)C)O)OC(=O)C)OC(=O)C 7112

CC(=O)OC[C@@H]1[C@H]([C@@H]([C@H]([C@H](O1)O[C@]2([C@H]([C@@H]([C@H](O2)CO)O)OC(=O)C3=CC=CC=C3)COC(=O)/C=C/C4=CC=C(C=C4)O)O[C@H]5[C@@H]([C@H]([C@@H]([C@H](O5)CO)O)O)O)O[C@@H]6[C@@H]([C@H]([C@@H]([C@H](O6)CO)O)O[C@@H]7[C@@H]([C@H]([C@@H]([C@H](O7)CO)O)O)O)O)OC(=O)/C=C/C8=CC(=C(C=C8)O)OC 8426

CC(=O)OC[C@@H]1[C@H]([C@@H]([C@H]([C@H](O1)O[C@H]2[C@@H]([C@H](O[C@@H]([C@@H]2O[C@H]3[C@@H]([C@H]([C@@H]([C@H](O3)CO)O)O)O)O[C@]4([C@H]([C@@H]([C@H](O4)CO)O)OC(=O)C5=CC=CC=C5)COC(=O)/C=C/C6=CC=C(C=C6)O)COC(=O)C)OC(=O)/C=C/C7=CC(=C(C=C7)O)OC)O)O[C@@H]8[C@@H]([C@H]([C@@H]([C@H](O8)CO)O)O)O)OC(=O)C 8423

CC(=O)OC[C@@H]1[C@H]([C@@H]([C@H]([C@H](O1)O[C@H]2[C@@H]([C@H](O[C@@H]([C@@H]2O[C@H]3[C@@H]([C@H]([C@@H]([C@H](O3)CO)O)O)O)O[C@]4([C@H]([C@@H]([C@H](O4)CO)O)OC(=O)C5=CC=CC=C5)COC(=O)/C=C/C6=CC=C(C=C6)O)COC(=O)C)OC(=O)C)O)O[C@@H]7[C@@H]([C@H]([C@@H]([C@H](O7)CO)O)O)O)O 8431

CC(=O)OC[C@@H]1C(CC[C@@H](C12COC(=O)C34[C@H]2[C@H](C[C@@H](C3)C(=C)C4=O)O)OC(=O)C)(C)C 7295

CC(=O)OC[C@]1([C@@H](CC[C@@]2([C@H]1C[C@@H]([C@@]34[C@H]2CC[C@@H](C3)C(=C)C4)O)C)O)C 4948

CC(=O)OC[C@]12[C@@H](C(C3C(C1([C@@](CCC2OC(=O)C)(C)O)OC3(C)C)OC(=O)C)OC(=O)C4=CC=CC=C4)OC(=O)C5=CN=CC=C5 11933

CC(=O)OC1=CC=CC(=C1CCCCCCCCCCC(=O)C2=C(C=C(C=C2O)OC)O)O 569

CC(=O)OC1C(=C)C2CC(C3C1(C2)C(=O)OC4C35CO[C@H]([C@@H]5C(CC4)(C)C)O)O 7753

CC(=O)OC1C(C(C2CC(C3(C(C2(C1OC(=O)C4=CC=CC=C4)C)C(=O)CC(C3=O)(C)C=C)O)O)(C)C)OC(=O)C 6385

CC(=O)OC1C(C(C2CC(C3(C(C2(C1OC(=O)C4=CC=CC=C4)C)C(CC(C3=O)(C)C=C)O)O)O)(C)C)OC(=O)C 6384

CC(=O)OC1C(C(C2CC(C3(C(C2(C1OC(=O)C4=CC=CC=C4)C)C(CC(C3=O)(C)C=C)OC(=O)C5=CC=CC=C5)O)O)(C)C)O 6393

CC(=O)OC1C(C(C2CC(C3(C(C2(C1OC(=O)C4=CC=CC=C4)C)C(CC(C3=O)(C)C=C)OC(=O)C5=CC=CC=C5)O)O)(C)C)OC(=O)C 6383

CC(=O)OC1C(C(OC2=C1C3=C(C=C2)C=CC(=O)O3)(C)C)O 7241

CC(=O)OC1C(C2(CCC3C(C24C1O4)CCC5[C@@]3(CCC(C5)OC(=O)CCCCCCC(=O)NC(CCCN=C(N)N)C(=O)O)C)C)C6=COC(=O)C=C6 1530

CC(=O)OC1C(C2=C(C=CC3=C2OC(=O)C=C3)OC1(C)C)O 7240

CC(=O)OC1C2[C@@H]3C(CC4=C(C3C(=O)O2)C=CO4)[C@@]5(C1(C(CC(C5O)O)(C)C)O)C 1119

CC(=O)OC1C2C(C(C3C1(C(CC4C3(CCCC4(C)C)C)O)C(=O)C2=C)O)O 7284

CC(=O)OC1CC2(C3CCC4CC(CCC4(C3CCC2(C1C5=COC(=O)C=C5)C)C)OC(=O)CCC(=O)NC(CCCN=C(N)N)C(=O)O)O 1057

CC(=O)OC1CC2C(C(C(C(C2(C3C1(C(=O)C(C(C3OC(=O)C4=CC=CC=C4)O)(C)C=C)O)C)OC(=O)C5=CC=CC=C5)O)OC(=O)C)(C)C 6392

CC(=O)OC1CC2C(C(C(C(C2(C3C1(C(=O)C(C(C3OC(=O)C4=CC=CC=C4)O)(C)C=C)O)C)OC(=O)C5=CC=CC=C5)OC(=O)C)O)(C)C 6388

CC(=O)OC1CC2C(C(C(C(C2(C3C1(C(=O)C(CC3=O)(C)C=C)O)C)OC(=O)C4=CC=CC=C4)OC(=O)C)O)(C)C 6389

CC(=O)OC1CC2C(C(C(C(C2(C3C1(C(=O)C(CC3=O)(C)C=C)O)C)OC(=O)C4=CC=CC=C4)OC(=O)C)OC(=O)C)(C)C 6386

CC(=O)OC1CC2C(C(C(C(C2(C3C1(C(=O)C(CC3O)(C)C=C)O)C)OC(=O)C4=CC=CC=C4)OC(=O)C)OC(=O)C5=CC=CC=C5)(C)C 6391

CC(=O)OC1CC2C(C(C(C(C2(C3C1(C(=O)C(CC3OC(=O)C4=CC=CC=C4)(C)C=C)O)C)OC(=O)C5=CC=CC=C5)O)O)(C)C 6387

CC(=O)OCC([C@]1(CCC2C(=C1)CCC3C2(C[C@@H](C[C@@]3(C)CO)O)C)C)O 119

CC(=O)OCC(C(=C)C(=O)O[C@H]1C[C@]2([C@@H](CC[C@@H]([C@@H]2[C@H]([C@@H]1C(=C)C(=O)OC)O)CO)O)C)O 756

CC(=O)OCC1(C2CCC3C45CCCC(C4C(C(C3(C2O)C1O)(OC5)O)OC(=O)C)(C)C)O 7291

CC(=O)OCC1C(OC2=C1C=C(C=C2OC)C=CCOC3C(C(C(C(O3)COC(=O)C(=C)CCO)O)O)O)C4=CC(=C(C(=C4)OC)OC)OC 9136

CC(C(=O)C1=NC2C(=NC1)NC(=O)NC2=O)O 9161

CC(C(=O)OC1[C@@H]2CC[C@@H]3C1(C[C@H]([C@]4([C@H]([C@]3(C)O)C5[C@@H](C4(C)C)O5)O)O)C[C@@]2(C)O)O 675

CC(C(CC([C@@](C)([C@H]1CCC2([C@@]1(CCC3C2=CC(=O)[C@H]4[C@@]3(C[C@@H]([C@@H](C4)O)O)C)C)O)O)O)C(=C)C)O 2074

CC(C)([C@H]1CC2=C(C3=CC=CC=C3[N+](=C2O1)C)OC)O 5650

CC(C)(C#N)O[C@H]1[C@@H]([C@H]([C@@H]([C@H](O1)CO)O)O)O 4928

CC(C)(C(CC1=C(C2=C(C3=C(C=C2)OCO3)N=C1OC)OC)O)O 6368

CC(C)(C)C1=C(C(=CC=C1)C(C)(C)C)O 11031

CC(C)(C)NCC(C1=CC(=CC(=C1)O)O)O.OS(=O)(=O)O 11011

CC(C)(C=C)C1=C(C=C(C2=C1O[C@@H](CC2=O)C3=CC=C(C=C3)O)O)O 8834

CC(C)(C=C)C1=C(C2=C(N1)C=C(C=C2)Br)CCNC 2079

CC(C)(C=C)C1=CC2=C(C=C3C(=C2)C=CO3)OC1=O 1404

CC(C)(C1CC2=C(O1)C=CC(=C2O)C(=O)/C=C/C3=CC=C(C=C3)O)O 815

CC(C)/C(=C/C(=O)OC(C)[C@@]1(CC[C@]2([C@@]1([C@@H](CC3[C@]2(CC=C4[C@@]3(CC[C@@H](C4)O)C)O)OC(=O)/C=C/C5=CC=CC=C5)C)O)O)/C 1517

CC(C)[C@@]1(CC[C@]2([C@H]1[C@H](C[C@@](C(=O)C2)(C)O)OC(=O)C3=CC=C(C=C3)O)C)O 4673

CC(C)[C@@]12CC[C@@](C=C1)(OO2)C 669

CC(C)[C@@H]1CC[C@@]([C@H]2[C@H]1[C@H](CC2)C(=O)C)(C)O 6350

CC(C)[C@@H]1CC[C@]2(CCCC(=C)[C@@H]2[C@H]1O)C 4530

CC(C)[C@@H]1CC[C@]2(CCCC(=C)C2C1=O)C 142

CC(C)[C@]12CC[C@](O1)(C3CC[C@](C3[C@@H]2O)(C)O)C 6365

CC(C)[C@H]1C(=O)N([C@@H](C(=O)O[C@H](C(=O)N([C@@H](C(=O)O[C@H](C(=O)N([C@@H](C(=O)O1)CC2=CC=CC=C2)C)C(C)C)CC3=CC=CC=C3)C)C(C)C)CC4=CC=CC=C4)C 853

CC(C)[C@H]1CC[C@]2(CCCC(=O)[C@]2(C1)C)C 8909

CC(C)[C@H]1CCC(=C)C=C1 10967

CC(C)C(=C)CC(C(C)C1CCC2(C1(CCC3C2=CC(=O)C4C3(CC(C(C4)O)O)C)C)O)O 6975

CC(C)C(=C)CC[C@H]([C@H]1[C@@H](C[C@@]2([C@@]1(CCC3=C2CCC4[C@@]3(CC[C@H](C4(C)C)OC(=O)C)C)C)C)O)C(=O)O 9486

CC(C)C(=C)CCC(C1CCC2([C@@]1(CCC3=C2CCC4[C@@]3(CC[C@@H](C4(C)C)O)C)C)C)C(=O)OC 5533

CC(C)C(=O)[C@H]1C(=O)[C@H](C[C@H](C1(C)C)CC=C(C)C)CC=C(C)C 3238

CC(C)C(=O)[O-].[NH4+] 9360

CC(C)C(=O)C1=C(C(=C(C(=C1O)CC=C(C)C)O)CC2=C(C(C(=O)C(=C2O)C(=O)C(C)C)(C)C)O)O 8840

CC(C)C(=O)C1=C(C2=C(C(=C1O)CC=C(C)C)OC(=O)C=C2C3=CC=CC=C3)O 5368

CC(C)C(=O)C12CC(C(C(C1=O)(C(=O)C3=C2OC(C3)C(C)(C)O)CC=C(C)C)(C)C)CC=C(C)C 3237

CC(C)C(=O)CC1=C(C2=C(C(=CC=C2)OC)N(C1=O)C)OC 6367

CC(C)C(=O)CC1=C(C2=C(C3=C(C=C2)OCO3)N=C1OC)OC 6369

CC(C)C(=O)CCC(C)C1CCC2(C1(CCC34C2CCC5C3(C4)CCC(=O)C5(C)C)C)C 11042

CC(C)C(=O)OCC(C)(C1=C(C=C(C=C1)CO)O)O 2444

CC(C)C(C(=O)O)(N)O 10159

CC(C)C(C)CCC(C)C1CCC2([C@@]1(CC[C@]34C2CCC5[C@]3(C4)CC[C@@H](C5(C)C)OC(=O)/C=C\C6=CC(=C(C=C6)O)OC)C)C 5510

CC(C)C(C[C@H]([C@@](C)(C1CC[C@@]2([C@@]1(CC[C@H]3C2=CC(=O)[C@H]4[C@@]3(C[C@@H]([C@@H](C4)O)O)C)C)O)O)O)O 7194

CC(C)C[C@@H](C(=O)N[C@@H](CS)C(=O)N[C@@H](CC1=CC=C(C=C1)O)C(=O)N[C@@H](CS)C(=O)N[C@@H](CCCNC(=N)N)C(=O)N[C@@H](CCCNC(=N)N)C(=O)N[C@@H](CCCNC(=N)N)C(=O)N[C@@H](CC2=CC=CC=C2)C(=O)N[C@@H](CS)C(=O)N[C@@H](C(C)C)C(=O)N[C@@H](CS)C(=O)N[C@@H](C(C)C)C(=O)NCC(=O)N[C@@H](CCCNC(=N)N)C(=O)O)NC(=O)[C@H](CCCNC(=N)N)NC(=O)CNC(=O)CNC(=O)[C@H](CCCNC(=N)N)N 10979

CC(C)C[C@H]1C(=O)O[C@H](C(=O)N[C@H](C(=O)N([C@H](C(=O)N[C@H](C(=O)N1)CC2=CC=CC=C2)CC(C)C)C)C(C)C)CC(C)C 5662

CC(C)C1=C(C=C2C(=C1)CC[C@@H]3[C@@]2(CC[C@@H](C3(C)C)O)C)O 3790

CC(C)C1=C(C=CC2=C1CCC3[C@@]2(CCCC3(C)C)C)O 8617

CC(C)C1=C(C2=C(C(=O)C1=O)C3(CCCC([C@@H]3C[C@H]2O)(C)C)C)O 3854

CC(C)C1=C(C2=C3C(=C1)C(=O)C4C(C3(CC(=O)O2)C)C(C(=O)O4)(C)C)O 7222

CC(C)C1=C[C@H]([C@@](CC1)(C)C=C)C(=C)C 9410

CC(C)C1=C2C=CC3(C(C2(CC1)C)CCC4C3(CC(C5C4(CCC(C5(C)C)O)C)O)C)C 5766

CC(C)C1=CC(=CC2=C1CCC2(C)O)C(=O)C 1928

CC(C)C1=CC(=O)C(O1)(C)C2C(CC3(C2(CC(=O)C4(C3CC=C5C4CCC(C5(C)C)OC(=O)C)C)C)C)O 6798

CC(C)C1=CC=C(C=C1)CO 9726

CC(C)C12[C@@H]([C@]34[C@H](O3)C[C@@H]5C6=C([C@@H](C[C@]5([C@@]47[C@@H](C1O2)O7)C)O)C(=O)OC6)O 8738

CC(C)C1C(C(=O)NC(C(=O)N/C=C\C2=CC=C(O1)C=C2)CC3=CC=CC=C3)NC(=O)C(CC4=CC=CC=C4)N(C)C 7762

CC(C)C1C2C3C4(C(CCC4(C1C(=O)O2)O)CN3C)C 2187

CC(C)C1CCC(=C)[C@H]2C1CC(=C)CC2 11287

CC(C)CC(=O)C1=C2C(=CC(=C1)C(=O)C)C=CC(O2)(C)C 1921

CC(C)CC(=O)O[C@H]1[C@H]2[C@H](C[C@@H]([C@@]2(CO)O)OC(=O)/C=C\C3=CC=C(C=C3)O)C(=CO1)CO 5053

CC(C)CC(=O)O[C@H]1[C@H]2C(=C[C@@H]([C@]23CO3)OC(=O)CC(C)(C)OC(=O)C)C(=CO1)COC(=O)C 137

CC(C)CC(=O)OC1C2C(CC(C2(COC(=O)C=CC3=CC=C(C=C3)O)O)O)C(=CO1)CO 5054

CC(C)CC(=O)OC1CC2C3(CCC(CC3CCC2(C4(C1(C(CC4)(C(C)OC(=O)CC(C)C)O)C)O)O)O)C 2606

CC(C)CC(=O)OCC1=COC(C2C1C[C@@H](C2(C)O)O)O[C@@H]3[C@@H]([C@H]([C@@H]([C@H](O3)CO)O)O)O 8913

CC(C)CC/C=C(\C)/C=O 10609

CC(C)CCC(=O)O[C@H]1C2C(CC([C@@]23CO3)OC(=O)C)C(=CO1)COC(=O)CC(C)C 2412

CC(C)CCCC(=O)C 9632

CC(C)CCN 4243

CC(C)CCN1C(=CC=C1C=O)CO 3231

CC(C)N=C=S 4396

CC(C1=C(C=C2C(=C1OC)C=CC(O2)(C)C)OC)OC 5590

CC(C1=CC=CC=C1)O 5645

CC(C1C(C[C@@]2([C@@]1(CCC34C2CCC5C3(C4)C=CC(=O)C5(C)C)C)C)O)N(C)C 1108

CC(C1C(CC2(C1(CCC34C2C=CC5C3(C4)CCC(C5(C)C)N(C)C)C)C)O)N(C)C 1926

CC(C1CC[C@@]2([C@@]1(CCC34C2CCC5C3(C4)CCCC5(C)C)C)C)N(C)C 1107

CC(CC(=O)O)(CC(=O)OC/C=C/C1=CC(=C(C(=C1C/C=C/C2=CC(=C(C(=C2)OC)O[C@H]3[C@@H]([C@H]([C@@H]([C@H](O3)CO)O)O)O)OC)OC)O[C@H]4[C@@H]([C@H]([C@@H]([C@H](O4)CO)O)O)O)OC)O[C@H]5[C@@H]([C@H]([C@@H]([C@H](O5)CO)O)O)O 8267

CC(CC(=O)OC1=C(C(=C(C(=C1C2=CC=C(C=C2)O)O)OC(=O)CC(C)OC(=O)C)C3=CC=C(C=C3)O)O)O 1859

CC(CC(=O)OC1=C(C(=C(C(=C1O)C2=CC=C(C=C2)O)OC(=O)C)O)C3=CC=C(C=C3)O)OC(=O)C 1864

CC(CC(=O)OC1=C(C(=C(C(=C1OC(=O)C)C2=CC=C(C=C2)O)OC(=O)CC(C)O)OC(=O)CCC3=CC=CC=C3)C4=CC=C(C=C4)O)O 1858

CC(CC/C=C(\C)/CO)C1CC[C@@]2([C@@]1(CC=C3C2=CCC4[C@@]3(CC[C@@H](C4(C)C)O)C)C)C 3226

CC(CC[C@@H](C(C)(CO)O)O)[C@H]1CC[C@@]2([C@@]1(CC=C3C2=CCC4[C@@]3(CCC(=O)C4(C)C)C)C)C 3228

CC(CC[C@H]([C@@](C)(CO)O)O)C1CCC2(C1(CCC3=C2C(=O)CC4C3(CCC(=O)C4(C)C)C)C)C 3224

CC(CCC(=C)[C@H]1CCC2(C1CCC3[C@]2(CCC4[C@@]3(CCCC4(C)C)C)C)C)C(=C)C 5519

CC(CCC(=O)OC)C1CC(=O)C2(C1(CC(=O)C3=C2C(=O)CC4C3(CCC(=O)C4(C)C)C)C)C 5595

CC(CCC(C)(C(C)(C)O)O)C1CCC2C1(C(CC3C2C(CC4C3(CCC(C4)O)C)O)O)C 6620

CC(CCC=C(CO)CO)[C@H]1CC[C@@]2([C@@]1(CC=C3C2=CCC4[C@@]3(CCC(=O)C4(C)C)C)C)C 3225

CC/C=C/C/C=C/C/C=C/CCCCCCCC(=O)[O-].[Ca+2] 9386

CC/C=C\C/C=C/C/C=C\CCCCCCCC=O 10989

CC[C@@](C)(C(=O)O[C@@H]1[C@H]2[C@]3([C@H]([C@@H]([C@H]4[C@@]2(CO3)[C@@H](C[C@@H]5[C@@]4([C@@H](C(=O)C=C5C)O)C)OC1=O)O)O)C)OC(=O)C 7249

CC[C@@]12C=C[C@@]3([C@H](C(CC3([C@@H]1CCC4C2CCC(C4(C)C)N(C)C)C)O)[C@H](C)NC)C 1906

CC[C@@]12C=CC[NH+]3[C@H]1[C@@]4(CC3)[C@@H]([C@@]([C@H]2OC(=O)C)(C(=O)OC)O)N(C5=CC(=C(C=C45)C6(CC7CC(C[NH+](C7)CCC8=C6NC9=CC=CC=C89)(CC)O)C(=O)OC)OC)C 9010

CC[C@@]12CC[C@@]3([C@H](CCC3([C@@H]1CCC4C2CCC(=O)C4(C)C)C)C(C)CCC(C)C(=C)C)C 1908

CC[C@@]12CC[C@@]3(C(CCC3(C1CCC4C2CC[C@@H](C4(C)C)OC(=O)/C=C/C5=CC(=C(C=C5)O)OC)C)C(C)CCC=C(C)C)C 1893

CC[C@@H](C)[C@@H](C(=O)N[C@H]1[C@@H](OC2=CC=C(C=C2)/C=C\NC(=O)[C@@H](NC1=O)CC(C)C)C(C)C)N(C)C 3124

CC[C@@H](C)[C@H]1C(=O)N2CCC[C@H]2C(=O)N[C@H](C(=O)N3CCC[C@H]3C(=O)N[C@H](C(=O)N[C@H](C(=O)N[C@H](C(=O)N4CCC[C@H]4C(=O)N1)CC5=CC=C(C=C5)O)CC(=O)N)CC6=CC=CC=C6)CC(C)C 5726

CC[C@@H](C)C(=O)OC1[C@@]2([C@@H]3C[C@H]([C@@]4([C@@H]([C@@]3(CO1)[C@H]([C@H]([C@H]2OC(=O)C)OC(=O)C)O)C(=O)[C@H]([C@@]5([C@]46[C@H](O6)C[C@H]5C7=COC=C7)C)O)C)O)C 8640

CC[C@@H]1C[C@H]2C[C@@](C3=C(CCN(C2)C1)C4=CC=CC=C4N3)(C5=C(C=C6C(=C5)[C@]78CCN9[C@H]7[C@@](C=CC9)([C@H]([C@@](C8N6C)(C(=O)OC)O)OC(=O)C)CC)OC)C(=O)OC 4345

CC[C@@H]1CN2CC[C@@]3([C@@H]2C[C@@H]1/C(=C\OC)/C(=O)OC)C4=CC=CC=C4NC3=O 1742

CC[C@@H]1CN2CCC3=C4C2CC1[C@H](N4C5=CC=CC=C35)C(=O)OC 11816

CC[C@@H]1CN2CCC3=CC(=C(C=C3[C@@H]2C[C@@H]1C[C@@H]4C5=CC(=C(C=C5CCN4)OC)OC)OC)OC 2695

CC[C@]1(CN2CCC3=C([C@](CC(C2)C1O)(C4=C(C=C5C(=C4)[C@]67CCN8C6C(C=CC8)([C@H]([C@@]([C@@H]7N5C)(C(=O)OC)O)OC(=O)C)CC)OC)C(=O)OC)NC9=CC=CC=C39)O 9011

CC[C@H](/C=C/[C@@H](C)[C@H]1CC[C@@H]2[C@@]1(CC[C@H]3[C@H]2CC(=O)C4=CC(=O)CC[C@]34C)C)C(C)C 2410

CC[C@H](/C=C/[C@@H](C)[C@H]1CC[C@@H]2[C@@]1(CC[C@H]3[C@H]2CC=C4[C@@]3(CC[C@@H](C4)O)C)C)C(C)C 9725

CC[C@H](/C=C/[C@@H](C)[C@H]1CC[C@@H]2[C@@]1(CC[C@H]3C2=CC[C@@H]4[C@@]3(CC[C@@H](C4)O)C)C)C(C)C 907

CC[C@H](C)[C@H]1C(=O)N[C@H](C(=O)N[C@H](C(=O)N[C@H](C(=O)N2CCC[C@H]2C(=O)N[C@H](C(=O)N[C@H](C(=O)N[C@H](C(=O)N1)CC3=CNC4=CC=CC=C43)CC5=CC=CC=C5)CC6=CC=CC=C6)CCS(=O)C)CC(C)C)CCS(=O)C 1910

CC[C@H](C)[C@H]1C(=O)N[C@H](C(=O)NCC(=O)N[C@H](C(=O)NCC(=O)N2CCC[C@H]2C(=O)N[C@H](C(=O)N1)CC(C)C)CC3=CC=C(C=C3)O)CC(C)C 7144

CC[C@H](CC[C@@H](C)[C@H]1CC[C@@H]2[C@@]1(CC[C@H]3C2=CC[C@@H]4[C@@]3(CC[C@@H](C4)O)C)C)C(C)C 7730

CC[C@H]1[C@H](C[C@@H]2[C@@]1(CC[C@H]3[C@H]2CC=C4[C@@]3(CC[C@@H](C4)O[C@H]5C([C@H]([C@@H](C(O5)CO)O[C@H]6[C@@H](C([C@H](C(O6)C)O)O)O)O)O[C@H]7[C@H](C([C@H](C(O7)C)O)O)O)C)C)OC(=C)[C@H](C[C@@H](C)CO[C@H]8C([C@H]([C@@H](C(O8)CO)O)O)O)OC 2542

CC[C@H]1[C@H](C[C@@H]2[C@@]1(CC[C@H]3[C@H]2CC=C4[C@@]3(CC[C@@H](C4)O[C@H]5C([C@H]([C@@H](C(O5)CO)O[C@H]6[C@@H](C([C@H](C(O6)C)O)O)O)O)O[C@H]7[C@H](C([C@H](C(O7)C)O)O)O)C)C)OC(=C)CC[C@@H](C)CO[C@H]8C([C@H]([C@@H](C(O8)CO)O)O)O 7141

CC[C@H]1C[C@H]2C[C@@H]3[C@H]1N(C2)CCC4=C3NC5=C4C=CC(=C5)[C@H]6C[C@@H]\7C([C@@H](CC8=C6NC9=CC=CC=C89)N(C/C7=C/C)C)C(=O)OC 8207

CC=C(C)C(=O)NC(C1=CC=CC=C1)C(C(=O)OC2CC3(C(C4C(C(CC5C4(CO5)OC(=O)C)O)(C(=O)C(C(=C2C)C3(C)C)OC(=O)C)C)OC(=O)C6=CC=CC=C6)O)O 2716

CC=C(C)C(=O)OC1=C2C(=CC(=C1OC)OC)CC(C(CC3=CC(=C(C(=C32)OC)OC)OC)(C)O)C 10701

CC=C(C)C(=O)OC1CC2(CO2)C3C(C4C(C3(C5C1C(=C)C(=O)O5)O)(O4)C)O 2723

CC1([C@@H](CC2=C(O1)C=CC3=C2OC(=O)C=C3)O[C@H]4[C@@H]([C@H]([C@@H]([C@H](O4)CO)O)O)O)C 7008

CC1([C@H](C[C@H]2[C@]3([C@@H]1[C@@H](OC3)O)[C@@H]4CC[C@@H]5C[C@]4(C(=O)C5=C)C(=O)O2)O)C 2700

CC1([C@H](O1)CC2=C(C3=C(C4=C(C=C3)OCO4)N=C2OC)OC)C 7033

CC1([C@H](O1)COC2=C3C(=C(C4=C2OC(=O)C=C4)OC)C=CO3)C 1113

CC1([C@H]2CC(=O)[C@@]3([C@@H]1[C@@H](C(=O)[C@]45[C@H]3CC[C@H](C4)[C@H](C5=O)CO)O)CO2)C 4830

CC1([C@H]2CCC1(C(=O)C2)C)C 1194

CC1(C[C@]23[C@@H]4CC[C@H]1[C@@H]2CC(=O)[C@H]3COC4=O)C 7248

CC1(C[C@H]([C@H]2[C@@H]1[C@@H](OC=C2)O[C@H]3[C@@H]([C@H]([C@@H]([C@H](O3)CO)O)O)O)O[C@H]4[C@@H]([C@H]([C@H]([C@H](O4)CO)O)O)O)O 7345

CC1(C=CC2=C(C(=C(C=C2O1)OC)C(=O)CC(=O)C3=CC4=C(C=C3)OCO4)OC)C 6990

CC1(C=CC2=C(C3=C(C(=C2O1)C(C)(C)C=C)OC(=O)C(=C3)C(C)(C)C=C)O)C 1565

CC1(C=CC2=C(C3=C(C(=C2O1)C(C)(C)C=C)OC4=C(C3=O)C=CC(=C4O)O)O)C 3912

CC1(C=CC2=C(O1)C(=C3C(=C2)C(=O)C4=C(C(=C(C=C4O3)O)C(C)(C)C=C)O)O)C 3316

CC1(C=CC2=C(O1)C=C(C=C2O)C3=CC4=C(O3)C=C(C=C4)O)C 5794

CC1(C=CC2=C(O1)C=C3C(=C2OC)C4=C(C5=C(O4)C=C(C=C5)O)C(=O)O3)C 3218

CC1(C=CC2=C(O1)C=CC(=C2O)C3CC(=O)C4=C(C=C(C=C4O3)O)O)C 7622

CC1(C=CC2=C(O1)C=CC(=C2OC)/C=C/C(=O)C3=CC=C(C=C3)O)C 4879

CC1(C=CC2=C(O1)C=CC3=C2OC=C(C3=O)C4=CC(=C(C=C4OC)OC)OC)C 828

CC1(C=CC2=C(O1)C=CC3=C2OCC(C3)C4=C(C(=C(C=C4)O)OC)O)C 5412

CC1(C=CC2=C3C(=C(C=C2O1)O)C(=O)C=C(O3)C4=CC=C(C=C4)O)C 737

CC1(C=CC2=C3C(=C(C=C2O1)O)C(=O)C4(C(O3)COC5=CC(=C(C=C54)OC)OC)O)C 4040

CC1(C=CC2=C3C(=C(C=C2O1)OC)C=CC(=O)O3)C 5453

CC1(C=CC2=C3C(=CC(=C2O1)C=O)C4=CC=CC=C4N3)C 5885

CC1(C=CC2=CC(=C(C=C2O1)O)C(=O)/C=C/C3=CC=C(C=C3)O)C 846

CC1(C2=C3C(=CC4=C2OCO4)CCN5CCCC5=C3C1=O)O 3625

CC1(C2C3C(C4(C5(CO5)C6C(C4(C2C(=O)O3)O1)O6)C)O)C 538

CC1(C2CC3C1C3(C2)C)C 10982

CC1(C2CCC(=C)C1C2)C 9567

CC1(C2CCC3(C(C2(CCC1OC4C(C(C(CO4)OC5C(C(C(C(O5)CO)O)O)O)O)OC6C(C(C(C(O6)CO)O)O)O)C)CC7C8(C3(CC(C9(C8CC(C(C9)OC(=O)C1=CC=CC=C1)(C)C=O)C)O)C)O7)C)C 772

CC1(C2CCC3=CC(CCC3C2(CCC1O[C@H]4[C@@H]([C@H]([C@@H]([C@H](O4)CO)O)O)O)C)(C)C(CO)O[C@H]5[C@@H]([C@H]([C@@H]([C@H](O5)CO)O)O)O)C 6016

CC1(CC(C2=C(O1)C=CC(=C2)O)O)C 3564

CC1(CC[C@@H]([C@@]2([C@@H]1C[C@H]([C@]34[C@H]2C[C@@H]([C@H]([C@H]3O)C(=C)C4=O)O)O)CO)O)C 7293

CC1(CC[C@@H]([C@]23[C@@H]1[C@@H]([C@]([C@]45[C@H]2CC[C@H]([C@H]4O)C(=C)[C@H]5O)(OC3=O)O)O)O)C 7298

CC1(CC[C@H]2[C@]3([C@@H]1[C@@H](OC3)O)[C@@H]4[C@H](C[C@H]5CC4([C@@H](C5=C)O)C(=O)O2)O)C 2746

CC1(CC[C@H]2[C@]3([C@@H]1[C@@H](OC3)OC)C4[C@H](C[C@H]5CC4(C(=O)C5=C)C(=O)O2)O)C 7294

CC1(CC=CC2(C3C1CC2(CC3)C)C)C 11151

CC1(CCC(C2(C1CC(C34C2C(CC(C3O)C(=C)C4=O)O)O)C)O)C 7283

CC1(CCC(C23C1C(OC2OC(=O)C45C3CCC(C4)C(=C)C5O)OC)O)C 7289

CC1(CCC2=C(O1)C=CC(=C2)/C=C/C(=O)C3=CC4=C(C=C3O)OC(C4)C(C)(C)O)C 834

CC1(CCC2=C(O1)C=CC(=C2)C3CC(=O)C4=CC5=C(C=C4O3)OC(CC5)(C)C)C 2595

CC1(CCC2=C(O1)C=CC(=C2O)C(=O)/C=C/C3=CC=C(C=C3)O)C 2594

CC1(CCC2=C(O1)C3=C(C4=C2O[C@@H](CC4=O)C5=CC(=C(C=C5)O)O)OC(CC3)(C)C)C 2598

CC1(CCCC2(C1CC(C34C2C(=O)C(C(C3O)C(=C)C4=O)O)O)C)CO 7287

CC1(CCCC2(C1CC(C34C2C(C(C(C3O)C(=C)C4=O)O)O)O)C)C 7288

CC1(CCCC2(C1CC(C34C2C(CC(C3O)C(=C)C4=O)O)O)C)CO 7286

CC1(CCCC2(C1CC(C34C2CC(C(C3O)C(=C)C4=O)O)O)C)CO 7285

CC1(CCCC2=C1C=CC3=C2C(=O)C(=O)C4=C3OC=C4CO)C 7113

CC1[C@@H]([C@@H](C([C@@H](O1)O[C@H]2CC[C@@]3([C@H]4CC[C@@]5([C@H](CC[C@@]5([C@@H]4CCC3=C2)O)C6=COC(=O)C=C6)C)C)O)O)O 7076

CC1[C@@H]([C@@H](C([C@@H](O1)OC2=CC(=C3C(=C2)OC(=C(C3=O)O[C@@H]4[C@H](C([C@H](C(O4)C)OC(=O)C)O)O)C5=CC=C(C=C5)O)O)O)O)O 8179

CC1[C@@H]([C@@H](C([C@@H](O1)OCC2[C@H](C(C([C@@H](O2)OC3=C(OC4=CC(=CC(=C4C3=O)O)O)C5=CC(=C(C=C5)O)OC)O[C@H]6[C@H](C([C@H](C(O6)C)O)O)O)O)O)O)O)O 8820

CC1[C@@H]([C@@H]2CC3C4=C(CCN3C(=O)C2=CO1)C5=CC=CC=C5N4)C=O 2744

CC1[C@@H](C([C@@H]([C@@H](O1)O[C@@H]2[C@@H](OC([C@@H](C2O)O)C)OC3=C(OC4=C(C3=O)C(=CC(=C4CC=C(C)C)O[C@H]5C([C@H]([C@@H](C(O5)CO)O)O)O)O)C6=CC=C(C=C6)O)O)O)O 2558

CC1[C@@H](C([C@@H]([C@@H](O1)OC2[C@@H](OC([C@H](C2=O)O)C)C3=C(C=C4C(=C3O)C(=O)C=C(O4)C5=CC(=C(C=C5)OC)O)O)O)O)O 1312

CC1[C@@H](C([C@@H]([C@@H](O1)OC2[C@H]([C@@H](C(O[C@H]2O[C@H]3CC[C@@]4([C@H]5CC[C@]6([C@H]([C@@H]5CC=C4C3)CC[C@@H]6C(=O)C)C)C)CO)O)O)O)O)O 8059

CC1[C@@H](C([C@@H]([C@@H](O1)OCC2[C@@H]([C@@H](C([C@@H](O2)OC3=C(OC4=CC(=CC(=C4C3=O)O)O)C5=CC(=C(C=C5)O)O)O[C@H]6C([C@H]([C@@H](C(O6)CO)O)O)O)O)O)O)O)O 6683

CC1[C@H](C(C([C@@H](O1)O[C@H]2CC[C@@]3(C4CC[C@@]5([C@H](CC[C@@]5(C4CC[C@@]3(C2)O)O)C6=CC(=O)OC6)C)C=O)O)O)O 2081

CC1[C@H](C(C[C@@H](O1)O[C@@H]2CC[C@@]3(C4CC[C@@]5([C@H](CC[C@@]5(C4CC[C@@]3(C2)O)O)C6=CC(=O)OC6)C)C)OC)O 6666

CC1[C@H](C2=CC(=C(C(=C2C1=O)C)CCO[C@H]3[C@@H]([C@H]([C@@H]([C@H](O3)CO)O)O)O)C)O 7175

CC1=C([C@@H]([C@H]2[C@@H](C1)OC(=O)C2=C)O)[C@@H](C)CCCO 10975

CC1=C([C@H](C[C@H]1C2=COC=C2)OC(=O)C)[C@]3([C@@H]([C@]4(C(CC([C@@]5(C4C(C3OC(=O)C(C)C)OC5)C)OC(=O)C)OC(=O)C)C)COC(=O)C)C 6123

CC1=C(C([C@@H](CC1)O[C@H]2[C@@H]([C@H]([C@@H]([C@H](O2)CO)O)O)O)(C)C)CCC(=O)C 4104

CC1=C(C(=C(C(=C1CCC2=C(C(=C(C(=C2C)C)O)C)C)C)C)O)C 5536

CC1=C(C(=C(C(=C1O)C(=O)C)O)CC2=C(C3=C(C(=C2O)C(=O)/C=C/C4=CC(=C(C=C4)O)O)OC(C=C3)(C)C)O)O 2472

CC1=C(C(=C(C(=C1O)OC)OC)O)CCC(C)CCCC(C)CCCC(C)CCCC(C)C=C.CC(C)CCCC(C)CCCC(C)C 11979

CC1=C(C(=C2C(=C1)[C@@H](C(C2=O)(C)C)O)C)CCO[C@H]3[C@@H]([C@H]([C@@H]([C@H](O3)CO)O)O)O 7176

CC1=C(C(=C2C(=C1)C(=O)C3=CC(=C(C(=C3C2=O)O)OC)O[C@H]4[C@@H]([C@H]([C@@H]([C@H](O4)CO)O)O)O)OC)O 11259

CC1=C(C(=C2C(=C1)C(=O)C3=CC(=CC(=C3C2=O)O)O)OC)O 3955

CC1=C(C(=C2C(=C1)C[C@@](C2=O)(C)CO)C)CCO[C@H]3[C@@H]([C@H]([C@@H]([C@H](O3)CO)O)O)O 7173

CC1=C(C(=C2C(=C1)CC(C2=O)(C)C)C)CCO[C@H]3[C@@H]([C@H]([C@@H]([C@H](O3)CO)O)O)O 7177

CC1=C(C(=C2C(=C1)CC(C2=O)C3C4=C(CCO3)C(=C5C(=C4)CCC5=O)C)C)CCO 5775

CC1=C(C(=C2C(=C1)CC(C2=O)CC3=C(C=C4C(=C3)CCC4=O)CCO)C)CCO 5774

CC1=C(C(=O)C[C@@H]1OC(=O)[C@@H]2[C@H](C2(C)C)/C=C(/C)\C(=O)OC)C/C=C/C=C 7229

CC1=C(C(=O)C[C@]2([C@@H]1C[C@@H]3[C@]45[C@@H]2[C@H]([C@@H]([C@]([C@@H]4[C@H](C(=O)O3)OC(=O)C6=CC=CC=C6)(OC5)C(=O)OC)O)O)C)O 1024

CC1=C(C(=O)C[C@]2([C@H]1C[C@@H]3[C@]45[C@@H]2[C@H]([C@@H]([C@@]([C@@H]4[C@H](C(=O)O3)OC(=O)C=C(C)C)(OC5)C(=O)OC)O)O)C)O 1042

CC1=C(C(=O)C[C@]2([C@H]1C[C@@H]3[C@]45[C@@H]2[C@H]([C@@H]([C@]([C@@H]4[C@H](C(=O)O3)OC(=O)/C=C(/C)\C(C)(C)O)(OC5)C(=O)OC)O)O)C)O 1030

CC1=C(C(=O)C[C@]2([C@H]1C[C@@H]3[C@]45[C@@H]2[C@H]([C@@H]([C@]([C@@H]4[C@H](C(=O)O3)OC(=O)CC(C)C)(OC5)C(=O)OC)O)O)C)O 1028

CC1=C(C(=O)C[C@]2(C1CC3[C@]45C2C([C@@H]([C@@](C4[C@H](C(=O)O3)OC(=O)C)(OC5)C(=O)OC)O)O)C)O 1029

CC1=C(C(=O)C[C@H]1OC(=O)[C@@H]2[C@H](C2(C)C)C=C(C)C)C/C=C/C=C 7228

CC1=C(C(=O)C=CO1)O[C@H]2[C@@H]([C@H]([C@@H]([C@H](O2)CO)O)O)O 2292

CC1=C(C(=O)C=CO1)O[C@H]2[C@@H]([C@H]([C@@H]([C@H](O2)CO)O)O)OC(C)(C)C(COC3=C4C=CC(=O)OC4=CC5=C3C=CO5)O 6466

CC1=C(C(=O)C2=C(C1=O)C=CC=C2O)C3=C(C(=O)C4=C(C3=O)C(=CC=C4)O)C 946

CC1=C(C(=O)N(N1C)C2=CC=CC=C2)N(C)CS(=O)(=O)[O-].[Na+] 10041

CC1=C(C(=O)O[C@H](C1)[C@@](C)([C@H]2CC[C@@H]3[C@@]2(CC[C@H]4[C@H]3C[C@@H]5C6([C@@]4(C(=O)C=C[C@@H]6O)C)O5)C)O)C 9293

CC1=C(C(=O)OC(C1)C2(C3CCC4(C3(CCC5C4CC=C6[C@@]5(C(=O)C=CC6)C)C(=O)O2)O)C)C 9128

CC1=C(C(C[C@@H](C1)O[C@H]2[C@@H]([C@H]([C@@H]([C@H](O2)CO)O)O)O)(C)C)CC[C@@H](C)O 4930

CC1=C(C(CC(C1)O[C@H]2[C@@H]([C@H]([C@@H]([C@H](O2)CO)O)O)O)(C)C)C=C(/C=C(/C)\C=C\C(=C\C=C/C=C(\C)/C=C/C(=C\C(=C\C3=C(CC(CC3(C)C)O[C@@H]4[C@H]([C@@H]([C@@H]([C@@H](O4)CO)O)O)O)C)\O[C@@H]5[C@H]([C@@H]([C@H]([C@@H](O5)CO[C@H]6[C@H]([C@@H]([C@H]([C@@H](O6)CO)O)O)O)O)O)O)/C)\C)O[C@H]7[C@@H]([C@H]([C@@H]([C@H](O7)CO[C@H]8[C@@H]([C@H]([C@@H]([C@H](O8)CO)O)O)O)O)O)O 7091

CC1=C(C(CCC1O[C@H]2[C@@H]([C@H]([C@@H]([C@H](O2)CO)O)O)O)(C)C)C(=O)O 7347

CC1=C(C=C(C(=C1O)C(=O)C(C)C)OC)OC 805

CC1=C(C=C(C(=C1OC)CC=C(C)C)CCC2=C(C(=CC=C2)O)O)O 7034

CC1=C(C=C2C(=C1O)C(=O)C(=CO2)CC3=CC4=C(C=C3)OCO4)O 6349

CC1=C(C=C2C(=C1O)C(=O)C(CO2)CC3=CC4=C(C=C3)OCO4)O 6342

CC1=C(C=C2C(=C1O)C(=O)C3(C(O2)COC4=C3C=CC=C4O)O)OC 970

CC1=C(C=C2C(=C1O)C(=O)C3=C(O2)C(=O)OC4=C3C=CC(=C4)O)O 973

CC1=C(C=C2C(=C1O)C(=O)C3=C(O2)C(OC4=CC=CC=C43)O)O 969

CC1=C(C=C2C(=C1O)C(=O)C3=C(O2)C(OC4=CC=CC=C43)OC)O 968

CC1=C(C=CC(=C1C(=O)C2=C(C=CC(=C2O)C(=O)C)O)O)O 813

CC1=C(C=CC2=C1CCC3=C2C(=CC(=C3C)OC)C(C)OC/C=C(\C)/CCCC(C)CCCC(C)CCCC(C)C)O 6793

CC1=C(C2=C(C(=C1O)C)O[C@@H](CC2=O)C3=CC=CC=C3)O 2163

CC1=C(C2=C(C(=C1O)C=O)OCC(C2=O)CC3=CC4=C(C=C3)OCO4)O 6343

CC1=C(C2=C(C(=C1O)OC)OCC(C2=O)CC3=C(C=C(C=C3)OC)O)O 6344

CC1=C(C2=C(C(=C1OC)C)OC(=CC2=O)C3=CC(=C(C=C3)O)O)O 2519

CC1=C(C2=NC1=CC3=C(C(=C([N-]3)C=C4C(=C(C(=N4)C=C5C(=C(C(=C2)[N-]5)C=C)C)C)CCC(=O)O)CCC(=O)O)C)C.[Fe] 7099

CC1=C(CC2(CC1)C(=C)C[C@@H]([C@@H](C2(C)C)Br)O)Cl 2673

CC1=C(OC=C1)CC(C(C)(C)O)O 2689

CC1=C(OC2=C1C=C(C=C2)CC(=O)C)C3=CC4=C(C=C3)OCO4 5540

CC1=C[C@H]([C@@H]2[C@H]1[C@@H]3[C@@H]([C@@H](C[C@@]2(CO)O)OC(=O)/C(=C/CO)/C)C(=C)C(=O)O3)O 2957

CC1=C[C@H](C(CC1)C(C)C)C2=C(C(=CC(=C2)CC=C)C3=CC(=CC(=C3O)C4C=C(CC[C@H]4C(C)C)C)CC=C)O 2559

CC1=C[C@H]2[C@@H](CC[C@]([C@@H]2CC1)(C)O)C(C)C 1118

CC1=C2[C@@]3(C[C@@H]1O)C(C4[C@]([C@]2(C(=O)OC3(C)C)O)([C@H](C[C@@H]5[C@]4(CO5)OC(=O)C)O)C)OC(=O)C6=CC=CC=C6 9085

CC1=C2[C@@H]([C@@H]3[C@@H]([C@H](C1)O)C(=C)C(=O)O3)C(=CC2=O)COC(=O)CC4=CC=C(C=C4)O 4754

CC1=C2[C@H](/C(=C(/[C@H](C[C@@H](C(=C[C@@H]([C@@H](C2(C)C)C[C@@H]1OC(=O)C)O)COC(=O)C)O)OC(=O)C)\C)/OC(=O)C)OC(=O)C 2034

CC1=C2[C@H]([C@@H]([C@@]3([C@H](C[C@@H]([C@]4([C@H]3[C@@H]([C@@](C2(C)C)(C[C@@H]1OC(=O)C)O)OC(=O)C)CO4)O)OC(=O)C)C)OC(=O)C)OC(=O)C 8392

CC1=C2[C@H]([C@@H]([C@@]3([C@H](C[C@@H]([C@]4([C@H]3[C@@H]([C@@](C2(C)C)(CC1OC(=O)C)O)O)CO4)OC(=O)C)O)C)O)OC(=O)C 4050

CC1=C2[C@H]([C@@H]([C@@]3([C@H](C[C@@H]([C@H]([C@H]3[C@@H]([C@@]2(C[C@@H]1O)C(C)(C)O)O)COC(=O)C)OC(=O)C)OC(=O)C)C)OC(=O)C)OC(=O)C 8310

CC1=C2[C@H]([C@@H]([C@@]3([C@H](C[C@@H]([C@H]([C@H]3[C@@H]([C@@H](C2(C)C)C[C@@H]1OC(=O)C)OC(=O)C)CO)OC(=O)C)O)C)O)OC(=O)C 8297

CC1=C2[C@H]([C@@H]([C@@]3([C@H](C[C@@H](C(=C)[C@H]3[C@@H]([C@@](C2(C)C)(C[C@@H]1OC(=O)C)O)OC(=O)C)O)OC(=O)C)C)OC(=O)C)OC(=O)C 2068

CC1=C2[C@H]([C@@H]([C@@]3([C@H](C[C@@H](C(=C)[C@H]3C[C@@](C2(C)C)(C[C@@H]1OC(=O)C)O)OC(=O)C)O)C)O)OC(=O)C 8301

CC1=C2[C@H]([C@@H]([C@@]3([C@H](C[C@@H](C(=C)[C@H]3C[C@@]2(C[C@@H]1O)C(C)(C)O)OC(=O)/C=C/C4=CC=CC=C4)OC(=O)C)C)OC(=O)C)OC(=O)C5=CC=CC=C5 8318

CC1=C2[C@H]([C@@H]([C@@]3([C@H](C[C@@H](C(=C)C3C[C@@]2(C[C@@H]1O)C(C)(C)O)O)OC(=O)C)C)OC(=O)C)OC(=O)C4=CC=CC=C4 1002

CC1=C2[C@H]([C@@H]([C@@]3([C@H](C[C@@H](C4([C@H]3[C@@H]([C@@H](C2(C)C)C[C@@H]1OC(=O)C)OC(=O)C)CO4)OC(=O)C)OC(=O)C)C)OC(=O)C)OC(=O)C 795

CC1=C2[C@H]([C@@H]([C@@]3([C@H](C[C@@H]4[C@](C3[C@@H]([C@@]2(C[C@@H]1O)C(C)(C)O)OC(=O)C5=CC=CC=C5)(CO4)OC(=O)C)O)C)OC(=O)C)O 8368

CC1=C2[C@H]([C@@H]([C@@]3([C@H](C[C@@H]4[C@](C3[C@@H]([C@@]2(C[C@@H]1O)C(C)(C)O)OC(=O)C5=CC=CC=C5)(CO4)OC(=O)C)OC(=O)C)C)OC(=O)C)O 8307

CC1=C2[C@H]([C@@H]([C@@]3([C@H](C[C@@H]4[C@](C3[C@@H]([C@@]2(C[C@@H]1OC(=O)C)C(C)(C)O)OC(=O)C5=CC=CC=C5)(CO4)OC(=O)C)OC(=O)C)C)OC(=O)C)OC(=O)C6=CC=CC=C6 8305

CC1=C2[C@H]([C@@H]([C@@]3([C@H](CC4[C@](C3C([C@@](C2(C)C)(CC1=O)O)OC(=O)C5=CC=CC=C5)(CO4)OC(=O)C)O)C)O)O 8366

CC1=C2[C@H]([C@@H]([C@@]3([C@H](CC4[C@](C3C([C@@](C2(C)C)(CC1OC(=O)C)O)OC(=O)C5=CC=CC=C5)(CO4)OC(=O)C)O)C)O)O 2012

CC1=C2[C@H]([C@@H]([C@@]3(CC[C@@H](C(=C)[C@H]3[C@@H]([C@@](C2(C)C)(CC1=O)O)OC(=O)C)OC(=O)/C=C/C4=CC=CC=C4)C)OC(=O)C)OC(=O)C 1520

CC1=C2[C@H]([C@@H]([C@@]3(CC[C@@H](C(=C)[C@H]3[C@@H](C(C2(C)C)C[C@@H]1OC(=O)C)O)O)C)OC(=O)C)OC(=O)C 2021

CC1=C2[C@H]([C@@H]([C@]3([C@H](C[C@H](C2(C)C)C[C@@H]1OC(=O)C)C(=C)[C@H](C[C@@H]3OC(=O)C)OC(=O)/C=C/C4=CC=CC=C4)C)OC(=O)C)OC(=O)C 2006

CC1=C2[C@H]([C@H]([C@@]3([C@H](C[C@@H]4[C@]([C@H]3[C@@H]([C@@](C2(C)C)(C[C@@H]1OC(=O)C)O)OC(=O)C)(CO4)OC(=O)C)OC(=O)C)C)OC(=O)C)OC(=O)C 797

CC1=C2[C@H](C([C@@]3([C@H](C[C@@H]4[C@](C3C(C2(CC1O)C(C)(C)O)OC(=O)C)(CO4)OC(=O)C)OC(=O)C5=CC=CC=C5)C)O)O 8304

CC1=C2[C@H](C(=O)[C@@]3([C@@H](C[C@@H]4[C@](C3[C@@H]([C@@](C2(C)C)(C[C@@H]1O)O)OC(=O)C5=CC=CC=C5)(CO4)OC(=O)C)O)C)OC(=O)C 796

CC1=C2[C@H](C(=O)[C@@]3([C@H](C[C@@H]4[C@]([C@H]3[C@@H]([C@@](C2(C)C)(C[C@@H]1OC(=O)[C@@H]([C@H](C5=CC=CC=C5)N(C)C(=O)C6=CC=CC=C6)O)O)OC(=O)C7=CC=CC=C7)(CO4)OC(=O)C)O)C)OC(=O)C 5635

CC1=C2[C@H](C(=O)[C@]3(C/C(=C\[C@@H](C(C2(C)C)C[C@@H]1OC(=O)C)OC(=O)C)/[C@H](C[C@@H]3O)OC(=O)/C=C/C4=CC=CC=C4)C)O 8370

CC1=C2C(=C(C=C1)O)C(=C(C(=O)C2=O)C)O 602

CC1=C2C(=C3C(=C1)C4=CC(=C(C(=C4N3)C5=C6C(=CC(=C5O)OC)C7=CC(=C8C(=C7N6)C=CC(O8)(C)C)C)O)OC)C=CC(O2)(C)C 958

CC1=C2C(=C3C(=C1)C4=CC(=C5C(=C4N3)[C@H]([C@@H](O5)C6=CC(=C(C(=C6)OC)O)OC)CO)OC)C=CC(O2)(C)C 5887

CC1=C2C(=C3C(=C1)C4=CC=CC=C4N3)C5CC(O2)(CCC5C(=C)C)C 1913

CC1=C2C(=CC=C1)OC(=O)C=C2O[C@H]3[C@@H]([C@H]([C@@H]([C@H](O3)CO[C@H]4[C@@H]([C@H]([C@@H]([C@H](O4)CO)O)O)O)O)O)O 5509

CC1=C2C(=O)O[C@@H]([C@@]2(CCC1)C)C3=COC=C3 3131

CC1=C2C(C3C(C(C1)O)C(=C)C(=O)O3)C(=CC2=O)CO 4753

CC1=C2C[C@@H]3[C@]([C@@H]2[C@@H]4[C@@H]([C@H](C1)OC(=O)C)C(=C)C(=O)O4)(O3)C 637

CC1=C2C[C@@H]3C(=C)CCC[C@]3(C[C@H]2OC1=O)C 745

CC1=C2CC[C@@]([C@@H]2C[C@H]3[C@@H](C1)OC(=O)C3=C)(C)O 7128

CC1=C2CC[C@@H]3C([C@H]2CCC1=O)C(=O)C[C@]4(C3(CC[C@@H]4[C@H](C)CC/C=C(\C)/C(=O)O)C)C 11878

CC1=C2CC3C(=C)CC=C[C@@]3(C=C2OC1=O)C 2113

CC1=C2CCC(=O)O[C@]2([C@H]3[C@@H](CC1)C(=C)C(=O)O3)C 7154

CC1=C2CCOC(=O)C2=CC3=C1C(=O)CC3 5825

CC1=CC([C@H](C(C1)OC2=C(C=C(C=C2)O)O)C(=O)C3=C(C=C(C=C3)O)O)C4=C(C=CC5=C4OC(=C(C5=O)CCC(C)(C)O)C6=C(C=C(C=C6)O)O)O 5790

CC1=CC(=C(C(=C1)C)C=O)C 8722

CC1=CC(=C(C(=C1)CC(C)C)O)CC(C)C 953

CC1=CC(=C(C(=C1C(=O)OC2=C(C(=C(C(=C2)C)O)C(=O)OC)C)O)C=O)O 752

CC1=CC(=C(C=C1)C(C)C)OC 4397

CC1=CC(=C(C=C1)OC)OC2=CC=C(C=C2)C[C@H]3C4=C(CCN3C)C=CC5=C4OC6=C(O5)C=C7CCN(C(=O)C7=C6)C 5522

CC1=CC(=C(C2=C1C(=O)C=C(O2)CC(=O)C)[C@H]3[C@@H]([C@H]([C@@H]([C@H](O3)CO)O)O)O)OC 5467

CC1=CC(=C2C(=C1)C(=O)C=C(C2=O)C3=C4C(=O)C=CC(=O)C4=C(C=C3C)O)O 6017

CC1=CC(=C2C(=C1)C(=O)C3=CC(=CC(=C3C2=O)O)O)O[C@H]4[C@@H]([C@H]([C@@H]([C@H](O4)CO)O)O)O 510

CC1=CC(=C2C(=C1)C(=O)C3=CC(=CC(=C3C2=O)O)O[C@H]4[C@@H]([C@](CO4)(CO)O)O)O 3126

CC1=CC(=C2C(=C1)C(=O)C3=CC(=CC(=C3C2=O)OC)O)[O-] 7270

CC1=CC(=C2C(=C1)C3=CC=CC=C3N2)OC 5886

CC1=CC(=C2C(=C1)CC3=CC(=CC(=C3C2=O)O)OC)O 6783

CC1=CC(=C2C(=C1O)C(=O)C3=C(C2=O)C(=CC=C3)O)O 4227

CC1=CC(=C2C3=C1C4=C5C(=C(C=C4CO)O)C(=O)C6=C(C=C(C7=C6C5=C3C8=C7C(=CC(=C8C2=O)O)O)O)O)O 7127

CC1=CC(=CC(=C1)O)O.O 6359

CC1=CC(=CC(=C1C(=O)OC2=CC(=C(C(=C2)C)C(=O)O)O)O)O 4835

CC1=CC(=CC2=C1C=CC(=O)O2)NC(=O)[C@H](CCCN=C(N)N)NC(=O)[C@H](CO)NC(=O)[C@H](CC3=CC=CC=C3)NC(=O)OC(C)(C)C.CC(=O)O 9445

CC1=CC(=O)[C@H]([C@]2([C@H]1C[C@@H]3[C@]45[C@@H]2[C@H]([C@@H]([C@]([C@@]4([C@H](C(=O)O3)O)O)(OC5)CO)O)O)C)O 1035

CC1=CC(=O)[C@H](CC1)C(=C)C 4390

CC1=CC(=O)C([C@]2([C@H]1C[C@@H]3C45[C@@H]2C([C@@H](C(C4(C(C(=O)O3)O)O)(OC5)C)O)O)C)O 1031

CC1=CC(=O)C[C@]2([C@H]1[C@H]([C@@H]3[C@]45[C@@H]2[C@H]([C@@H](C([C@H]4[C@H](C(=O)O3)O)(OC5)C)O)O)O)C 1034

CC1=CC(=O)C2=C(C=C3C(=C2O1)C=CC(O3)(C)C)OC 5464

CC1=CC(=O)C2=C(C3=C(C=C2O1)OC(C3)C(C)(C)O)O 9032

CC1=CC(=O)C2=C(O1)C=C3C=CC=C(C3=C2O)O[C@H]4[C@@H]([C@H]([C@@H]([C@H](O4)CO)O)O)O 1313

CC1=CC(C(=C(C)C)C(=O)C1)C/C(=C/COC(=O)C)/C 6425

CC1=CC(C(C(C1)C(=O)C2=C(C3=C(C=C2)OC(CC3)(C)C)O)C4=C(C=C(C=C4)O)O)C5=C(C=C(C6=C5OC(=C(C6=O)CC=C(C)C)C7=C(C=C(C=C7)O)O)O)O 5789

CC1=CC(C(C(C1)C2=C(C=C(C=C2)O)O)C(=O)C3=C(C4=C(C=C3)OC(C=C4)(C)C)O)C5=C(C=C(C=C5O)C6=CC7=C(O6)C=C(C=C7)O)O 5847

CC1=CC[C@@H](C([C@@H]1CC/C(=C/CC/C=C(\C)/CC/C=C(\C)/CCC=C(C)C)/C)(C)C)O 139

CC1=CC[C@@H](CC1O)C2=COC(=C2)CC(C)C 935

CC1=CC[C@H](CC1)[C@@]2(CC[C@@H](C(O2)(C)C)O)C 948

CC1=CC[C@H](CC1)C(=C)C 9418

CC1=CC[C@H](CC1)C(=C)CCC=C(C)C 9764

CC1=CC[C@H](OC1=O)[C@@H](C)[C@H]2CC[C@@]3([C@@]2(CC[C@]45[C@H]3CC[C@@H]6[C@]4(C5)CC[C@@H]([C@@]6(C)C(=O)O)O[C@H]7[C@@H]([C@H]([C@@H]([C@H](O7)CO)O)O)O)C)C 17

CC1=CC[C@H]2[C@@]([C@@H]1COC3=CC4=C(C=C3)C=CC(=O)O4)(CCC(=O)C2(C)C)C 1668

CC1=CC=C(C=C1)[C@@H](C)CC(=O)C=C(C)C 9355

CC1=CC=C(C=C1)[C@]2(CCCC2(C)C)C 1842

CC1=CC=C(C=C1)[C@H](C)CC(C=C(C)C)O 950

CC1=CC=CC=C1C(=O)C 5460

CC1=CC2=C(C=C[C@@](O2)(C)CC/C=C(\C)/CC/C=C(\C)/CO)C(=C1)O 1993

CC1=CC2=C(C=C1)NC3=C2C=CC4=C3C=CC(O4)(C)CCC(C)(C)O 5173

CC1=CC2=C(C3=CC(=CC(=C3C(=C2C(=O)O1)O)O)OC)OC 6503

CC1=CC23CC(=O)C4[C@]5(CCCC46C2CC1CC3C6N7C5OCC7)C 8044

CC1=CC2C(CC3C1C(CC3(C)O)OC(=O)C)C(=C)C(=O)O2 3186

CC1=CC2C(CCC3N2C(C1)CC4N3C(=O)CCC4)O 1271

CC1=CCC(CC1)[C@](C)(CCC=C(C)C)O 947

CC1=CCC[C@]2(C1C(=O)[C@@H](CC2)C(C)C)C 4229

CC1=CCC2(CCC(=C2CC1)C(C)C)C 1987

CC1=CCCC([C@H]1CC2=C(C=C3C(=C2O)C(=O)C(=C(O3)C4=CC=C(C=C4)O)O)O)(C)C 8835

CC1=CCSS1 5529

CC1=CN[11C](=O)NC1=O 10748

CC1=CO[C@@H](C2C1([C@H](C[C@@]2(C)O)O)O)O[C@H]3[C@@H]([C@@H]([C@@H]([C@H](O3)CO)O)O)O 4762

CC1=COC2=C1[C@H]3C=C(CC[C@H]4[C@@](C2)(O4)C)C(=O)O3 7139

CC1=COC2=C1C(=O)[C@@H]([C@](C2)(C)C=C)C(=C)C 2717

CC1=COC2=C1C(=O)C(=O)C3=C2C=CC4=C3CCCC4(C)C 9310

CC1=NC=C(C(=C1O)C=O)COP(=O)([O-])[O-] 9363

CC12C3C4C(C(C1(CC5C2(O5)C(=O)O3)O)C(=O)O4)C(C)(CO)O 2469

CC12CC3C(C4C=C(C5C(C1O2)O5)C(=O)O4)C(=C)C(=O)O3 5727

CC12CCC[C@@]3([C@@H]1CCC(=C)[C@H]3CCC4=COC=C4)C(=O)OC2 7000

CC12CCN(C1N(C3=C2C=C(C=C3)OC(=O)NC)C)C.C1=CC=C(C(=C1)C(=O)O)O 2875

CC1C(=O)N2CCCC2C(=O)NC(C(=O)N3CCCC3C(=O)NC(C(=O)N4CCCC4C(=O)NC(C(=O)NC(C(=O)N1)CC5=CC=CC=C5)CC6=CC=CC=C6)CC7=CC=CC=C7)CC(C)C 7116

CC1C(C(C(C(O1)OC2=CC(=CC(=C2O)C)C3CC(=O)C4=C(C=C(C=C4O3)O)O)O)O)OC5C(C(C(C(O5)CO)O)O)O 5367

CC1C(C(C(C(O1)OC2C(C(C(OC2OC3=CC(=C4C(=O)C[C@H](OC4=C3)C5=CC(=C(C=C5)O)O)O)CO)O)O)O)O)O 6018

CC1C(C(C(C(O1)OC2C(C(C(OC2OC3C(C(COC3OC4CCC5(C(C4(C)C)CCC6(C5CC=C7C6(CCC8(C7CC(CC8)(C)C)C(=O)O)C)C)C)O)O)CO)O)O)O)O)O 7303

CC1C(C(C(C(O1)OC2C(C(C(OC2OC3CCC4C(=CCC5C4(C(=O)CC6(C5(CC(C6C7(C(=O)C=C(O7)C(C)C)C)O)C)C)C)C3(C)C)CO)O)O)O)O)O 6800

CC1C(C(C(C(O1)OC2C(C(COC2OC3CCC4(C(C3(C)C)CCC5(C4CC=C6C5(CCC7(C6CC(=C)CC7)C(=O)OC8C(C(C(C(O8)O)COC9C(C(C(C(O9)CO)O)O)O)O)O)C)C)C)O)O)O)OC1C(C(C(C(O1)CO)O)O)O)O 9214

CC1C(C(C(C(O1)OC2C(OC(CC2OC)OC3C(OC(CC3OC)OC4C(OC(CC4OC)OC5C(OC6(CC5OC)COC7CC(OC(C7OO6)C)OC(C)C8(CCC9C8(CCC1C9CC=C2C1(CCC(C2)O)C)C)O)C)C)C)C)O)OC)O 6662

CC1C(C(CC(O1)OC2CCC3(C(C2)CCC4(C3CC(C5(C4(CCC5(C(C)O)O)O)C)OC(=O)CC(C)C)O)C)OC)OC6CC(C(C(O6)C)OC7C(C(C(C(O7)C)OC8C(C(C(C(O8)CO)O)O)O)OC)O)OC 2603

CC1C(C1C(C)C)CC(C)C2CCC3C2(CCC4C3CC=C5C4(CCC(C5=O)O)C)C 4752

CC1C(C2=C(O1)C=C3C(=C2O)C(=O)C4=CC5=C(C(=C4O3)O)OC(C=C5)(C)C)(C)C 3315

CC1C(C2=C3C(=C(C=C2O1)O)C(=O)C(=C(O3)C4=CC5=C(C(=C4)O)OC(C=C5)(C)C)O)(C)C 1019

CC1C(C2=C3C(=C(C=C2O1)O)C(=O)CC(O3)C4=CC=C(C=C4)O)(C)C 8833

CC1C(C2=CC(=C(C(=C2C1=O)C)CCCl)C)O 7186

CC1C(C2=CC(=C(C(=C2C1=O)C)CCO)C)OC3C(C(C(C(O3)CO)O)O)O 9084

CC1C(C2=CC(=C(C(=C2C1=O)C)CCO)CO)O 7191

CC1C[C@@]23CCC4[C@](C2CC(=O)[C@@H]1C3)(CC[C@H](C4(C)C)O)C 6363

CC1C[C@@]23CCC4[C@](C2CC(=O)[C@@H]1C3)(CC[C@H](C4(C)C)O[C@@H]5[C@@H]([C@H]([C@@H](CO5)O)O)O)C 6364

CC1C[C@]2(C3C4C1(C5C=C(C(=O)[C@]5([C@@H]([C@@]6([C@H]4O6)CO)O)O)C)O[C@@](O3)(O2)C7=CC=CC=C7)C(=C)C 1972

CC1C=C(C(=O)[C@]2(C1C[C@@H]3[C@@]4(C2C([C@H]([C@@]([C@@H]4CC(=O)O3)(C)OC(=O)C)OC(=O)C)O)C)C)OC 4717

CC1C=CSS1 5531

CC1C2=C(CN(CCC3=CC4=C(C=C3C1=O)OCO4)C)C5=C(C=C2)OCO5 1726

CC1C2C(C/C(=C/CC(/C(=C/C2OC1=O)/C)OC(=O)C)/C)OC(=O)C 5729

CC1C2C(CC1=O)C(=COC2O[C@H]3[C@@H]([C@H]([C@@H]([C@H](O3)CO)O)O)O)C(=O)OC 2114

CC1C2C(CC3C2(CCC4C3CCC5C4(CCC(C5)O)C)C)OC16CCC(CO6)CO 829

CC1C2CC3C1(CCCC3(C2)C(=C)C)C 11150

CC1C2CC3C45COC(C4C(C(=O)O3)OC(=O)CC(C)C(C)(C)OC(=O)C)(C(C(C5C2(C=C(C1=O)OC6C(C(C(C(O6)CO)O)O)O)C)O)O)C(=O)OC 4489

CC1C2CN3CCCC14C3C25C(C(C4)C(=O)OC)NC6=CC=CC=C56 2415

CC1CC(=O)C(=C[C@H]1O)C(C)(C)O 7727

CC1CC(=O)C2[C@@](C1(CCC3=COC=C3)O)(CCCC2(C)C)C 2409

CC1CC(C(C2(C13CC(C(C2OC(=O)C)OC(=O)C)C(O3)(C)C)COC(=O)C)OC(=O)C4=CC=CC=C4)OC(=O)C 4685

CC1CC(C2C1COC(=O)C2CC(C)C3=NC4CC5(C6CC3C4CO6)C7=CC=CC=C7N(C5=O)OC)O 2675

CC1CC2(C(C1OC(=O)C)C=C(C(CC(C(CC=C(C2=O)C)(C)C)OC(=O)C)OC(=O)C3=CC=CC=C3)C)O 7201

CC1CC2(C(C1OC(=O)C3=CC=CC=C3)/C=C(/C(CC(C(/C=C\C(C2OC(=O)C)C)(C)C)OC(=O)C)OC(=O)C)\C)O 9585

CC1CC2(C(O2)C)C(=O)OC3CCN(C/C=C(\C3=O)/COC(=O)C1(C)O)C 6693

CC1CC2(CC(=CCCC2C1(CC/C(=C/CO)/CO)CO)C)CO 6998

CC1CC2[C@H](OC3([C@H]1[C@]4(CC[C@@]56C[C@@]57CC[C@@H](C(C7CCC6C4(C3OC)C)(C)C)O)C)O2)C(C)(C)O 5501

CC1CC2=CC(=C(C(=C2C1=O)C)CCO[C@H]3[C@@H]([C@H]([C@@H]([C@H](O3)CO)O)O)O)C 7174

CC1CC2=CC(=C(C(=C2CO1)O)C)OC 2518

CC1CC2=CC(=C(C=C2C(C1C)C3=CC(=C(C=C3)O)OC)O)OC 3534

CC1CC2C(C(C3(C1C(CC3OC(=O)C)OC(=O)C)C)O)C(=C)C(=O)O2 4180

CC1CC2C(C(C3(O2)CCC4C5CC(=O)C6CC(CCC6(C5C(C4=C3C)O)C)O)C)NC1 9221

CC1CC2C(C(C3(O2)CCC4C5CC=C6CC(CCC6(C5CC4=C3C)C)O)C)NC1 1918

CC1CC2CC3CCCC(=O)N3C4N2C(C1)C(CC4)O 5061

CC1CCC(=O)CC1 5513

CC1CCC(C(=C1)O)C(=C)C 10971

CC1CCC(C(C1)OC(=O)C2OC(CS2)N3C=CC(=NC3=O)N)C(C)C 7233

CC1CCC(C1)(C)C 8725

CC1CCC(C2(C13C(C(C(C2OC(=O)C)OC(=O)C)C(O3)(C)C)OC(=O)C4=CC=CC=C4)COC(=O)C5=CN=CC=C5)OC(=O)C 4683

CC1CCC(C2(C13CC(C(C2OC(=O)C)OC(=O)C)C(O3)(C)C)COC(=O)C)OC(=O)C4=CC=CC=C4 4686

CC1CCC(C2(C13CC(C(C2OC(=O)C)OC(=O)C4=CN=CC=C4)C(O3)(C)C)COC(=O)C)OC(=O)C5=CC=CC=C5 4684

CC1CCC(CC1)C(C)CCC=C(C)C 2515

CC1CCC=C(C12CC[C@H](C2)C(C)(C)O)C(=O)O 11264

CC1CCC2(C(C3[C@]4(CCC5C(C4CC3(O2)OC)CC=C6[C@@]5(CC[C@@H](C6)O)C)C)C)OC1 908

CC1CCC2(C(C3C(O2)CC4C3(C(=O)CC5C4CCC6C5(CCC(C6)O[C@H]7[C@@H]([C@H]([C@H]([C@H](O7)CO)O[C@H]8[C@@H]([C@H]([C@@H]([C@H](O8)CO)O)O[C@H]9[C@@H]([C@H]([C@@H](CO9)O)O[C@H]2[C@@H]([C@H]([C@@H]([C@H](O2)CO)O)O[C@H]2[C@@H]([C@H]([C@@H]([C@H](O2)CO)O)O)O)O)O)O[C@H]2[C@@H]([C@H]([C@@H]([C@H](O2)CO)O)O)O)O)O)C)C)C)OC1 2571

CC1CCC2(C(C3C(O2)CC4C3(C(=O)CC5C4CCC6C5(CCC(C6)OC7C(C(C(C(O7)CO)OC8C(C(C(C(O8)CO)O)OC9C(C(C(C(O9)CO)O)O)O)OC2C(C(C(C(O2)CO)O)OC2C(C(C(CO2)O)O)O)O)O)O)C)C)C)OC1 214

CC1CCC2(C(C3C(O2)CC4C3(CCC5C4CCC6C5(CCC(C6)O[C@H]7[C@@H]([C@H]([C@@H]([C@H](O7)CO)O)O)O[C@H]8[C@@H]([C@H]([C@@H](CO8)O[C@H]9[C@@H]([C@H]([C@@H]([C@H](O9)CO)O)O)O)O)O)C)C)C)OC1 661

CC1CCC2(C(C3C(O2)CC4C3(CCC5C4CCC6C5(CCC(C6)O[C@H]7[C@@H]([C@H]([C@@H]([C@H](O7)CO)O)O)O[C@H]8[C@@H]([C@H]([C@H](CO8)O[C@H]9[C@@H]([C@@H]([C@@H]([C@@H](O9)O[C@H]2[C@@H]([C@H]([C@@H]([C@H](O2)CO)O)O)O)O)O)O)O)O)C)C)C)OC1 698

CC1CCC2(CCC3(C(=CCC4C3(CCC5C4(CC(C(C5(C)CO)O)O)C)C)C2C1C)C)COC6C(C(C(C(O6)COC7C(C(C(C(O7)CO)C)O)O)O)O)O.CC1C(C(C(C(O1)O)O)O)O 676

CC1CCC2(CCC3(C(=CCC4C3(CCC5C4(CCC(C5(C)C)O)C)C)C2C1C)C)C 9451

CC1CCC2C(C(=O)OC2C3C1CCC3C)C 11342

CC1CCCC(=O)CCCC=CC2=C(C=C(C=C2C(=O)O1)O)O 9262

CC1COC2=C(C1(C)CO)C(=O)C3=C(C2=O)C4=C(C=C3)C(=CC=C4)C 1966

CCC(=CC(=O)OC1C2C34COC2(C(C(C3C5(C=C(C(=O)C(C5CC4OC1=O)C)OC6C(C(C(C(O6)CO)O)O)O)C)O)O)C(=O)OC)C 4490

CCC(=CC(=O)OC1C2C34COC2(C(C(C3C5(C=C(C(=O)C(C5CC4OC1=O)C)OC6C(C(C(C(O6)CO)O)O)O)C)O)O)C(=O)OC)C(C)(C)O 4488

CCC(=O)O[C@H]1CC(=C2[C@H](C[C@@]3(CC[C@@H](C(=C)[C@H]3[C@@H]([C@H]1C2(C)C)OC(=O)C)OC(=O)C)C)OC(=O)C)C 11936

CCC(=O)OC1[C@H]([C@@]2([C@H]([C@H]([C@H]3[C@](C24[C@@H](C1[C@@](O4)(COC(=O)C5=C([C@@H]([C@@H](C(=O)O3)C)C)N=CC=C5)C)OC(=O)C6=CC=CC=C6)(C)O)O)OC(=O)C7=CC=CC=C7)COC(=O)C)OC(=O)C 2939

CCC(C)/C=C(\C)/C=C/C=C/C(=O)C1=C(C(=CN(C1=O)O)C2=CC=C(C=C2)O)O 835

CCC(C)C(=O)OC(C)C1(CCC2(C1(C(CC3C2CCC4C3(CCC(C4)O)C)O)C)O)O 2604

CCC(C)C(=O)OC[C@@]1([C@@H]2[C@]1(C[C@H]([C@]3([C@H]2C=C(C[C@]4([C@H]3C=C(C4=O)C)O)COC(=O)C)O)C)OC(=O)CC5=CC=CC=C5)C 1209

CCC(C)C(C(=O)NC(C(C)C)C(=O)N1CCC2C1C(=O)NC(C(=O)N/C=C/C3=CC=C(O2)C=C3)CC4=CC=CC=C4)N(C)C 5285

CCC(C)C(C(=O)NC1C(OC2=CC=C(C=C2)C=CNC(=O)C(NC1=O)CC3=CC=CC=C3)C(C)C)N(C)C 7763

CCC(C)CCC(=O)N[C@@H](C1=CC=CC=C1)[C@H](C(=O)O[C@H]2C[C@]3([C@H]([C@H]4[C@@]([C@H](C[C@@H]5[C@]4(CO5)OC(=O)C)O)(C(=O)[C@@H](C(=C2C)C3(C)C)OC(=O)C)C)OC(=O)C6=CC=CC=C6)O)O 8364

CCC(C)OC(=O)C1=CC(CC2=C1C=CC=C2/C(=C/C(=O)O)/O)C(=O)O 11978

CCC(C[C@H]1C=CC[C@H](N1C)CC(=O)C2=CC=CC=C2)O 4352

CCC(C=C)O 6635

CCC(CC([C@@](C)(C1CCC2([C@@]1(CCC3C2=CC(=O)[C@H]4[C@@]3(C[C@@H]([C@@H](C4)O)O)C)C)O)O)O)C(C)(C)O 4840

CCC/C=C/CO 3783

CCC/C=C/COC(=O)C1=CC=CC=C1 3784

CCC/C=C\1/C2=C(C(=CC=C2)O)C(=O)O1 7806

CCC[C@H](/C=C/C=C/C=C/C#CC#CCCCO)O 1483

CCC[C@H](CC)O 3779

CCC[NH3+] 7074

CCC\1=C(/C/2=C/C3=C(C(=C(N3)/C=C\4/[C@H]([C@@H](C(N4)C5=C6C(=C([C@@H]5C(=O)OC)O)C(=C(N6)/C=C1\N2)C)CCC(=O)OC/C=C(/C)\CCC[C@H](C)CCC[C@H](C)CCCC(C)C)C)C)C=C)C 6752

CCC1(C2=C(COC1=O)C(=O)N3CC4=C(C3=C2)N=C5C=CC(=CC5=C4)OC)O 5389

CCC1=C(C=C(C=C1)O)O 2917

CCC1=C2C=C3C(=C4C(=O)C(C(=C5C(C(C(=N5)C=C6C(=C(C(=CC(=C1C)N2)N6)C=C)C)C)CCC(=O)OC)C4=N3)C(=O)OC)C 5646

CCC1=C2C=C3C(=C4C(=O)CC(=C5[C@@H]([C@H](C(=N5)C=C6C(=C(C(=CC(=C1C)N2)N6)C=C)C)C)CCC(=O)O)C4=N3)C 7235

CCC1=N[C@H]2C[C@@]3([C@@H]4C[C@@H]1[C@@H]2CO4)C5=CC=CC=C5N(C3=O)OC 3862

CCC1=NC=C(C2=C1NC3=C2C=CC=C3O)OC 6804

CCC12C=CCN3C1C4(CC3)C(C(C2C(=O)OC)(C(=O)OC)O)N(C5=CC=CC=C45)C 9022

CCC1C(=O)CC2[C@@]1(CCC3C2CC=C4C3(CC(C(C4)NC)O)C)C 4696

CCC1C(COC1=O)CC2=CN=CN2C.[N+](=O)(O)[O-] 6837

CCC1CC(OC(=O)C1)CCCCCCCCCCCCCCCCC(C(C)CC)O[C@@H]2[C@@H]([C@H]([C@@H]([C@H](O2)CO[C@H]3[C@@H]([C@@H]([C@H]([C@@H](O3)C)O)O)O)O)O)O 792

CCC1CN2CCC3=C(C2C[C@@H]1/C(=C\OC)/OC(=O)C)NC4=C3C(=CC=C4)O 3212

CCC1CN2CCC3=CC(=C(C=C3C2CC1/C=C\4/C5=CC(=C(C=C5CCN4)O)OC)OC)OC 7164

CCCC(=O)C1=C(C(=C(C=C1O)OC)C)O 712

CCCC(=O)C1=C(C2=C(C(=C1O)CC(C(=C)C)O)OC(=O)C=C2C3=CC=CC=C3)O 7302

CCCC(=O)OC[C@@H]1[C@H]([C@@H]([C@H]([C@@H](O1)OC2CCC3(C(C2(C)CO)CCC4(C3CC=C5C4(CC(C6(C5CC(CC6)(C)C)C(=O)O)O)C)C)C)O)O)O 9081

CCCC(=O)OCC 2892

CCCC/C=C/CC/C=C/CCCCCCCC(=O)OC 5591

CCCC/C=C\CCCCCCCCCC1=C(C(=O)C(=C(C1=O)OC(=O)C)C)OC 5377

CCCC[11C](=O)O 9694

CCCC[C@H](C1=CC=CC=C1)O 10970

CCCC=C1CCCCC1 1100

CCCC1OC[C@]23CC[C@](CC2C4=CCC5[C@]6(CC[C@@H](C(C6CC[C@]5(C4(C[C@@H]3O1)C)C)(C)C)O[C@H]7[C@@H]([C@H]([C@H](CO7)O[C@H]8[C@@H]([C@H]([C@@H]([C@H](O8)CO)O)O)O[C@H]9[C@@H]([C@H]([C@@H](CO9)O)O)O)O)O[C@H]1[C@@H]([C@H]([C@@H]([C@H](O1)CO)O)O)O)C)(C)CO 5087

CCCCC(=O)C1=CC=CC=C1 10003

CCCCC.CCCC=C 2551

CCCCC/C=C/C=C/C=O 9474

CCCCC/C=C\C=C\C(=O)NCC(C)C 10630

CCCCC[14C](=O)O 9546

CCCCC[C@H]1CCCCCCCCCC(=O)OC2[C@@H](C(O[C@H](C2O[C@H]3[C@H](C([C@H](C(O3)C)OC(=O)[C@@H](C)CC)O[C@H]4[C@H](C([C@H](C(O4)C)O)O)O)OC(=O)[C@H](C)CC)OC5[C@@H](O1)OC([C@@H](C5O)O)C)CO)O 8652

CCCCC1=CC(=CC(=C1)C)C 2507

CCCCC1C2=C(C=CC(=C2C(=O)O1)O)O 2422

CCCCCC(=O)CCC(=O)OC 5634

CCCCCC(=O)O[C@H]1[C@H]2[C@@]([C@H](C[C@@H]3[C@]2(CO3)OC(=O)C)OC(=O)C)([C@@H]([C@@H](C4=C([C@H](C[C@@]1(C4(C)C)O)OC(=O)C)C)OC(=O)C)OC(=O)C)C 800

CCCCCC(=O)OC1CCC2(C3CCC4C5C(CCC5(CCC4(C3(CCC2C1(C)C)C)C)C)CC)C 1936

CCCCCC(CC)O 6293

CCCCCC/C=C\CCCCCCCCCC1=CC(=O)C=C(C1=O)OC 4219

CCCCCC1=C(C(=CC(=C1O[C@H]2[C@@H]([C@H]([C@@H]([C@H](O2)CO)O)O)O)O)O)O[C@H]3[C@@H]([C@H]([C@@H]([C@H](O3)CO)O)O)O 1239

CCCCCC1=CC2=C(C=CC(O2)(C)CCC=C(C)C)C(=C1)O 1212

CCCCCCC/C=C\CO 10978

CCCCCCC[C@@H]([C@@H]1CC[C@H](O1)[C@H](CCCCCCCCCC[C@@H](CC(=O)CC[C@H](CC2=C[C@@H](OC2=O)C)O)O)O)O 5786

CCCCCCC1=CC=CC=C1 6741

CCCCCCCC 6292

CCCCCCCC(=O)[O-].[Na+] 9349

CCCCCCCC(=O)CCC(C)C 5532

CCCCCCCC(=O)CCC1=CC(=C(C=C1)O)OC 6557

CCCCCCCC(=O)NCC1=CC(=C(C=C1)O)OC 2073

CCCCCCCC(C1CCC(O1)C(CCCCCCCCCCC(CC(=O)CCC(CC2=CC(OC2=O)C)O)O)O)O 5787

CCCCCCCC(CC)C1=CC=CC=C1 6734

CCCCCCCC/C=C\CCCCCCCC(=O)OC 5625

CCCCCCCC/C=C\CCCCCCCC(=O)OC[C@H](COP(=O)(O)[O-])OC(=O)CCCCCCC/C=C\CCCCCCCC.[Na+] 2592

CCCCCCCC[C@H](C)O 10960

CCCCCCCCC(C)C1CCCCC1 1903

CCCCCCCCC[C@H](C)OC(=O)C 8858

CCCCCCCCCC(=O)/C=C(/C=C\C1=CC(=C(C=C1)O)OC)\O 2103

CCCCCCCCCC(=O)CCCCCCCCC 11219

CCCCCCCCCC(=O)OCC1=CC2C3C(C3(C)C)CC(C4(C2=O)C=C(C(C4(C1O)O)O)C)C 2060

CCCCCCCCCC(CC)C1CCCCC1 1904

CCCCCCCCCC(CCC)C1=CC=CC=C1 6744

CCCCCCCCCC/C=C\C(=O)O 10788

CCCCCCCCCCCC(=O)O[C@H]1CC[C@@]2(C3CC=C4C5CC(CC[C@@]5(CCC4([C@@]3(CCC2(C1(C)C)C)C)C)C)(C)C)C 11234

CCCCCCCCCCCC(CCCCCCCCCCC)O 8655

CCCCCCCCCCCC[C@@H]([C@@H]1CC[C@H](O1)[C@@H](CCCCC(CCCCC[C@H](CC2=C[C@@H](OC2=O)C)O)O)O)O 680

CCCCCCCCCCCC[C@@H]1[C@@H](CC[C@@H](O1)[C@@H](CCCC[C@@H](CCCCC[C@H](CC2=C[C@@H](OC2=O)C)O)O)O)O 7225

CCCCCCCCCCCC[C@H]([C@H]1CC[C@@H](O1)[C@@H](C[C@@H](CCCCCCCCCC(CCC2=CC(OC2=O)C)O)O)OC(=O)C)O 8895

CCCCCCCCCCCC[C@H]([C@H]1CC[C@@H](O1)[C@@H](CC[C@@H](CCCCCCC[C@H](CC2=C[C@@H](OC2=O)C)O)O)O)O 4491

CCCCCCCCCCCC1=C2C(=CC(=C1)O)C(=C(C(=O)C2=O)CCCCCCCCCCC)O 2441

CCCCCCCCCCCCC(C(CCC(C(CCCCCCC(CCC[C@H](CC1=C[C@@H](OC1=O)C)O)O)O)O)O)O 3251

CCCCCCCCCCCCCC(CCCC)O 6288

CCCCCCCCCCCCCC[C@@H]1[C@@H](CC[C@@H](O1)[C@@H](CC[C@@H](CCCCC[C@H](CC2=C[C@@H](OC2=O)C)O)O)O)O 7223

CCCCCCCCCCCCCC1=C(C(=O)C(=C(C1=O)OC(=O)C)C)OC 5378

CCCCCCCCCCCCCCC#C 11088

CCCCCCCCCCCCCCC(C(C(CO)[NH3+])O)O 6792

CCCCCCCCCCCCCCC(C(CCCCCCC(CCCCC[C@H](CC1=C[C@@H](OC1=O)C)O)O)O)O 3252

CCCCCCCCCCCCCCCC(=O)OC/C=C(\C)/C=C/C=C(\C)/C=C/C1=C(CCCC1(C)C)C.CC1=CC2=C(C=C1C)N(C=N2)C3C(C(C(O3)CO)OP(=O)([O-])OC(C)CNC(=O)CCC\4(C(C5=N/C4=C(\C6=N/C(=C\C7=N/C(=C(\C8=NC5(C(C8CCC(=O)N)(C)CC(=O)N)C)/C)/C(C7CCC(=O)N)(C)CC(=O)N)/C(C6CCC(=O)N)(C)C)/C)CC(=O)N)C)O.CC1=CC2=C(C=C1C)N(C3=NC(=O)NC(=O)C3=N2)C[C@@H]([C@@H]([C@@H](CO)O)O)O.CC1=C(C(=C2CC[C@@](OC2=C1C)(C)CCC[C@H](C)CCC[C@H](C)CCCC(C)C)C)O.CC1=C(C(=O)C2=CC=CC=C2C1=O)C/C=C(/C)\CCCC(C)CCCC(C)CCCC(C)C.CC1=C(SC=[N+]1CC2=CN=C(N=C2N)C)CCO.CC1=NC=C(C(=C1O)CO)CO.C[C@H](/C=C/[C@H](C)C(C)C)[C@H]1CC[C@@H]\2[C@@]1(CCC/C2=C\C=C/3\C[C@H](CCC3=C)O)C.CC(C)(CO)[C@H](C(=O)NCCC(=O)O)O.[C-]#N.C1[C@H]2[C@@H]([C@@H](S1)CCCCC(=O)O)NC(=O)N2.C1=CC(=CN=C1)C(=O)N.C1=CC(=CC=C1C(=O)N[C@@H](CCC(=O)O)C(=O)O)NCC2=CN=C3C(=N2)C(=O)N=C(N3)N.C([C@@H]([C@@H]1C(=C(C(=O)O1)O)O)O)O.[Co+2] 7232

CCCCCCCCCCCCCCCC(=O)OC[C@@H]1[C@H]([C@@H]([C@H]([C@@H](O1)O[C@H]2CC[C@@]3(C4CC[C@@]5([C@H](CCC5C4CC=C3C2)[C@H](C)CC[C@H](CC)C(C)C)C)C)O)O)O 10100

CCCCCCCCCCCCCCCC[14CH2]O 3719

CCCCCCCCCCCCCCCC1CC(=O)NCCCNCCCCNCCCN1 1049

CCCCCCCCCCCCCCCCC1=C(C(=O)C=C(C1=O)OC)O 4213

CCCCCCCCCCCCCCCCCCCC1=C(C(=O)C=C(C1=O)OC)O 4215

CCCCCCCCCCCCCCCCCCCCC 3708

CCCCCCCCCCCCCCCCCCCCCCCC1=CC(=CC(=C1)OC)O 8254

CCCCCCCCCCCCCCCCCCCCCCCCC 6615

CCCCCCCCCCCCCCCCCCCCCCCCCCCCCCC 3710

CCCCCCCCCCCCCCCCCCCCCCCCCCCCCCOC(=O)CCCCCCCCCCCCCCCCCCCCCCC 5337

CCCCCCCCCCCCCCCCCCCCCCCCCCCO 3714

CCCCCCCCCCCCCCCCCCCCCCCCCCO 1396

CCCCCCCCCCCCCCCCCCCCCCCCCCOC(=O)CCCCCCCCCCCCCCCCCCCCCCCCC 1397

CCCCCCCCCCCCCCCCCCOC1(C2=CC(=CC(=C2C(=O)C3=C(C=C(C=C31)O)O)O)CO)O 7387

CCCCCOC(=O)CCC 392

CCCCOC(=O)C1C(C(C(C(O1)OC2CCC3(C(C2(C)C)CCC4(C3CC=C5C4(C(C(C6(C5CC(C(C6OC(=O)C(C)CC)OC(=O)C=C(C)CCC=C(C)C)(C)C)CO)O)O)C)C)C)OC7C(C(C(C(O7)CO)O)O)O)O)OC8C(C(C(O8)CO)O)O 8195

CCCCOC(=O)CC(C)C(=O)NC1=CC=CC=C1C(=O)OC[C@@]23CCC(C45C2C([C@](C4N(C3)CC)([C@]6(C[C@@H](C7CC5C6C7OC)OC)O)O)OC)OC 7001

CCCNC(=O)CCCC1CCC2(C1(C(CC3C2C(CC4(C3(CCC(C4)O)C)C)O)O)C)C 8286

CCCSS/C=C\C 7070

CCCSSCCC 2562

CCN(CC)C(=O)/C(=C\C1=CC(=C(C(=C1)O)O)[N+](=O)[O-])/C#N.C[C@](CC1=CC(=C(C=C1)O)O)(C(=O)O)NN.C1=CC(=C(C=C1C[C@@H](C(=O)O)N)O)O.O 9411

CCN(CC)CCC1=NC=C(C2=C1NC3=C2C=CC=C3O)OC 6805

CCN1C[C@@]2([C@@H](CC(C34C2[C@H](C(C31)[C@]5(C[C@@H]([C@]6(CC4C5C6OC(=O)C7=CC=CC=C7)O)OC)O)OC)OC)O)COC 5018

CCN1C[C@@]2(C(CC(C34C2C(C(C31)[C@]5(C([C@@H]([C@]6(CC4C5C6OC(=O)C7=CC=CC=C7)O)OC)O)OC)OC)OC)O)COC 877

CCN1C[C@@]2(CC[C@@H]([C@@]34[C@@H]2[C@@H]([C@@](C31)([C@]5(C[C@@H]([C@H]6C[C@@H]4[C@@H]5[C@H]6OC)OC)O)O)OC)OC)COC(=O)C 8653

CCN1C[C@@]2(CC[C@@H]([C@@]34[C@@H]2[C@@H]([C@](C31)([C@]5(C[C@@H]([C@H]6C[C@@H]4[C@@H]5[C@H]6O)OC)O)O)OC)OC)COC 1023

CCN1C[C@@]2(CC[C@@H]([C@@]34[C@@H]2[C@@H]([C@](C31)([C@]5(C[C@@H](C6CC4C5[C@H]6OC)OC)O)O)OC)OC)CO 5063

CCN1C[C@@]2(CC[C@H](C34[C@@H]2C(C(C31)([C@]5(C[C@@H](C6CC4C5[C@H]6OC)OC)OC)O)OC)OC)COC(=O)C7=CC=CC=C7NC(=O)CCC(=O)O 7198

CCN1C[C@@]2(CCC(C34[C@@H]2[C@@H]([C@]5([C@@H]31)[C@]6(C[C@@H]([C@@H]7C[C@@H]4[C@@H]6C7OC)OC)OCO5)OC(=O)C)OC)C 2154

CCN1C[C@@]2(CCC(C34[C@@H]2CC(C31)[C@]5(C[C@@H]([C@H]6C[C@@H]4[C@@]5([C@H]6OC)O)OC)O)OC)OC(=O)C7=CC=CC=C7N 7200

CCN1C[C@@]2(CCC(C34C2[C@@H]([C@](C31)([C@]5(C[C@@H](C6CC4C5C6OC)OC)O)O)OC)OC)COC 2143

CCN1C[C@@]2(CCC(C34C2C([C@](C31)([C@]5(C[C@@H](C6CC4C5C6OC)OC)O)O)OC)OC)COC(=O)C7=CC=CC=C7NC(=O)CC(C)C(=O)N 2152

CCN1C[C@@]2(CCC(C34C2C(C(C31)([C@]5(C[C@@H](C6CC4C5C6O)OC)O)O)OC)OC)COC(=O)C7=CC=CC=C7N 2141

CCN1C[C@@]2(CCC(C34C2C(C(C31)([C@]5(C[C@@H](C6CC4C5C6O)OC)OC)O)OC)O)CO 7003

CCN1CC2(C(CC(C34C2C(C(C31)C5(CC(C6(CC4C5C6OC(=O)C7=CC(=C(C=C7)OC)OC)O)OC)OC(=O)C)OC)OC)O)COC 7118

CCN1CC2(C=CC(C34C2C(C(C31)[C@]5(C[C@@H]([C@]6(CC4C5C6OC(=O)C7=CC=C(C=C7)OC)O)OC)OC(=O)C)OC)OC)COC 4871

CCN1CCC2=CC(=C(C=C2C1(C)C)O)O 2438

CCO[C@H]1[C@]([C@H]([C@@H]([C@H](O1)CO[C@H]2[C@@H]([C@@H]([C@H]([C@@H](O2)C)O)O)O)OC(=O)/C=C/C3=CC(=C(C=C3)O)O)O)(C4=CC(=C(C=C4)O)O)O 10972

CCOC(=O)CCCCCCCCCCCCCC(C)C 2903

CCOC(C)(C)C1(CCC2(C1C(CC3(C2CCC4C3(CCC5C4(CCC(C5)O)C)C)C)O)C)O 5767

CN(C)C(=NCCC[C@@H](C(=O)O)N)N 9353

CN(C)C.Cl 8720

CN(C)CCC1=CC(=C(C2=C1CCC3=CC4=C(C=C32)OCO4)OC)OC 8521

CN(C)CCC1=CC=C(C=C1)O.OS(=O)(=O)O 3853

CN(CCC1=C2C(=NC=N1)N(C=N2)[C@H]3[C@@H]([C@@H]([C@H](O3)CO)O)O)C4CCCCC4 7214

CN[C@@H](CC1=CC=CC=C1)C(=O)O 5644

CN[C@H]1[C@@H]([C@H]([C@@H](OC1C2[C@@H](O[C@H](C2(C=O)O)CO)O[C@@H]3[C@@H]([C@H]([C@H]([C@H]([C@@H]3O)O)N=C(N)N)O)N=C(N)N)CO)O)O 7374

CN\1C=CC=C/C1=C\[NH+]=O.CN1[C@@H]2CC[C@H]1CC(C2)OC(=O)C(CO)C3=CC=CC=C3.[Cl-] 755

CN1[C@H](CCC[C@@H]1C[C@@H](C2=CC=CC=C2)O)C[C@H](C3=CC=CC=C3)O 4969

CN1[C@H]2C[C@H](CC[C@@]23[C@@H](C1=O)C(C(=O)C4=CC5=C(C=C34)OCO5)CCC6=CC=C(C=C6)O)OC 6925

CN1C[C@@H](C[C@@H](C1)C2=CC=CC(=O)N2)CO 4687

CN1C[C@@H](C=C2[C@@H]1CC3=CNC4=CC=CC2=C34)CO 5085

CN1CC(CCC1CC(=O)C2=CC=CC=C2)CC(=O)C3=CC=CC=C3 4970

CN1CC[C@@]23[C@@]1(CC[C@@]24[C@H](C(=CC4=O)OC)O)C(=C(C(=O)C3)OC)OC 2719

CN1CC[C@@]23CC(=O)C(=C[C@@H]2[C@@H]1CC4=C3C(=C(C=C4C5=CC(=C(C6=C5C[C@H]7[C@@H]8[C@@]6(CCN7C)CC(=O)C(=C8)OC)O)OC)OC)O)OC 2565

CN1CC[C@]2([C@@H]1NC3=CC=CC=C32)C4=C5C(=CC=C4)[C@]6(CCN([C@H]6N5)C)[C@@]78CCN([C@@H]7NC9=C(C=CC=C89)[C@]12CCN([C@H]1NC1=CC=CC=C21)C)C 7246

CN1CC[C@]2([C@@H]1NC3=CC=CC=C32)C4=C5C(=CC=C4)[C@]6(CCN([C@H]6N5)C)C7=C8C(=CC=C7)[C@]9(CCN([C@H]9N8)C)[C@@]12CCN([C@@H]1NC1=C(C=CC=C21)[C@]12CCN([C@H]1NC1=CC=CC=C21)C)C 7163

CN1CC[C@]2([C@@H]1NC3=CC=CC=C32)C4=C5C(=CC=C4)[C@]6(CCN([C@H]6N5)C)C7=C8C(=CC=C7)[C@]9(CCN([C@H]9N8)C)[C@@]12CCN([C@@H]1NC1=CC=CC=C21)C 7247

CN1CC[C@]23[C@@H]4C5C(CC2=O)C(=CCOC5CC(=O)N4C6=CC=CC=C36)C1 4102

CN1CC2=C(C=CC3=C2OCO3)C4=C1C5=CC6=C(C=C5C=C4)OCO6 2406

CN1CC2=C(C=CC3=C2OCO3)C4C1C5=C(C6=C(C=C5CC4O)OCO6)OC 5395

CN1CCC(CC1)N(CC2=CC=CC=C2)C3=CC=CC=C3 6874

CN1CCC[C@@H]1CC(=O)C[C@H]2CCCN2C 1870

CN1CCC2=C([C@@H]1C[C@H]3C[C@H]4C5=C(CCN4C[C@@H]3C=C)C6=CC=CC=C6N5)NC7=CC=CC=C27 8884

CN1CCC2=C(C(=C3C=C2[C@@H]1CC4=CC(=C(C=C4)O)OC5=CC=C(CC6C7=CC(=C(C(=C7CCN6C)O3)OC)OC)C=C5)OC)OC 8512

CN1CCC2=C3[C@@H]1CC4=CC5=C(C=C4OC3=C(C=C2)O)OCO5 1833

CN1CCC2=C3C(=C(C=C2[C@@H]1CC4=CC(=C(C=C4)OC)OC5=CC=CC(=C5)C[C@H]6C7=C(CCN6C)C(=C8C(=C7O3)OCO8)OC)OC)OC 8511

CN1CCC2=CC(=C(C=C2[C@@H]1CC3=CC(=C(C=C3)OC)O)OC)OC 4813

CN1CCC2=CC(=C(C=C2[C@H]1CC3=CC(=C(C=C3)O)OC4=CC=C(C=C4)CC5C6=CC(=C(C=C6CCN5C)OC)OC)OC)OC 1994

CN1CCC2=CC(=C(C=C2[C@H]1CC3=CC(=C(C=C3)OC)OC)OC)OC 4814

CN1CCC2=CC(=C(C=C2[C@H]1CC3=CC=C(C=C3)OC4=C(C=CC(=C4)CC5C6=CC(=C(C=C6CCN5C)O)OC)O)OC)OC 1995

CN1CCC2=CC(=C(C=C2C1=O)O)OC 8525

CN1CCC2=CC(=C(C=C2C1=O)OC)OC 5507

CN1CCC2=CC(=C(C=C2C1CC3=CC(=C(C=C3)OC)O)OC)OC 4812

CN1CCC2=CC(=C(C=C2C1CC3=CC=C(C=C3)OC)OC4=C(C=CC(=C4)CC5C6=CC(=C(C=C6CCN5C)OC)O)O)OC 4347

CN1CCC2=CC(=C(C=C2C3[C@H]1C4=C(C(O3)O)C5=C(C=C4)OCO5)O)OC 5524

CN1CCC2=CC(=C(C3=C2C1CC34C=CC(=O)C(=C4)OC5=CC=C(C=C5)CC6C7=CC(=C(C=C7CCN6C)OC)OC)OC)OC 6504

CN1CCC2=CC(=C(C3=C4C=C(C(=CC4=CC1=C23)OC)OC)OC)OC 2104

CN1CCC2=CC(=C3C=C2C1CC4=CC=C(C=C4)OC5=C(C=CC(=C5)CC6C7=C(O3)C(=C(C=C7CCN6)OC)O)OC)OC 6229

CN1CCC2=CC(=C3C4=C2C1CC5=CC=C(C=C5)OC6=C(C=CC(=C6)CC7=NCCC8=CC(=C(O4)C(=C87)OC)O3)OC)OC 5341

CN1CCC2=CC3=C(C=C2[C@H]1C4C5=C(C6=C(C=C5)OCO6)C(=O)O4)OCO3 920

CN1CCC2=CC3=C(C=C2C1CC4=CC=C(C=C4)OC5=C(C=CC(=C5)CC6C7=C(C3)C(=C(C=C7CCN6C)OC)OC)O)OC 8516

CN1CCC23C=C(C(=O)C=C2C1CC4=CC(=C(C=C34)OC)O)OC 6507

CN1CCC23C1(CCC4=C2C(=C(C=C4)OC)O)C(=C(C(C3)O)OC)OC 3745

CN1CCCC1C2=C(C=CC3=C2NC4=C3CC[N+]5=C4C(=C(C(=C5)C=C)CC6C7=C(CCN6C)C8=CC=CC=C8N7)O)O.[Cl-] 1470

CO[C@@H]1C[C@@]23C(=CCN2CCC4=CC(=C(C=C34)OS(=O)(=O)CC(=O)O)O)C=C1 2849

CO[C@H]1[C@H]2[C@H](CC=C2CO)[C@H](C(=O)O1)CO 2462

COC(=O)/C=C/C=C/C1=CC2=C(C=C1)OCO2 5648

COC(=O)[C@@]1(CC2=CC[C@]13[C@]4(CC(=O)N(C4=O)CCC2)C5=C(N3C(=O)OC)C6=C(C=C5)OCO6)O 6584

COC(=O)[C@]1(C[C@@H]([C@@H]([C@H](C1)OC(=O)/C=C/C2=CC(=C(C=C2)O)O)O)O)O 5499

COC(=O)[C@H]1[C@H](CC[C@H]2[C@@H]1C[C@H]3C4=C(CCN3C2)C5=CC=CC=C5N4)O 311

COC(=O)C1=CC2=C(C(=CC3=C1NC4=CC=CC=C43)C(=O)OC)NC5=CC=CC=C52 1341

COC(=O)C1=CCCNC1 3554

COC(=O)C1=CO[C@@H]([C@@H]2[C@H]1C=C[C@@]2(CO)O)O[C@H]3[C@@H]([C@H]([C@@H]([C@H](O3)CO)O)O)O 3250

COC(=O)C1=CO[C@H](C2C1CC=C2CO)O 3273

COC(=O)C1=COC(C(C1CC=O)C=C)OC2C(C(C(C(O2)CO)O)O)O.C(Cl)(Cl)Cl 7768

COC(=O)C1=COC(C2C1C[C@@H]([C@@H]2COC(=O)C3C(C(C3C4=CC=C(C=C4)O)C(=O)OC[C@H]5[C@H](CC6C5C(OC=C6C(=O)OC)O[C@H]7[C@@H]([C@H]([C@@H]([C@H](O7)CO)O)O)O)O)C8=CC=C(C=C8)O)O)O[C@H]9[C@@H]([C@H]([C@@H]([C@H](O9)CO)O)O)O 1625

COC(=O)C1CC23CCCN4C2C5(C1(CC3)N(C6=CC=CC=C65)C(=O)OC)CC4 6922

COC(=O)CCCCCCCC(=O)OC 2505

COC(CCC1=CC=C(C=C1)O)CC(=O)CCC2=CC=CC=C2 5425

COC[C@H]1CC2=CC3=C(C=C2[C@H]([C@@H]1COC)C4=CC5=C(C=C4)OCO5)OCO3 8876

COC1=C(C(=C(C=C1)C2CC3=C(C=C(C=C3)O)OC2)O)OC 4374

COC1=C(C(=C(C=C1)OC)OC)C2=CC(=O)C3=C(O2)C=CC(=C3OC)OC 6627

COC1=C(C(=C2C(=C1)C=CC(=O)O2)O)O 3128

COC1=C(C(=C2C(=C1)C=CC(=O)O2)O)OC 3129

COC1=C(C(=C2C(=C1)C=CC(=O)O2)O[C@H]3[C@H]([C@@H]([C@@H]([C@@H](O3)CO)O)O)O)O 3130

COC1=C(C(=C2C(=C1)C=CC(=O)O2)OC)O 4323

COC1=C(C(=C2C(=C1)OC(=CC2=O)C3=CC(=C(C=C3)O)O)O)OC 1534

COC1=C(C(=C2C(=C1)OC(=CC2=O)C3=CC(=C(C=C3)O[C@@H]4[C@H]([C@@H]([C@H]([C@@H](O4)CO)O)O)O)O)O)OC 1535

COC1=C(C(=C2C(=C1)OC(=CC2=O)C3=CC=C(C=C3)O)O)[C@H]4C([C@H]([C@@H](C(O4)CO)O)O)O 8190

COC1=C(C(=C2C(=C1)OC(=CC2=O)C3=CC=C(C=C3)O)O)OC 1536

COC1=C(C(=C2C(=C1)OC3=CC(=C(C=C3C2=O)O)O)O)O[C@H]4[C@@H]([C@H]([C@@H]([C@H](O4)CO)O)O)O 3835

COC1=C(C(=C2CCCCC(=O)CCC3=CC(=C(C=C3)O)C1=C2)O)OC 5910

COC1=C(C=C(C=C1)/C=C/C(=O)OCC(=O)C(C2=CC(=C(C=C2)O)O)OC)O 1494

COC1=C(C=C(C=C1)/C=C/C(=O)OCC(=O)CC2=CC(=C(C=C2)O)O)O 1492

COC1=C(C=C(C=C1)[C@@H]2[C@H]([C@@H]([C@H](N2)CO)O)O)O 7306

COC1=C(C=C(C=C1)[C@@H]2[C@H]([C@H](CO2)CC3=CC(=C(C=C3)O)OC)CO)OC 1967

COC1=C(C=C(C=C1)[C@H]2[C@@H]3[C@@H](CC4=CC5=C(C=C24)OCO5)COC3=O)OC 5803

COC1=C(C=C(C=C1)[C@H]2[C@H]3C4=C(O2)C=CC(=C4)[C@@H]5CC(=O)NCCCN(C3=O)CCCCNCCCN5)OC 2706

COC1=C(C=C(C=C1)[C@H]2[C@H]3CO[C@@H]([C@H]3CO2)C4=CC(=C(C(=C4)OC)OC)OC)OC 5166

COC1=C(C=C(C=C1)C[C@H]2COC(=O)[C@H]2CC3=CC(=C(C=C3)OC)OC)OC 2520

COC1=C(C=C(C=C1)C2=C(C(=O)C3=C(C(=C(C=C3O2)O[C@H]4C([C@H]([C@@H](C(O4)CO)O)O)O)OC)O)OC)O 1368

COC1=C(C=C(C=C1)C2=C(C(=O)C3=C(C(=C(C=C3O2)OC)OC)O)OC)OC 638

COC1=C(C=C(C=C1)C2=C(C(=O)C3=C(C=C(C=C3O2)O)O)O)O 8253

COC1=C(C=C(C=C1)C2=C(C(=O)C3=C(C=C(C=C3O2)OC)O)OS(=O)[O-])OC.[Na+] 7265

COC1=C(C=C(C=C1)C2=CC(=O)C3=C(C=C(C=C3O2)OC)O)OC 4056

COC1=C(C=C(C=C1)C2=CC(=O)C3=CC=CC=C3O2)OC 2492

COC1=C(C=C(C=C1)CC2COC(=O)C2CC3=CC4=C(C=C3)OCO4)OC 4718

COC1=C(C=C(C=C1)Cl)C(=O)NCCC2=CC=C(C=C2)S(=O)(=O)NC(=O)NC3CCCCC3 11093

COC1=C(C=C(C2=C1OC(=C(C2=O)OC)C3=CC=CC=C3)O)O 3459

COC1=C(C=C2C(=C1)C(=O)CC(O2)C3=CC(=C(C=C3)O)O)O 8150

COC1=C(C=C2C(=C1)C=C(O2)C3=CC(=CC(=C3)O)O)OC 5796

COC1=C(C=C2C(=C1)C3COC4=C(C3O2)C=CC(=C4)O)O 4698

COC1=C(C=C2C(=C1)NC(=O)O2)OC 6668

COC1=C(C=C2C(=C1O)C(=O)C(=C(O2)C3=CC(=C(C=C3)O)O)O)O[C@H]4[C@@H]([C@H]([C@@H]([C@H](O4)CO)O)O)O 10149

COC1=C(C=C2C(=C1O)C(=O)C=C(O2)OC3=CC=C(C=C3)O)O 1233

COC1=C(C=C2C(=C1O)C(=O)CC(O2)C3=CC(=C(C=C3)O)O)O 11002

COC1=C(C=C2C(=C1O)C(=O)CC(O2)C3=CC=C(C=C3)O)O 8691

COC1=C(C=C2C(=CC(=O)OC2=C1)C3=CC=CC=C3)O 1953

COC1=C(C=C2C(=CCOC2=C1)C3=CC=CC=C3)O 1952

COC1=C(C=C2C(C(C(CC2=C1)(CO)O)CO)C3=CC(=C(C=C3)O)OC)O 1917

COC1=C(C=C2C[C@H]3C4=C(C2=C1)C(=C(C=C4CCN3)O)OC)O 4821

COC1=C(C=C2C=CC3=CC(=C(C(=C3C2=C1)OC)OC)O[C@H]4[C@@H]([C@H]([C@@H]([C@H](O4)CO)O)O)O)O 4103

COC1=C(C=C2CCC3=C(C2=C1OC)C=CC(=C3)O)O 10714

COC1=C(C=CC(=C1)/C=C/C(=O)O[C@H]2[C@@H]([C@H](O[C@@]2(CO)O[C@@H]3[C@@H]([C@H]([C@@H]([C@H](O3)CO)OC(=O)C4=CC=CC=C4)O)O)CO)O)O 7350

COC1=C(C=CC(=C1)/C=C/C(=O)O[C@H]2[C@@H]([C@H](O[C@@]2(CO)O[C@@H]3[C@@H]([C@H]([C@@H]([C@H](O3)COC(=O)C4=CC=CC=C4)O)O)O)CO)O)O 7351

COC1=C(C=CC(=C1)/C=C/C(=O)OCC(=O)C(C2=CC(=C(C=C2)O)O)OC)O 1495

COC1=C(C=CC(=C1)/C=C/C(=O)OCC(=O)CC2=CC(=C(C=C2)O)O)O 1493

COC1=C(C=CC(=C1)[13CH2][C@@H](CO)[C@@H]([13CH2]C2=CC(=C(C=C2)O)OC)[13CH2]O)O 10981

COC1=C(C=CC(=C1)[C@@H]2[C@@H](OC3=C4C(=CC(=C3O2)OC)C=CC(=O)O4)CO)O 1590

COC1=C(C=CC(=C1)[C@@H]2[C@]3(CO[C@@H]([C@]3(CO2)O)C4=CC(=C(C=C4)O)OC)O)O 7044

COC1=C(C=CC(=C1)[C@@H]2[C@H](C(=CC3=C2C(=C(C=C3)O)OC)C=O)CO)O 9054

COC1=C(C=CC(=C1)[C@@H]2CC(=O)C3=C(C=C(C=C3O2)OC4C(C(C(C(O4)CO)O)O)O)O)OC5C(C(CO5)(CO)O)O 9034

COC1=C(C=CC(=C1)C(=O)CO)O 2446

COC1=C(C=CC(=C1)C(=O)O)[O-] 9440

COC1=C(C=CC(=C1)C(=O)OC2[C@H]([C@@H](C(O[C@H]2C3=C(C=C(C4=C3OC(=CC4=O)C5=CC=C(C=C5)O)O)O)CO)O)O)O 8923

COC1=C(C=CC(=C1)C(=O)OCC23C4C(C=COC4OC5C(C(C(C(O5)CO)O)O)O)C(C2O3)O)O 4720

COC1=C(C=CC(=C1)C2[C@H](C3=CC(=CC(=C3O2)OC)C(C(CO)O)O)CO)O 2425

COC1=C(C=CC(=C1)C2=C(C(=O)C3=C(O2)C(=C(C(=C3O)OC)O)OC)O)O 4920

COC1=C(C=CC(=C1)C2=C(C(=O)C3=C(O2)C(=C(C=C3O)O)OC)OC)O 8700

COC1=C(C=CC(=C1)C2=CC(=O)C3=C(O2)C(=C(C(=C3O)O)OC)OC)O 8574

COC1=C(C=CC(=C1)C2=COC3=CC(=C(C(=C3C2=O)O)OC)O)O 4867

COC1=C(C=CC(=C1)O)CCCC2=CC=C(C=C2)O 1014

COC1=C(C=CC(=C1O)/C=C/C2=CC(=CC(=C2)O)O)O 8982

COC1=C(C=CC2=CC3=[N+](CCC4=CC5=C(C=C43)OCO5)C=C21)O.[Cl-] 8524

COC1=C(C2=C(C(=C1)O)C(=O)C3=C(O2)C=CC(=C3)O)OC 2437

COC1=C(C2=C(C(=C1)O[C@H]3[C@@H]([C@H]([C@@H]([C@H](O3)CO)O)O)O)C(=O)C=C(O2)C4=C(C(=C(C=C4)O)OC)OC)OC 11242

COC1=C(C2=C(C=C1)C(=O)C3=C(C2=O)C=CC(=C3)O)O 2447

COC1=C(C2=C(CC3C4=C2C5=C(C=C4CCN3)OCO5)C=C1)O 4815

COC1=C(C2=C[N+]3=C(C=C2C=C1)C4=CC5=C(C=C4CC3)OCO5)O 899

COC1=C(C2=C3C(CC4=CC(=C(C=C42)OC)O)NCCC3=C1)OC 4822

COC1=C(OC2=CC(=C(C(=C2C1=O)O)O)O)C3=CC(=C(C=C3)O)O 6622

COC1=C(OC2=CC(=CC(=C2C1=O)O)O[C@H]3C([C@H]([C@@H](C(O3)C(=O)O)O)O)O)C4=CC=C(C=C4)O 994

COC1=C2C(=CC3=C1OCO3)CCN4C2(CC5=C(C4)C6=C(C=C5)OCO6)CO 9275

COC1=C2C(=O)CC(OC2=C3[C@@H]4C[C@@H](O[C@@H]([C@H]4[C@@H](OC3=C1)C5=CC=C(C=C5)O)C6=CC=C(C=C6)O)CCC7=CC=C(C=C7)O)C8=CC=C(C=C8)O 2713

COC1=C2C(=O)CC(OC2=CC(=C1)O)C3=C(C=CC=C3O)O 8692

COC1=C2C=CC3=CC(=C(C(=C3C2=C(C=C1)O)OC)OC)O 10718

COC1=C2C=COC2=CC3=C1C(=O)C(=CO3)C4=CC5=C(C=C4)OCO5 3210

COC1=C2C3=CC(=C1)CCCC[C@H](O2)CCC4=CC3=C(C=C4)O 5911

COC1=C2CN3CCC4(C3CC(C=C4)O)C2=CC5=C1OCO5 7004

COC1=CC(=C(C(=C1)OC2C(C(C(C(O2)CO)O)O)O)C(=O)CCC3=CC=C(C=C3)O)O 672

COC1=CC(=C(C(=C1C(=O)/C=C/C2=CC=C(C=C2)O)O)[C@@H]3C[C@@H](O[C@@H](C3)C4=CC=C(C=C4)O)CCC5=CC=C(C=C5)O)O 2712

COC1=CC(=C(C=C1)C2=C(C3=C(O2)C=C(C=C3)OC)C=O)O 5324

COC1=CC(=C(C=C1)C2=CC3=C(O2)C=C(C=C3)O)O 975

COC1=CC(=C(C=C1)CCCC2=CC(=C(C=C2)O)OC)OC 1017

COC1=CC(=C(C=C1)CCCC2=CC(=C(C=C2)OC)O)O 1016

COC1=CC(=C(C=C1)CCCC2=CC=C(C=C2)O)O 1013

COC1=CC(=C(C=C1)OC)C2=CC(=O)C3=C(C=C(C=C3O2)OC)O 4057

COC1=CC(=C(C=C1)OC)C2=CC(=O)C3=C(O2)C4=C(C=C3)OC=C4 5733

COC1=CC(=C(C=C1)OC)C2=CC(=O)C3=CC=CC=C3O2 2491

COC1=CC(=C(C=C1C=O)OC)OC 667

COC1=CC(=C(C=C1C2=COC3=CC(=CC(=C3C2=O)O)O)OC)O 6325

COC1=CC(=C(C=C1C2CCC2C3=CC(=C(C=C3OC)OC)OC)OC)OC 6609

COC1=CC(=C(C2=C1C3=C(C=C2)C=C(C=C3)O)C4=C5CCC6=C(C5=C(C=C4O)OC)C=CC(=C6)O)O 965

COC1=CC(=C2C(=C1)C=CC3=CC(=C(C=C32)O)OC)OC 837

COC1=CC(=C2C(=C1)C3=CC(=C(C=C3N2)O)C=O)OC 1569

COC1=CC(=C2C(=C1)CCC3=C2C(=CC=C3)O)O 5402

COC1=CC(=C2C(=C1)CCC3=C2C=CC(=C3)O)OC 6358

COC1=CC(=C2C(=C1)OC(=C(C2=O)OC)C3=CC(=C(C=C3O)OC)O)O 10163

COC1=CC(=C2C(=C1)OC(=C(C2=O)OC)C3=CC=C(C=C3)O[C@H]4C(C([C@@H](C(O4)CO)O)O)O)O 1177

COC1=CC(=C2C(=C1)OC(=C(C2=O)OS(=O)[O-])C3=CC(=C(C=C3)O)OS(=O)[O-])O.[Na+].[Na+] 7262

COC1=CC(=C2C(=C1)OC(=CC2=O)C3=CC(=C(C(=C3)OC)OC)OC)OC 6630

COC1=CC(=C2C(=C1)OC(C(C2=O)O)C3=CC=CC=C3)O 343

COC1=CC(=C2C(=C1)OC3=CC(=C(C=C3C2=O)OC)O)O 2436

COC1=CC(=C2C(=O)C[C@H](OC2=C1)C3=CC(=C(C=C3)O)O)O 5549

COC1=CC(=C2C(=O)C[C@H](OC2=C1)C3=CC=CC=C3O)O 2373

COC1=CC(=CC(=C1)/C=C/C2=CC=CC=C2)OC 2502

COC1=CC(=CC(=C1)/C=C\C2=CC=C(C=C2)O)OC 7195

COC1=CC(=CC(=C1)O)CCC2=CC(=CC=C2)O 838

COC1=CC(=CC(=C1)O)CCC2=CC=CC=C2O 839

COC1=CC(=CC(=C1[O-])OC)C2=CC(=O)C3=C(C=C(C=C3O2)O)O 8651

COC1=CC(=CC(=C1C(=O)/C=C/C2=C(C=CC=C2O)O)O)O 8689

COC1=CC(=CC(=C1O)OC)/C=C/C(=O)OC[C@@H]2[C@H]([C@@H]([C@H]([C@H](O2)O[C@]3([C@H]([C@@H]([C@H](O3)CO)O)OC(=O)/C=C/C4=CC(=C(C=C4)O)OC)CO)O)O[C@H]5[C@@H]([C@H]([C@@H]([C@H](O5)CO)O)O)O)O 7353

COC1=CC(=CC(=C1O)OC)[C@@H]2[C@@H]3[C@@H](CC4=C(C5=C(C=C24)OCO5)O)COC3=O 9380

COC1=CC(=CC(=C1O)OC)[C@@H]2[C@H](OC3=C(O2)C=CC4=C3OC(=O)C=C4)CO 1969

COC1=CC(=CC(=C1O)OC)[C@H]2[C@@H]3[C@@H](CC4=CC5=C(C=C24)OCO5)COC3=O 2175

COC1=CC(=CC(=C1O)OC)[C@H]2[C@@H]3C(CC4=CC5=C(C=C24)OCO5)COC3=O 9464

COC1=CC(=CC(=C1O)OC)CCC2=CC(=C(C=C2)O)OC 5813

COC1=CC(=CC(=C1O[C@H]2[C@@H]([C@H]([C@@H]([C@H](O2)CO)O)O)O)OC)C3C4COC(C4CO3)C5=CC(=C(C=C5)O[C@H]6[C@@H]([C@H]([C@@H]([C@H](O6)CO)O)O)O)OC 10220

COC1=CC(=CC(=C1OC(CO)C(C2=CC(=C(C=C2)O[C@H]3[C@@H]([C@H]([C@@H]([C@H](O3)CO)O)O)O)OC)O)OC)/C=C/CO 1559

COC1=CC(=CC(=C1OC)O)C2=CC(=O)C3=C(C(=C(C=C3O2)O)OC)O 8699

COC1=CC(=CC(=C1OC)O)C2=CC(=O)C3=C(C(=C(C=C3O2)OC)OC)O 2473

COC1=CC(=CC(=C1OC)O)C2=COC3=CC4=C(C(=C3C2=O)O)OCO4 2312

COC1=CC(=CC(=C1OC)OC)/C=C/C(=O)O[C@H]2[C@@H]([C@H](O[C@@]2(CO)O[C@@H]3[C@@H]([C@H]([C@@H]([C@H](O3)COC(=O)/C=C/C4=CC(=C(C=C4)O)OC)O)O)O)CO)O 7349

COC1=CC(=CC(=C1OC)OC)[C@@H]2[C@H]3CO[C@@H]([C@H]3CO2)C4=CC5=C(C=C4)OCO5 670

COC1=CC(=CC(=C1OC)OC)[C@H]([C@@H]2[C@H](COC2=O)CC3=CC4=C(C=C3)OCO4)O[C@H]5[C@@H]([C@H]([C@@H]([C@H](O5)CO)O)O)O 11820

COC1=CC(=CC(=C1OC)OC)[C@H]2[C@@H]3[C@@H](CC4=C(C5=C(C=C24)OCO5)O)COC3=O 11172

COC1=CC(=CC(=C1OC)OC)C2=CC(=O)C3=C(C(=C(C=C3O2)O)OC)O 636

COC1=CC(=CC(=C1OC)OC)C2=CC(=O)C3=C(O2)C(=C(C(=C3O)OC)OC)OC 3248

COC1=CC(=CC(=C1OC)OC)C2=CC(=O)C3=C(O2)C(=C(C=C3OC)OC)OC 818

COC1=CC(=CC(=C1OC)OC)C2C3C(COC3=O)C(C4=CC5=C(COO5)C=C24)O 6936

COC1=CC(=CC2=C1OC(C2CO)C3=CC(=C(C=C3)O)OC)C=CCOC4C(C(C(C(O4)COC(=O)C(=C)CCO)O)O)O 9137

COC1=CC(=CC2=C1OCO2)C[C@H]3CO[C@@H]([C@@H]3CC4=CC(=C(C(=C4)OC)OC)OC)O 5399

COC1=CC(=O)C2=C(C1=O)C3=CC(=C(C=C3C(=C2)C4=CC=CC=C4)O)OC 4811

COC1=CC(=O)C2=CC[C@H](O[C@@]23[C@@]1(C4=C(C(=C5C(=C4O3)[C@@H](C[C@H](O5)C6=CC=CC=C6)O)OC)OC)OC)C7=CC=CC=C7 2165

COC1=CC(=O)OC(=C1)/C=C/C2=CC=CC=C2 2110

COC1=CC(=O)OC(C1)/C=C/C2=CC(=C(C=C2)OC)OC 5407

COC1=CC=C(C=C1)[C@@H]2[C@@H](C(=O)C3=C(C=C(C=C3O2)O)O)C4[C@H](OC5=CC(=CC(=C5C4=O)O)O)C6=CC=C(C=C6)OC 1405

COC1=CC=C(C=C1)[C@@H]2[C@H](C3=C(O2)C=C4C(=C3O)C(=O)CC(O4)C5=CC=C(C=C5)O)C(=O)C6=C(C=C(C=C6)O)O 208

COC1=CC=C(C=C1)C2=COC3=C(C2=O)C(=CC=C3)O 6506

COC1=CC=C(C=C1)C2=COC3=CC(=C(C(=C3C2=O)O)OC)O 4212

COC1=CC=C(C=C1)CC(=O)OC 5606

COC1=CC=C(C=C1)CCNC(=O)/C=C/C2=CC3=C(C=C2)OCO3 611

COC1=CC=C(O1)C=O 5410

COC1=CC=CC=C1/C=C/C(=O)O 10633

COC1=CC=CC=C1[C@@H]2CC(=O)C3=C(C4=C(C=C3O2)OCO4)OC 11267

COC1=CC=CC2=C1COO2 5385

COC1=CC=CC2=C1N(C34C25CCN6C5C(CCC6)(CC3)CC4C(=O)OC)C=O 8937

COC1=CC=CC2=C1NC3=C[N+](=CC(=C23)OC)C(=O)CCC(=C)C4=NC=C(C5=C4NC6=C5C=CC=C6OC)OC 6810

COC1=CC=CC2=C1NC3=C2C(=CN=C3C=C)OC 4682

COC1=CC=CC2=C1NC3=C2C(=CN=C3CCN4C=CC5=C6C4=CC(=O)C(=O)N6C7=CC=CC=C57)OC 6806

COC1=CC=CC2=C1OC3=C(C2=O)C(=CC(=C3OC)O)O 2435

COC1=CC=CN1 10974

COC1=CC2=C(C=C1)C(=O)C(=C(O2)C3=CC=C(C=C3)O)O 5422

COC1=CC2=C(C=C1)C(=O)C=C(O2)C3=C(C=C(C=C3)OC)OC 8715

COC1=CC2=C(C=C1)C3=C(C=C(C=C3CC2)OC)OC 8713

COC1=CC2=C(C=C1)C3=C(CN4CCCC4C3O)C5=CC(=C(C=C52)O)OC 8818

COC1=CC2=C(C=C1)C3=C(CO2)C4=CC5=C(C=C4O3)OCO5 3076

COC1=CC2=C(C=C1)C3=C4C(=CC5=C3OCO5)C=CN=C4C2=O 741

COC1=CC2=C(C=C1)C3=NC=CC4=C3C(=C(C(=C4OC)OC)N)C2=O 1999

COC1=CC2=C(C=C1)C3=NC=CC4=CC(=C(C(=C43)C2=O)NCCC5=CC=C(C=C5)O)OC 1998

COC1=CC2=C(C=C1)NC=C2CCNC(=O)/C=C\C3=CC=C(C=C3)O 1370

COC1=CC2=CC(=CN=C2C=C1)[C@@H]([C@@H]3CC4CCN3C[C@@H]4C=C)O 7275

COC1=CC2=CC(=CN=C2C=C1)[C@H]([C@H]3CC4CCN3C[C@@H]4C=C)O 7273

COC1=CC2=NC=CC3=C2N(C1=O)C4=CC=CC=C34 5392

COC1=CC23CCCN2CCC4=CC5=C(C=C4C3(C1O)O)OCO5 3919

COC1=CN=C(C2=C1C3=CC=CC=C3N2)C(=O)C(CC4=CC=CC5=C4NC6=CN=CC(=C56)OC)CO 6803

COC1=CN=C(C2=C1C3=CC=CC=C3N2)C4CCCC5=[N+]4C=C(C6=C5NC7=CC=CC=C76)OC.[Cl-] 4681

COC1=CN=C(C2=C1C3=CC=CC=C3N2OC)CCN4C=CC5=C6C4=CC(=O)C(=O)N6C7=CC=CC=C57 6807

COC1C(C(C(C(C1O)O)O)O)O 7251

COC1CCC(=O)N1[C@@H]2C=C(CO2)COC(=O)C3=CC=CN3 992

CS(=O)/C=C/CCN=C=S 7320

CS(=O)CCCC/C(=N\OS(=O)(=O)[O-])/S[C@H]1[C@@H]([C@H]([C@@H]([C@H](O1)CO)O)O)O 3409

CSS(=O)CC=C 5466

CSSCC=C 5465
