# Supplementary material for: Data-mining of potential antitubercular activities from molecular ingredients of traditional Chinese medicines
Source: PeerJ. 2014 Jul 17;2:e476. doi: 10.7717/peerj.476 (PMC4106188; doi:10.7717/peerj.476)
Supplement: Table S3 — Supplementary Table 3 shows the 9 molecules which could penetrate the Mycobacterium tuberculosis cell wall. [file peerj-02-476-s003.docx]

| **Compound No.** | **Compound Structure** | **Ingredient name** | **English name** | **Latin name** | **References to their activity as anti tubercular/antimicrobial agents** |
| --- | --- | --- | --- | --- | --- |
| 1. | 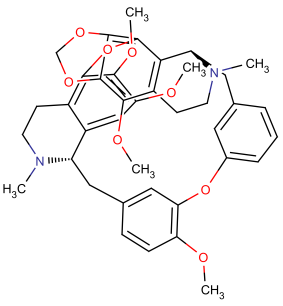 | Thalfinine | Tibetan Meadowrue, Rugose Meadowrue, Low Meadowrue,  Manyleaf Meadowrue, Faber Meadowrue | Thalictrum foetidum,  Thalictrum rugosum,  Thalictrum minus,  Thalictrum foliolosum,  Thalictrum faberi, | Zhou et al., 2011 |
| 2. | 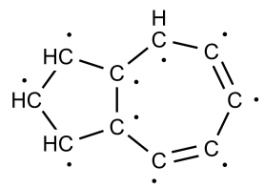 | Azulene | Camphortree,  Common Yarrow | Cinnamomum camphora,  Achillea millefolium | Kurti & Uldrich, 1958 |
| 3. | 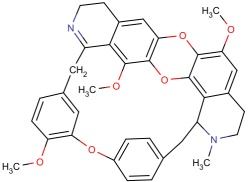 | Menisarine | Japanese Snailseed, Arboreous Coriaria | Cocculus trilobus, Coriaria arborea |  |
| 4. | 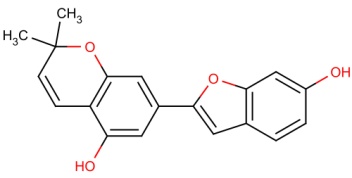 | Moracin d | White Mulberry Leaf,  Common Papermulberry | Morus alba,  Broussonetia papyrifera |  |
| 5. | 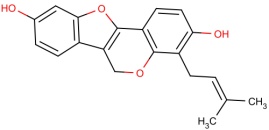 | Erypoegin e | Mountain Immortelle | Erythrina poeppigiana | Sato et al.,2006 |
| 6. | 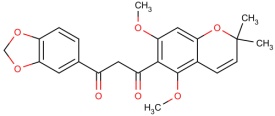 | Pongapinone a | Poongaoil Pongamia | Pongamia pinnata | Mary et al., 2013 |
| 7. | 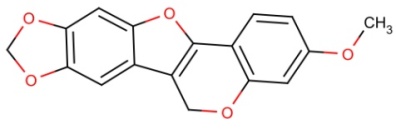 | Flemichapparin b | Climbing Jewelvine | Derris scandens |  |
| 8. | 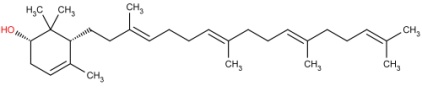 | Achilleol c | Ancients Euphorbia,  Beautiful Garcinia | Euphorbia antiquorum, Garcinia speciosa | Iinuma et al., 1996 |
| 9. | 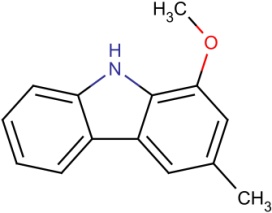 | Murrayafoline a | Taiwan Common Jasminorange, Indian Common Jasminorange, Euchretaleaf Common Jasminorange, Narrowfruit Glycosmis Root | Murraya crenulata, Murraya koenigii, Murraya euchrestifolia [Syn. Clausena euchrestifolia ], Glycosmis stenocarpa | Choi et al., 2006 |
